# Anti-Inflammatory Cembranoids from a Formosa Soft Coral *Sarcophyton cherbonnieri*

**DOI:** 10.3390/md18110573

**Published:** 2020-11-19

**Authors:** Chia-Chi Peng, Chiung-Yao Huang, Atallah F. Ahmed, Tsong-Long Hwang, Jyh-Horng Sheu

**Affiliations:** 1 Department of Marine Biotechnology and Resources, National Sun Yat-sen University, Kaohsiung 804, Taiwan; Chia-Chi.Peng@hki-jena.de (C.-C.P.); huangcy@mail.nsysu.edu.tw (C.-Y.H.); 2 Department of Pharmacognosy, College of Pharmacy, King Saud University, Riyadh 11451, Saudi Arabia; afahmed@ksu.edu.sa; 3 Department of Pharmacognosy, Faculty of Pharmacy, Mansoura University, Mansoura 35516, Egypt; 4 Graduate Institute of Natural Products, College of Medicine, Chang Gung University, Taoyuan 333, Taiwan; htl@mail.cgu.edu.tw; 5 Research Center for Industry of Human Ecology and Graduate Institute of Health Industry Technology, Chang Gung University of Science and Technology, Taoyuan 333, Taiwan; 6 Department of Anesthesiology, Chang Gung Memorial Hospital, Taoyuan 333, Taiwan; 7 Graduate Institute of Natural Products, Kaohsiung Medical University, Kaohsiung 807, Taiwan; 8 Department of Medical Research, China Medical University Hospital, China Medical University, Taichung 404, Taiwan

**Keywords:** *Sarcophyton cherbonnieri*, cembranoid, anti-inflammatory activity, elastase release, superoxide anion generation

## Abstract

The present investigation on chemical constituents of the soft coral *Sarcophyton cherbonnieri* resulted in the isolation of seven new cembranoids, cherbonolides F–L (**1**–**7**). The chemical structures of **1**–**7** were determined by spectroscopic methods, including infrared, one- and two-dimensional (1D and 2D) NMR (COSY, HSQC, HMBC, and NOESY), MS experiments, and a chemical reduction of hydroperoxide by triphenylphosphine. The anti-inflammatory activities of **1**–**7** against neutrophil proinflammatory responses were evaluated by measuring their inhibitory ability toward *N*-formyl-methionyl-leucyl-phenylalanine/cytochalasin B (fMLF/CB)-induced superoxide anion generation and elastase release in primary human neutrophils. The results showed that all isolates exhibited moderate activities, while cherbonolide G (**2**) and cherbonolide H (**3**) displayed a more active effect than others on the inhibition of elastase release (48.2% ± 6.2%) and superoxide anion generation (44.5% ± 4.6%) at 30 µM, respectively.

## 1. Introduction

Series of cembranoidal secondary metabolites from soft corals have been shown to exhibit attractive biological activities including cytotoxicity [1,2,3,4,5,6,7,8,9,10,11,12,13,14] and anti-inflammatory ability [6,7,9,11,13,14,15,16,17,18]. From previous investigations of exploring bioactive natural products from soft corals, many cembranoids were discovered from organisms of the genera *Sarcophyton*, [1,2,3,4,5,6,7,8,16], *Sinularia* [9,10,11,12,17,18], and *Lobophyton* [13,14,15]. In some cases, two cembranoid units could be linked to produce biscembranoids via various reactions [18,19,20,21,22,23,24], marking the high diversity and complexity in chemical structures of cembrane-related soft coral natural products.

**Figure 1:**
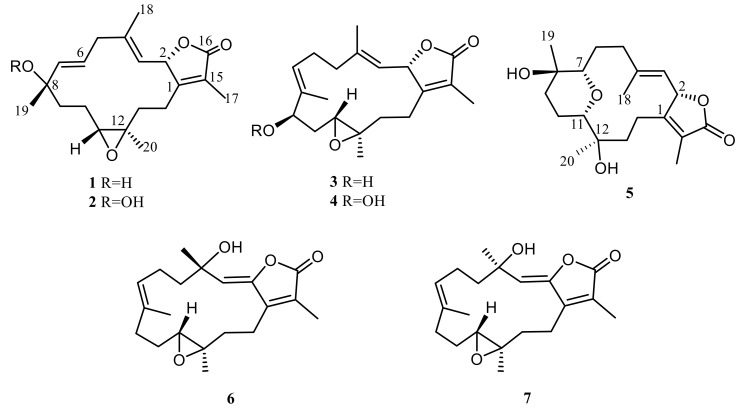
New cembranoids isolated from *Sarcophyton cherbonnieri*.

Many studies have revealed that soft corals of the genus *Sarcophyton* are important sources of various types of natural products, some of them with notable bioactivies [25,26,27,28]. Our previous chemical study on *Sarcophyton cherbonnieri* led to the isolation of six new cembranoids cherbonolides A–E and one biscembranoid bischerbolide peroxide, along with a known compound, isosarcophine [24]. In continuation of our effort on discovery of new and bioactive compounds from marine animals, we further explored the chemical constituents of *S. cherbonnieri*. This investigation again led to the discovery of new cembranoids, cherbonolides F–L (**1–7**). The structures of **1**–**7** (Figure 1) were determined by spectroscopic analysis, including two-dimensional (2D) NMR experiments and a chemical reaction. Compounds **2** and **4** were elucidated as cembranoids possessing an allylic peroxy group. Cembranoids of isosarcophine-type have been reported frequently [24,27,28,29,30].

The screening of the in vitro anti-inflammatory activities through the inhibition of superoxide anion generation and elastase release in *N*-formyl-methionyl-leucyl-phenylalanine/cytochalasin B (fMLF/CB)-induced primary human neutrophils was also performed in order to unveil the anti-inflammatory ability of these compounds. We report herein the isolation, structure determination, and bioactivity of the new metabolites **1**–**7**. 

## 2. Results and Discussion

Solvent-free residue of the ethyl acetate extract of the soft coral *S. cherbonnieri* was separated and further purified by chromatographic methods to yield metabolites **1**–**7**. The structures were established by extensive analyses of MS and NMR spectra (Figures S1–S49, Supplementary Materials). ^13^C- and ^1^H-NMR data which were essential for structure determination of **1**–**7** are listed in Table 1, Table 2 and Table 3.

**Figure 2:**
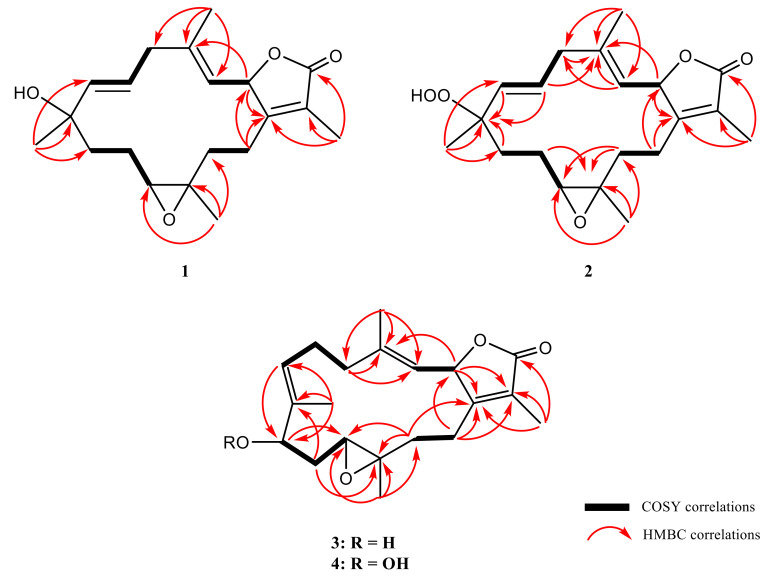
The selected COSY and HMBC correlations of **1**–**4**.

Cherbonolide F (**1**) was obtained as a colorless oil. The molecular formula of **1**, C_20_H_28_O_4_, was established by high-resolution electrospray ionization mass spectrometry (HR-ESI-MS) (*m/z* calculated 355.1880; found 355.1879, [M + Na]^+^), implying seven degrees of unsaturation. The IR spectrum of **1** revealed the absorptions of a hydroxy (ν_max_ 3460 cm^−1^) and a lactonic carbonyl group (ν_max_ 1748 cm^−1^). The ^13^C-NMR spectrum of **1** showed 20 signals which were assigned to four methyls, five *sp^3^* methylenes, two *sp^3^* oxygenated methines, three *sp^2^* methines, and two *sp^3^* and four *sp^2^* nonprotonated carbon atoms (Table 1) with the assistance of distortionless enhancement by polarization transfer (DEPT) spectra. Carbon signals resonating at *δ*_C_ 173.9 (C), 160.5 (C), 123.6 (C), 78.6 (CH), and 9.0 (CH_3_) and proton signals resonating at *δ*_H_ 4.95 (1H, dd, *J* = 10.0, 1.6 Hz) and *δ*_H_ 1.66 (3H, s) were attributed to signals of an α-methyl-α,β-unsaturated-γ-lactone ring by comparing the NMR data of the γ-lactone ring of the known compound isosarcophine (**7**). Signals at *δ*_C_ 61.2 (CH), 60.2 (C), and *δ*_H_ 2.54 (1H, dd, *J* = 6.0, 6.0 Hz) showed the appearance of a trisubstituted epoxide. One trisubstituted and one disubstituted double bond were identified by NMR signals resonating at *δ*_C_ 120.9 (CH), 142.4 (C) and *δ*_H_ 4.54 (1H, dd, *J* = 10.0, 0.8 Hz), and at *δ*_C_ 140.3 (CH), 124.5 (CH) and *δ*_H_ 5.32 (1H, d, *J* = 16.0 Hz) and 5.38 (1H, ddd, *J* = 16.0, 6.8, 6.8 Hz), respectively. ^1^H–^1^H correlation spectroscopy (COSY) correlations established four separate proton sequences, which were connected by heteronuclear multiple bond correlation (HMBC) correlations (Figure 2). Essential HMBC correlations from H-2 to C-1 and C-4, H_2_-14 to C-1 and C-2, H_3_-17 to C-1, C-15, and C-16, H_3_-18 to C-3, C-4, and C-5, H_3_-19 to C-7, C-8, and C-9, and H_3_-20 to C-11, C-12, and C-13 established the 14-membered ring carbon skeleton of **1**, which also indicated the presence of a hydroxyl at C-8.

**Figure 3:**
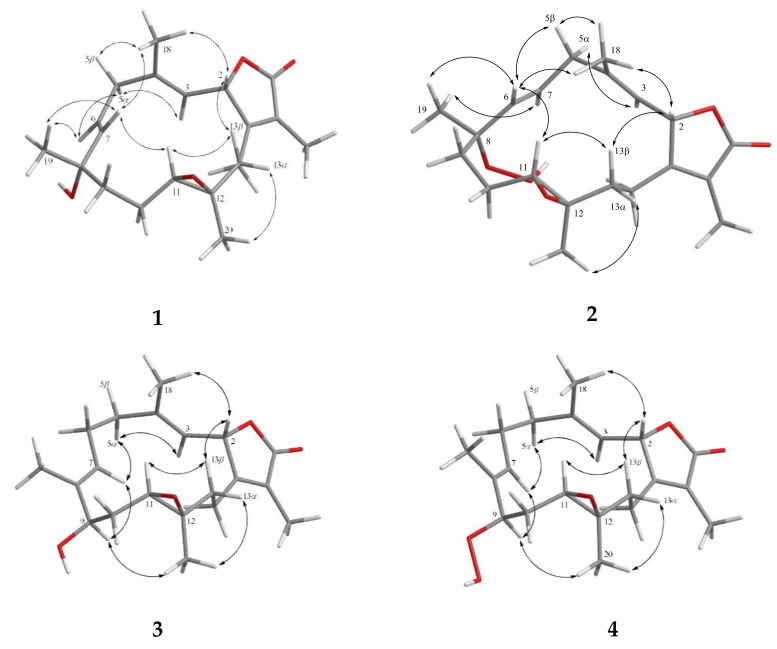
Key NOE correlations for **1**–**4.**

Furthermore, analysis of nuclear Overhauser effect (NOE) correlations was applied to establish the relative configuration of **1**, as shown in Figure 3. It was revealed that H-2 showed NOE correlation with H_3_-18, but not with H-3; therefore, assuming the *β*-orientation of H-2, H_3_-18 should be located on the *β* face. Moreover, H_3_-18 exhibited NOE correlation with H-7, but not with H-6, revealing the β-orientation of H-7 and the *α*-orientation of H-6. Both H-6 and H-7 exhibited NOE interactions with H_3_-19, thus established the *β*-orientation of H_3_-19 as shown in Figure 3. One methylene proton at C-13 exhibited NOE correlation with H-2 and was characterized as H-13*β* (*δ*_H_ 0.99, m), while the other proton was assigned as H-13α (*δ*_H_ 1.49, m). NOE correlations of H-13*β* with H-11 and H-13α with H_3_-20 reflected the *β*-orientation of H-11 and the α-orientation of H_3_-20. The *E* geometries of the trisubstituted C-3/C-4 and C-6/C-7 double bonds were also assigned from the NOE correlations of H_3_-18 (*δ*_H_ 1.35, s) with H-2, but not with H-3, as well as the large coupling constant *J* = 16.0 Hz between H-6 and H-7, and the observed more shielded signal of C-18 (*δ*_C_ 16.7). According to the above observations, the relative configuration of this compound was established. As **1** was isolated together with the previous reported compounds isosarcophine and cherbonolides A−E [24] from the same organism, it should possess the same (2*S*,8*S*,11*R*,12*R*)-configuration from the shared biosynthetic pathway.

The molecular formula of cherbonolide G (**2**) was found to be C_20_H_28_O_5_ by analysis of HR-ESI-MS (*m/z* calculated 371.1829; found 371.1830, [M + Na]^+^), revealing that **2** possesses an additional oxygen atom to that of **1**. Moreover, both **1** and **2** showed the very similar ^1^H- and ^13^C-NMR data (Table 1), except that the chemical shift of C-8 was shifted from *δ*_C_ 71.7 of **1** to *δ*_C_ 83.7 of **2**. The very similar COSY, HMBC (Figure 2), and NOE (Figure 3) correlations of **1** and **2** also revealed the very close structures for both compounds. However, the hydroxy group of **1** at C-8 was replaced by a hydroperoxy group in **2**, with a broad singlet appearing at *δ*_H_ 6.72 and the downfield shift of C-8. Accordingly, the molecular skeleton and the (2*S*,8*S*,11*R*,12*R*)-configuration of **2** were determined.

Cherbonolide H (**3**) has the same molecular formula as that of **1**, as determined by HR-ESI-MS experiment. Moreover, most of the ^1^H–^1^H COSY and HMBC correlations (Figure 2) of **3** were similar to those of isosarcophine except for the presence of a hydroxyl at C-9 leading to the shift of CH-9 to lower field (*δ*_C_ 76.2; *δ*_H_ 3.68), and the shift of C-6/C-7 double bond of **1** to C-7/C-8 double bond of **3**. Analysis of NOE correlations (Figure 3) showed that the *β*-oriented H-2 exhibited NOE interactions with both H_3_-18 and H-13*β*, but not with H-3, assigning the *E*-geometry of the trisubstituted C-3/C-4 double bond. These results, along with the found NOE correlations (Figure 3) of H-13α/ H_3_-20, H_3_-20/H-9, led to the assignment of the α-orientation of H-9.

Cherbonolide I (**4**) was found to contain one additional oxygen atom than **3**, according to HR-ESI-MS experiment. These two compounds also showed very similar ^1^H–^1^H COSY and HMBC correlations, revealing the identical molecular framework of both compounds. NMR data of **3** and **4** were similar (Table 1), except for those of CH-9, suggesting that **4** is possibly the C-9 hydroperoxy derivative of **3**. By analysis of NOE correlations (Figure 3), the *E* geometries of both C-3/C-4 and C-7/C-8 double bonds of **4** and the (2*S*,9*R*,11*R*,12*R*)-configuration were also established. Reduction of **4** by triphenylphosphine yielded **3**, further confirming the structure of **4**.

**Figure 4:**
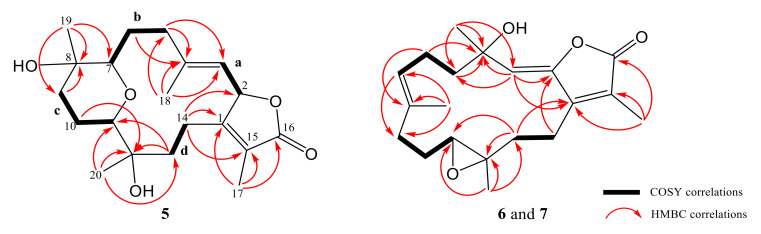
Selected COSY and HMBC correlations of **5**–**7**.

**Figure 5:**
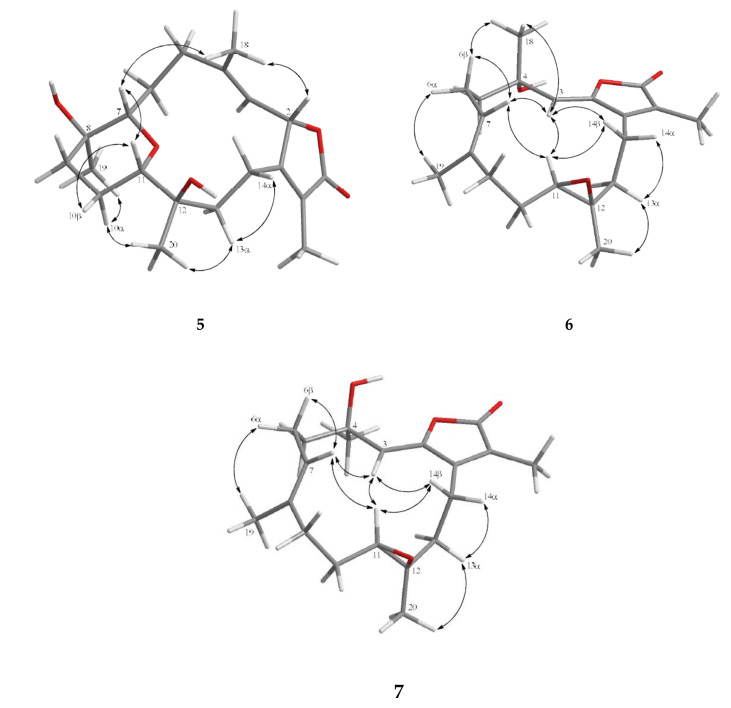
Selected NOE correlations for **5**–**7**.

**Figure 6:**
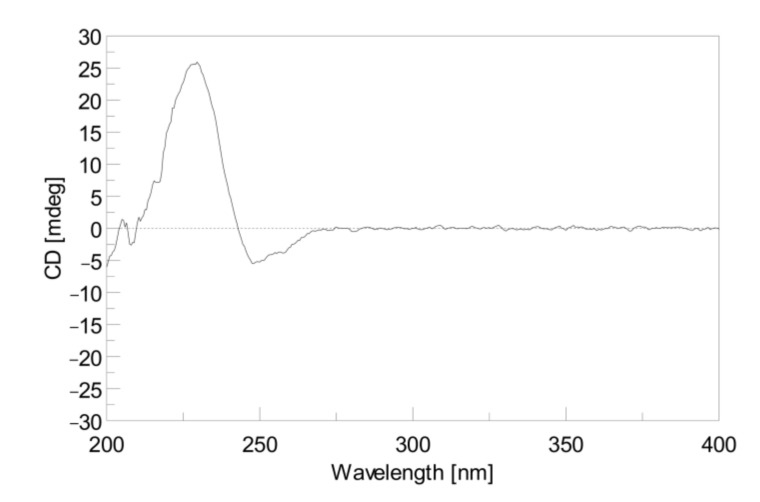
the CD spectrum of **5** (1.2 × 10^−^^4^ M, MeOH).

Cherbonolide J (**5**) was given as a colorless oil with a molecular formula C_20_H_30_O_5_ on the basis of HR-ESI-MS data (*m/z* calculated for C_20_H_30_O_5_Na 373.1986; found 373.1984), revealing six degrees of unsaturation. The IR absorptions at 3443 and 1748 cm^−1^ were due to hydroxy and ester carbonyl groups, respectively. The ^13^C- and ^1^H-NMR spectroscopic data (Table 1 and Table 3) of **5** measured at C_6_D_6_ were very close to a known compound sarcophyolide E [29], and the 2D NMR (COSY, HSQC, and HMBC) correlation analysis revealed that both compounds had the same molecular framework (Figure 4). Detailed analysis of the NOE correlations (Figure 5) showed that both compounds possessed the same relative configuration. However, the [α]${}_{25}^{D}$ values in CHCl_3_ (−6 for **5** and +4.4 for sarcophyolide E) were close but with different signs, suggesting that **5** is the enantiomer of this known compound. The absolute configurations of **5** and sarcophyolide E were deduced by comparison of the circular dichroism (CD) spectroscopic data. As shown in Figure 6, the negative Cotton effect at 247 nm and positive effect at 228 nm for **5** in comparison with the positive and negative Cotton effects at 252 and 226 nm for sarcophyolide E [29], respectively, confirmed that **5** is the newly found enantiomer of sarcophyolide E.

Cherbonolide K (**6**) is a colorless oil which was shown to have the molecular formula C_20_H_28_O_4_ by HR-ESI-MS experiment, appropriate for seven degrees of unsaturation. The infrared (IR) spectrum of **6** showed peaks of hydroxy and estercarbonyl groups at 3444 and 1763 cm^−1^, respectively. ^13^C-NMR data (Table 1) with signals at *δ*_C_ 151.2 (C), 147.2 (C), 116.2 (CH), 72.7 (C), 123.6 (C), 169.5 (C), 9.1 (CH_3_), and 29.9 (CH_3_) and ^1^H NMR data (Table 3) with signals at *δ*_H_ 5.50 (s, 1H), 1.95 (s, 3H), and 1.41 (s, 3H) were attributed to the cembranoidal α-methyl-α,β-unsaturated-γ lactone ring with a conjugated 2,3-double bond that further connected with the methyl and hydroxyl substituted C-4. The above results were supported by HMBC correlations of **6** (Figure 4) from H-3 (*δ*_H_ 5.50) to C-1 (*δ*_C_ 151.2), C-2 (*δ*_C_ 147.2), and C-4 (*δ*_C_ 72.7), H_3_-17 (*δ*_H_ 1.95) to C-1, C-15 (*δ*_C_ 123.6), and C-16 (*δ*_C_ 169.5), and H_3_-18 (*δ*_H_ 1.41) to C-3 (*δ*_C_ 116.2) and C-4 (*δ*_C_ 72.6). The remainder of the structure from C-5 to C-14 was found to be identical to isosarcophine [24]. Thus, the planar structure of **6** was established. Furthermore, the NOE correlation analysis shown in Figure 5 revealed the *α*-orientations of 4-OH and 12-CH_3_, *β*-orientation of H-11, (*Z*)-2,3-double bond, and (*E*)-7,8-double bond. An isomer of **6**, cherbonolide L (**7**), was also subsequently isolated. The metabolite **7** had nearly the same NMR data as **6** except for CH_2_-5 and CH_2_-6. Thus, it can be assumed that **7** is the C-4 epimer of **6**. Analysis of the 2D NMR correlations of **7** (Figure 4 and Figure 6) further supported this assumption.

For the screening of bioactivities, the anti-inflammation activities of **1**–**7** toward inhibition of *N*-formyl-methionyl-leucyl-phenylalanine/cytochalasin B (fMLF/CB)-induced generation of superoxide anion (O_2_^•^^─^) and release of elastase in primary human neutrophils were measured. The results (Table 4) showed that, although none of the isolates exhibited strong inhibitory activities in the assay, **2** and **3** were found to display notable ability to inhibit the elastase release (48.2% ± 6.2%) and superoxide anion generation (44.5% ± 4.6%) at 30 μM, respectively. In comparison with (+)-isosarcophine, cherbonolides A–E, and bischerbonolide peroide discovered previously from *S. cherbonnieri* [24], it was found that, although **2** and **3** exhibited weaker activities than bischerbonolide peroxide, they displayed comparable activities to those of cherbonolides A and C. In general, allylic oxidation at the 7,8-double bond of (+)-isosarcophine might be able to produce derivatives with stronger bioactivities.

## 3. Materials and Methods

### 3.1. General Experimental Procedures 

Values of the specific optical rotation of the isolates were measured with a JASCO P-1020 polarimeter (JASCO Corporation, Tokyo, Japan). Infrared spectra were recorded using a JASCO FT/IR-4100 infrared spectrophotometer (JASCO Corporation, Tokyo, Japan). The CD spectrum was recorded on a Jasco J-815 circular dichroism (CD) spectropolarimeter (JASCO, Tokyo, Japan) in MeOH. ^1^H- and ^13^C-NMR spectra were acquired on a Varian 400MR FT-NMR (or Varian Unity INOVA500 FT-NMR) instrument (Varian Inc., Palo Alto, CA, USA) at 400 MHz (or 500 MHz) and 100 MHz (or 125 MHz), respectively, in CDCl_3_ or C_6_D_6_. LR-ESI-MS and HR-ESI-MS experiments were carried out using a Bruker APEX II (Bruker, Bremen, Germany) mass spectrometer. Silica gel (230–400 mesh) was used as the adsorbent for normal-phase column chromatography. Thin-layer chromatography (TLC) analyses were performed with precoated silica gel plates (Kieselgel 60 F-254, 0.2 mm, Merck, Darmstadt, Germany). Further purification of impure fractions or compounds was further achieved by high-performance liquid chromatography on a Hitachi L-7100 HPLC instrument (Hitachi Ltd., Tokyo, Japan) with a Merck Hibar Si-60 column (250 mm × 21 mm, 7 μm; Merck, Darmstadt, Germany) and on a Hitachi L-2455 HPLC apparatus (Hitachi, Tokyo, Japan) with a Supelco C18 column (250 mm × 21.2 mm, 5 μm; Supelco, Bellefonte, PA, USA).

### 3.2. Animal Materials

The marine organism *S. cherbonnieri* was collected and preserved as described previously [24].

### 3.3. Extraction and Isolation

By using the procedure reported previously, 1.2 kg (wet weight) of organism *S. cherbonnieri* was dehydrated, minced, extracted, and concentrated to afford 10.2 g of residue. The residue was fractionated by chromatography to yield 19 fractions [24]. Fraction 10, eluting with *n*-hexane–acetone (4:1), was further purified over silica gel using *n*-hexane–acetone (6:1) to afford seven subfractions (A1–A7). Subfraction A3 was further separated by reverse-phase HPLC using acetonitrile–H_2_O (1:1.1) to afford **2** (1.4 mg). Subfraction A4 was purified by reverse-phase HPLC using acetonitrile–H_2_O (1:1.2) to afford **4** (8.8 mg), and subfraction A6 was purified by reverse-phase HPLC acetonitrile–H_2_O (2:1) to afford **5** (3.1 mg). Fractions 11 and 12, obtained by eluting with *n*-hexane–acetone 3:1 and 2:1, respectively, were combined and further eluted with acetone by a Sephadex LH-20 column to afford six subfractions (B1–B6). The purification of subfractions B4 and B5 using reverse-phase HPLC by elution of acetonitrile–H_2_O (1:1.3) and MeOH–H_2_O (3:2) afforded **6** (12.4 mg) and **7** (33.1 mg), respectively. Fraction 13, eluting with *n*-hexane–acetone (1:1), was purified by eluting with acetone on Sephadex LH-20 to yield five subfractions (C1–C5). Subfraction C2 was further separated by reverse-phase HPLC using acetonitrile–H_2_O (1:1.4) to afford **1** (3.3 mg) and **3** (10.8 mg).

Cherbonolide F (**1**): colorless oil; [α]${}_{25}^{D}$ +177 (*c* 0.50, CHCl_3_); IR (neat) ν_max_ 3460, 2967, 2928, 2864, 1748, 1677, 1452, 1385, 1096, 984, and 755 cm^–1^; for ^13^C- and ^1^H-NMR data (400 MHz; C_6_D_6_), see Table 1 and Table 2; ESI-MS *m/z* 355 [M + Na]^+^; HR-ESI-MS *m/z* 355.1879 [M + Na]^+^ (calculated for C_20_H_28_O_4_Na, 355.1880). 

Cherbonolide G (**2**): colorless oil; [α]${}_{25}^{D}$ +25 (*c* 0.33, CHCl_3_); IR (neat) ν_max_ 3419, 2925, 2855, 1748, 1678, 1454, 1387, 1096, 987, and 755 cm^–1^; for ^13^C- and ^1^H-NMR data (400 MHz; C_6_D_6_), see Table 1 and Table 2; ESI-MS *m/z* 371 [M + Na]^+^; HR-ESI-MS *m/z* 371.1830 [M + Na]^+^ (calculated for C_20_H_28_O_5_Na, 371.1829).

Cherbonolide H (**3**): colorless oil; [α]${}_{25}^{D}$ +41 (*c* 1.00, CHCl_3_); IR (neat) ν_max_ 3445, 2928, 2864, 1747, 1679, 1455, 1387, 1094, 996, and 755 cm^–1^; for ^13^C- and ^1^H-NMR data (400 MHz; C_6_D_6_), see Table 1 and Table 2; ESI-MS *m/z* 355 [M + Na]^+^; HR-ESI-MS *m/z* 355.1878 [M + Na]^+^ (calculated for C_20_H_28_O_4_Na, 355.1880).

Cherbonolide I (**4**): colorless oil; [α]${}_{25}^{D}$ +13 (*c* 1.00, CHCl_3_); IR (neat) ν_max_ 3420, 2925, 2855, 1747, 1541, 1390, 992, and 756 cm^–1^; for ^13^C- and ^1^H-NMR data (500 MHz; C_6_D_6_), see Table 1 and Table 2; ESI-MS *m/z* 371 [M + Na]^+^; HR-ESI-MS *m/z* 371.1828 [M + Na]^+^ (calculated for C_20_H_28_O_5_Na, 371.1829).

Cherbonolide J (**5**): white powder; [α]${}_{25}^{D}$ −6 (*c* 0.50, CHCl_3_); IR (neat) ν_max_ 3443, 2937, 2860, 1755, 1675, 1381, 1076, 990, and 755 cm^–1^; CD (1.2 × 10^−^^4^ M, MeOH) λ_max_ (Δε ) 247 (−5.2), and 228 (+26.5) nm; for ^13^C- and ^1^H-NMR data (400 MHz; C_6_D_6_), see Table 1 and Table 3; ESI-MS *m/z* 373 [M + Na]^+^; HR-ESI-MS *m/z* 373.1984 [M + Na]^+^ (calculated for C_20_H_30_O_5_Na, 373.1986).

Cherbonolide K (**6**): yellow oil; [α]${}_{25}^{D}$ +12 (*c* 1.00, CHCl_3_); IR (neat) ν_max_ 3444, 2927, 1763, 1435, 1386, 1241, 1083, 931, and 756 cm^–1^; for ^13^C- and ^1^H-NMR data (400 MHz; CDCl_3_), see Table 1 and Table 3; ESI-MS *m/z* 355 [M + Na]^+^; HR-ESI-MS *m/z* 355.1880 [M + Na]^+^ (calculated for C_20_H_28_O_4_Na, 355.1880).

Cherbonolide L (**7**): yellow oil; [α]${}_{25}^{D}$ +33 (*c* 1.00, CHCl_3_) ; IR (neat) ν_max_ 3445, 2929, 2872, 1752, 1665, 1455, 1384, 1050, 927, and 756 cm^–1^; for ^13^C- and ^1^H-NMR data (400 MHz; CDCl_3_), see Table 1 and Table 3; ESI-MS *m/z* 355 [M + Na]^+^; HR-ESI-MS *m/z* 355.1877 [M + Na]^+^ (calculated for C_20_H_28_O_4_Na, 355.1880).

### 3.4. Reduction of Cherbonolide I (***4***)

The solution of compound **4** (1.4 mg) in diethyl ether (5.0 mL) was added to an excess amount of triphenylphosphine (1.3 mg), and the mixture was stirred at room temperature for 4 h. The solvent of the solution was evaporated under reduced pressure to afford a residue, which was purified by silica gel column chromatography using *n*-hexane–acetone (3:1) as an eluent to yield **3** (1.0 mg, 75%).

### 3.5. In Vitro Anti-Inflammatory Assay

#### 3.5.1. Primary Human Neutrophils

Blood was obtained from the elbow vein of healthy adult volunteers (with ages 20–30). Neutrophils were enriched by means of dextran sedimentation, Ficoll–Hypaque centrifugation, and hypotonic lysis. Neutrophils were incubated in an ice-cold Ca^2+^-free Hank's Balanced Salt Solution (HBSS buffer, pH 7.4) [31]. The research protocol was granted approval by the institutional review board of Chang Gung Memorial Hospital (IRB No: 201601307A3, 20161124-20191123; 201902217A3, 20200501-20240630). All subjects gave their informed consent for inclusion before they participated in the study. The study was conducted in accordance with the Declaration of Helsinki.

#### 3.5.2. Superoxide Anion Generation

Neutrophils (6 × 10^5^ cells·mL^−1^) incubated in HBSS with ferricytochrome *c* (0.5 mg·mL^−1^) and Ca^2+^ (1 mM) at 37 °C were treated with dimethyl sulfoxide (DMSO), as control, or with the tested compound for 5 min. Neutrophils were primed by cytochalasin B (CB, 1 μg·mL^−1^) for 3 min before activating fMLF (100 nM) for 10 min (fMLF/CB). The change in superoxide anion generation was spectrophotometrically measured at 550 nm (U-3010, Hitachi, Tokyo, Japan) [32,33].

#### 3.5.3. Elastase Release

Neutrophils (6 × 10^5^ cells·mL^−1^) incubated in HBSS with MeO-Suc-Ala-Ala-Pro-Val-*p*- nitroanilide (100 μM) and Ca^2+^ (1 mM) at 37 °C were treated with DMSO or the tested compound for 5 min. Neutrophils were challenged by fMLF (100 nM)/CB (0.5 μg·mL^−1^) for 10 min. The change in elastase release was spectrophotometrically measured at 405 nm (U-3010, Hitachi, Tokyo, Japan) [32].

#### 3.5.4. Statistical Analysis

Data were displayed as the mean ± SD, and comparisons were performed by one-way ANOVA with Dunnett analysis. All results were obtained from eight biological replicates. A probability value of 0.05 or less was considered to be significant. The Prism software (Version 5.0, GraphPad Software, San Diego, CA, USA) was used for the statistical analysis.

## 4. Conclusions

Our present examination of the chemical constituents of the soft coral *S. cherbonnieri* led to the discovery of new cembranoid compounds **1**–**7**. All compounds were found to possess anti-inflammatory activity by exhibiting inhibitory effects on the generation of superoxide anion and elastase release in fMLF/CB-induced primary human neutrophils, and cherbonolides G and H (**2** and **3**) were found to be the most active in the inhibition of elastase release and superoxide anion generation, respectively. As the marine environment is an important source of bioactive substances, and due to the high chemical diversity and specimen diversity of the *Sarcophyton* genus [27,28,34,35], it can be expected that new natural products and activities from soft corals of this genus can be continuously discovered in the future.

## Figures and Tables

**Table 1:** ^13^C-NMR spectroscopic data of compounds **1**–**7.**

**Position**	**1 ^a^**	**2 ^a^**	**3 ^a^**	**4** **^c^**	**5 ^a^**	**6** **^d^**	**7 ^d^**
**1**	160.5 (C)	160.4 (C)	159.9 (C)	160.2 (C)	162.4 (C)	151.2 (C)	151.2 (C)
**2**	78.6 (CH) ^b^	78.5 (CH)	77.7 (CH)	78.0 (CH)	79.5 (CH)	147.2 (C)	147.2 (C)
**3**	120.9 (CH)	121.2 (CH)	121.8 (CH)	122.3 (CH)	120.7 (CH)	116.2 (CH)	116.1 (CH)
**4**	142.4 (C)	142.1 (C)	143.2 (C)	143.4 (C)	143.2 (C)	72.7 (C)	72.6 (C)
**5**	41.5 (CH_2_)	41.9 (CH_2_)	38.4 (CH_2_)	38.7 (CH_2_)	36.4 (CH_2_)	42.5 (CH_2_)	42.2 (CH_2_)
**6**	124.5 (CH)	128.6 (CH)	23.9 (CH_2_)	24.4 (CH_2_)	24.7 (CH_2_)	23.1 (CH_2_)	23.2 (CH_2_)
**7**	140.3 (CH)	135.9 (CH)	127.3 (CH)	130.9 (CH)	84.1 (CH)	127.2 (CH)	126.5 (CH)
**8**	71.7 (C)	83.7 (C)	137.1 (C)	133.8 (C)	69.4 (C)	133.9 (C)	133.8 (C)
**9**	39.7 (CH_2_)	35.7 (CH_2_)	76.2 (CH)	88.9 (CH)	40.7 (CH_2_)	36.3 (CH_2_)	36.2 (CH_2_)
**10**	24.3 (CH_2_)	24.2 (CH_2_)	32.4 (CH_2_)	28.4 (CH_2_)	23.5 (CH_2_)	24.4 (CH_2_)	24.3 (CH_2_)
**11**	61.3 (CH)	61.1 (CH)	59.2 (CH)	59.4 (CH)	80.1 (CH)	60.5 (CH)	60.5 (CH)
**12**	60.2 (C)	60.2 (C)	60.1 (C)	60.6 (C)	72.6 (C)	60.2 (C)	60.3 (C)
**13**	35.7 (CH_2_)	35.6 (CH_2_)	36.9 (CH_2_)	37.3 (CH_2_)	37.2 (CH_2_)	35.1 (CH_2_)	35.1 (CH_2_)
**14**	23.2 (CH_2_)	22.9 (CH_2_)	23.6 (CH_2_)	24.0 (CH_2_)	20.2 (CH_2_)	19.6 (CH_2_)	19.8 (CH_2_)
**15**	123.6 (C)	123.6 (C)	123.7 (C)	124.1 (C)	123.4 (C)	123.6 (C)	123.7 (C)
**16**	173.9 (C)	173.9 (C)	173.8 (C)	174.2 (C)	174.4 (C)	169.5 (C)	169.8 (C)
**17**	9.0 (CH_3_)	8.9 (CH_3_)	8.7 (CH_3_)	9.1 (CH_3_)	8.9 (CH_3_)	9.1 (CH_3_)	9.0 (CH_3_)
**18**	16.7 (CH_3_)	16.2 (CH_3_)	14.4 (CH_3_)	14.7 (CH_3_)	16.2 (CH_3_)	29.9 (CH_3_)	29.4 (CH_3_)
**19**	28.0 (CH_3_)	21.6 (CH_3_)	9.6 (CH_3_)	10.3 (CH_3_)	20.4 (CH_3_)	15.3 (CH_3_)	15.5 (CH_3_)
**20**	16.7 (CH_3_)	16.9 (CH_3_)	16.0 (CH_3_)	16.2 (CH_3_)	23.7 (CH_3_)	17.5 (CH_3_)	17.4 (CH_3_)

^a^ Spectra recorded in C_6_D_6_ at 100 MHz at 25 °C. ^b^ Attached protons were deduced by distortionless enhancement by polarization transfer (DEPT) experiments. ^c^ Spectra recorded in C_6_D_6_ at 125 MHz. ^d^ Spectra recorded in CDCl_3_ at 100 MHz.

**Table 2:** ^1^H-NMR spectral data for compounds **1**−**4**.

**Position**	**1 ^a^**	**2 ^a^**	**3 ^a^**	**4** **^b^**
**2**	4.95, dd (10.0, 1.6) ^c^	4.92, dd (10.0, 1.6)	4.99, dd (10.4, 1.6)	4.96, d (10.5)
**3**	4.54, dd (10.0, 0.8)	4.47, d (10.0)	4.49, d (10.4)	4.47, d (10.5)
**5**	2.40, dd (13.2, 6.8)	2.41, dd (13.6, 7.2)	1.84, dd (13.2, 4.4)	1.80, dd (13.5, 4.5)
	2.26, dd (13.2, 6.8)	2.27, dd (13.6, 7.2)	1.92, m	1.91, m
**6**	5.38, ddd (16.0, 6.8, 6.8)	5.47, ddd (16.8, 7.2, 7.2)	1.73, m	1.75, m
			2.03, m	2.02, m
**7**	5.32, d (16.0)	5.35, d (16.8)	4.74, dd (10.0, 1.2)	4.91, d (9.5)
**9**	1.52, m	1.57, m	3.68, dd (11.6, 4.0)	4.06, dd (12.0, 4.0)
	1.59, m	1.59, m		
**10**	1.43, m	1.56, m	1.47, m	1.53, m
	1.71, m	1.56, m	2.16, ddd	2.03, m
**11**	2.54, dd (6.0, 6.0)	2.44, dd (6.4, 6.4)	2.03, m	2.09, dd (10.5, 3.0)
**13**	1.49, m	1.69, m	1.59, dd (13.2, 5.6)	1.56, m
	0.99, m	0.99, m	0.72, ddd (13.2, 13.2, 2.8)	0.69, dd (13.5, 13.5, 2.5)
**14**	1.81, m	1.78, m	1.93, m	1.89, m
	1.67, m	1.68, m	1.49, m	1.43, m
**17**	1.66, s	1.66, s	1.65, s	1.65, s
**18**	1.35, s	1.29, s	1.13, s	1.11, s
**19**	1.05, s	1.19, s	1.37, s	1.37, s
**20**	1.03, s	1.02, s	1.03, s	1.01, s

^a^ Spectra recorded in C_6_D_6_ at 400 MHz at 25 °C. ^b^ Spectra recorded in C_6_D_6_ at 500 MHz at 25 °C. ^c^ Coupling constants (*J* values) in Hz are shown in parentheses.

**Table 3:** ^1^H-NMR spectral data for compounds **5**−**7**.

**Position**	**5** **^a^**	**6 ^b^**	**7 ^b^**
**2**	4.92, d (11.2) ^c^		
**3**	4.85, d (11.2)	5.50, s	5.52, s
**5**	2.07, m	1.83, m	1.94, m
	1.93, m	1.98, m	1.94, m
**6**	1.31, m	2.41, m	2.46, m
	1.70, m	2.21, m	2.14, m
**7**	2.79, dd (10.0, 2.4)	5.26, dd (6.0, 6.0)	5.25, dd (7.2, 7.2)
**9**	1.59, m	2.28, m	2.26, m
	1.31, m	2.08, m	2.06, m
**10**	1.22, m	1.53, m	1.54, m
	1.50, m	1.85, m	1.86, m
**11**	2.96, d (11.2)	2.71, dd (6.8, 5.6)	2.73, dd (7.6, 4.6)
**13**	1.59, m	2.16, m	2.19, m
	1.21, m	1.63, m	1.62, m
**14**	2.16, ddd (12.4, 12.4, 6.4)	2.26, m	2.24, m
	1.59, m	2.42, m	2.45, m
**17**	1.72, s	1.95, s	1.92, s
**18**	1.47, s	1.41, s	1.51, s
**19**	0.94, s	1.66, s	1.65, s
**20**	0.89, s	1.30, s	1.28, s

^a^ Spectra recorded in C_6_D_6_ at 400 MHz at 25 °C. ^b^ Spectra recorded in CDCl_3_ at 400 MHz at 25 °C. ^c^ Coupling constants (*J* values) in Hz are shown in parentheses.

**Table 4:** Inhibitory effects of metabolites **1**–**7** against elastase release and superoxide anion generation in *N*-formyl-methionyl-leucyl-phenylalanine/cytochalasin B (fMLF/CB)-induced primary human neutrophils. IC_50_, half maximal inhibitory concentration.

Compound	Superoxide Anion		Elastase Release	
	IC_50_ (μM) ^a^	Inh ^b^ %	IC_50_ (μM) ^a^	Inh ^b^ %
**1**	>30	11.0 ± 8.7	>30	35.1 ± 10.6 ***
**2**	>30	29.8 ± 9.8 **	>30	48.2 ± 12.5 ***
**3**	>30	44.5 ± 7.9 ***	>30	35.6 ± 10.7 ***
**4**	>30	6.4 ± 7.3	>30	27.6 ± 12.8 **
**5**	>30	6.2 ± 5.5	>30	29.7 ± 11.1 **
**6**	>30	12.9 ± 11.4	>30	16.7 ± 10.2 *
**7**	>30	17.1 ± 11.6 *	>30	27.6 ± 12.0 **
**Idelalisib**	0.07 ± 0.03	102.8 ± 5.4 ***	0.07 ± 0.02	99.6 ± 10.3 ***

^a^ Concentration necessary for 50% inhibition (IC_50_). ^b^ Percentage of inhibition (Inh %) at 30 μM. Results presented as mean ± S.D. The anti-inflammatory assays were performed with eight biological replicates. * *p* < 0.05, ** *p* < 0.01, and *** *p* < 0.001 compared with the control.

## Supplementary Materials

The following are available online at https://www.mdpi.com/1660-3397/18/11/573/s1. HR-ESI-MS, ^1^H-NMR, ^13^C-NMR, DEPT, HMQC, COSY, HMBC, and NOESY spectra of new compounds **1**–**7** are available online at https://www.mdpi.com/1660-3397/18/11/573/s1. Figure S1: HR-ESI-MS spectrum of **1**; Figure S2. ^1^H-NMR spectrum of **1** in C_6_D_6_; Figure S3. ^13^C-NMR spectrum of **1** in C_6_D_6_; Figure S4. HSQC spectrum of **1** in C_6_D_6_; Figure S5. ^1^H–^1^H COSY spectrum of **1** in C_6_D_6_; Figure S6. HMBC spectrum of **1** in C_6_D_6_; Figure S7. NOESY spectrum of **1** in C_6_D_6_; Figure S8. HR-ESI-MS spectrum of **2**; Figure S9. ^1^H-NMR spectrum of **2** in C_6_D_6_; Figure S10. ^13^C-NMR spectrum of **2** in C_6_D_6_; Figure S11. HSQC spectrum of **2** in C_6_D_6_; Figure S12. ^1^H–^1^H COSY spectrum of **2** in C_6_D_6_; Figure S13. HMBC spectrum of **2** in C_6_D_6_; Figure S14. NOESY spectrum of **2** in C_6_D_6_; Figure S15. HR-ESI-MS spectrum of **3**; Figure S16. ^1^H-NMR spectrum of **3** in C_6_D_6_; Figure S17. ^13^C-NMR spectrum of **3** in C_6_D_6_; Figure S18. HSQC spectrum of **3** in C_6_D_6_; Figure S19. ^1^H–^1^H COSY spectrum of **3** in C_6_D_6_; Figure S20. HMBC spectrum of **3** in C_6_D_6_; Figure S21. NOESY spectrum of **3** in C_6_D_6_; Figure S22. HR-ESI-MS spectrum of **4**; Figure S23. ^1^H-NMR spectrum of **4** in C_6_D_6_; Figure S24. ^13^C-NMR spectrum of **4** in C_6_D_6_; Figure S25. HSQC spectrum of **4** in C_6_D_6_; Figure S26. ^1^H-^1^HCOSY spectrum of **4** in C_6_D_6_; Figure S27. HMBC spectrum of **4** in C_6_D_6_; Figure S28. NOESY spectrum of **4** in C_6_D_6_; Figure S29. HR-ESI-MS spectrum of **5**; Figure S30. ^1^H-NMR spectrum of **5** in C_6_D_6_; Figure S31. ^13^C-NMR spectrum of **5** in C_6_D_6_; Figure S32. HSQC spectrum of **5** in C_6_D_6_; Figure S33. ^1^H–^1^H COSY spectrum of **5** in C_6_D_6_; Figure S34. HMBC spectrum of **5** in C_6_D_6_; Figure S35. NOESY spectrum of **5** in C_6_D_6_; Figure S36. HR-ESI-MS spectrum of **6**; Figure S37. ^1^H-NMR spectrum of **6** in CDCl_3_; Figure S38. ^13^C-NMR spectrum of **6** in CDCl_3_; Figure S39. HSQC spectrum of **6** in CDCl_3_; Figure S40. ^1^H–^1^H COSY spectrum of **6** in CDCl_3_; Figure S41. HMBC spectrum of **6** in CDCl_3_; Figure S42. NOESY spectrum of **6** in CDCl_3_; Figure S43. HR-ESI-MS spectrum of **7**; Figure S44. ^1^H-NMR spectrum of **7** in CDCl_3_; Figure S45. ^13^C-NMR spectrum of **7** in CDCl_3_; Figure S46. HSQC spectrum of **7** in CDCl_3_; Figure S47. ^1^H–^1^H COSY spectrum of **7** in CDCl_3_; Figure S48. HMBC spectrum of **7** in CDCl_3_; Figure S49. NOESY spectrum of **7** in CDCl_3_.
