# Supplementary material for: Anti-Inflammatory Cembranoids from a Formosa Soft Coral Sarcophyton cherbonnieri
Source: Mar Drugs. 2020 Nov 19;18(11):573. doi: 10.3390/md18110573 (PMC7699541; doi:10.3390/md18110573)
Supplement: Supplementary file 1 [file marinedrugs-18-00573-s001.pdf]

Supporting Information

Anti-inflammatory Cembranoids from a Formosa Soft Coral *Sarcophyton cherbonnieri*

Chia-Chi Peng [1,⊥], Chiung-Yao Huang [1,⊥], Atallah F. Ahmed [2,3], Tsong-Long Hwang [4,5,6] and Jyh-Horng Sheu [1,7,8,*]

[1] Department of Marine Biotechnology and Resources, National Sun Yat-sen University, Kaohsiung 804, Taiwan; m045020008@student.nsysu.edu.tw (C.-C.P.), huangcy@mail.nsysu.edu.tw (C.-Y.H.)
[2] Department of Pharmacognosy, College of Pharmacy, King Saud University, Riyadh 11451, Saudi Arabia; afahmed@ksu.edu.sa (A.F.A.)
[3] Department of Pharmacognosy, Faculty of Pharmacy, Mansoura University, Mansoura 35516, Egypt
[4] Graduate Institute of Natural Products, College of Medicine, Chang Gung University, Taoyuan 333, Taiwan;htl@mail.cgu.edu.tw
[5] Research Center for Industry of Human Ecology and Graduate Institute of Health Industry Technology, Chang Gung University of Science and Technology, Taoyuan 333, Taiwan
[6] Department of Anesthesiology, Chang Gung Memorial Hospital, Taoyuan 333, Taiwan
[7] Graduate Institute of Natural Products, Kaohsiung Medical University, Kaohsiung 807, Taiwan
[8] Department of Medical Research, China Medical University Hospital, China Medical University, Taichung 404, Taiwan

[*] Correspondence: sheu@mail.nsysu.edu.tw; Tel.: +886-7-525-2000 (ext. 5030); Fax: +886-7-525-5020.
[⊥] These authors contributed equally to this work

Table of Contents

Figure S1. HREIMS spectrum of **1**	S4
Figure S2. ^{1}H NMR spectrum of **1** in C_6D_6	S5
Figure S3. ^{13}C NMR spectrum of **1** in C_6D_6	S6
Figure S4. HSQC spectrum of **1** in C_6D_6	S7
Figure S5. ^{1}H-^{1}H COSY spectrum of **1** in C_6D_6	S8
Figure S6. HMBC spectrum of **1** in C_6D_6	S9
Figure S7. NOESY spectrum of **1** in C_6D_6	S10
Figure S8. HREIMS spectrum of **2**	S11
Figure S9. ^{1}H NMR spectrum of **2** in C_6D_6	S12
Figure S10. ^{13}C NMR spectrum of **2** in C_6D_6	S13
Figure S11. HSQC spectrum of **2** in C_6D_6	S14
Figure S12. ^{1}H-^{1}H COSY spectrum of **2** in C_6D_6	S15
Figure S13. HMBC spectrum of **2** in C_6D_6	S16
Figure S14. NOESY spectrum of **2** in C_6D_6	S17
Figure S15. HREIMS spectrum of **3**	S18
Figure S16. ^{1}H NMR spectrum of **3** in C_6D_6	S19
Figure S17. ^{13}C NMR spectrum of **3** in C_6D_6	S20
Figure S18. HSQC spectrum of **3** in C_6D_6	S21
Figure S19. ^{1}H-^{1}H COSY spectrum of **3** in C_6D_6	S22
Figure S20. HMBC spectrum of **3** in C_6D_6	S23
Figure S21. NOESY spectrum of **3** in C_6D_6	S24
Figure S22. HREIMS spectrum of **4**	S25
Figure S23. ^{1}H NMR spectrum of **4** in C_6D_6	S26
Figure S24. ^{13}C NMR spectrum of **4** in C_6D_6	S27
Figure S25. HSQC spectrum of **4** in C_6D_6	S28
Figure S26. ^{1}H-^{1}H COSY spectrum of **4** in C_6D_6	S29
Figure S27. HMBC spectrum of **4** in C_6D_6	S30
Figure S28. NOESY spectrum of **4** in C_6D_6	S31
Figure S29. HREIMS spectrum of **5**	S32
Figure S30. ^{1}H NMR spectrum of **5** in C_6D_6	S33
Figure S31. ^{13}C NMR spectrum of **5** in C_6D_6	S34
Figure S32. HSQC spectrum of **5** in C_6D_6	S35
Figure S33. ^{1}H-^{1}H COSY spectrum of **5** in C_6D_6	S36
Figure S34. HMBC spectrum of **5** in C_6D_6	S37
Figure S35. NOESY spectrum of **5** in C_6D_6	S38

Figure S36. HREIMS spectrum of **6**	S39
Figure S37. ^{1}H NMR spectrum of **6** in $CDCl_3$	S40
Figure S38. ^{13}C NMR spectrum of **6** in $CDCl_3$	S41
Figure S39. HSQC spectrum of **6** in $CDCl_3$	S42
Figure S40. ^{1}H-^{1}H COSY spectrum of **6** in $CDCl_3$	S43
Figure S41. HMBC spectrum of **6** in $CDCl_3$	S44
Figure S42. NOESY spectrum of **6** in $CDCl_3$	S45
Figure S43. HREIMS spectrum of **7**	S46
Figure S44. ^{1}H NMR spectrum of **7** in $CDCl_3$	S47
Figure S45. ^{13}C NMR spectrum of **7** in $CDCl_3$	S48
Figure S46. HSQC spectrum of **7** in $CDCl_3$	S49
Figure S47. ^{1}H-^{1}H COSY spectrum of **7** in $CDCl_3$	S50
Figure S48. HMBC spectrum of **7** in $CDCl_3$	S51
Figure S49. NOESY spectrum of **7** in $CDCl_3$	S52

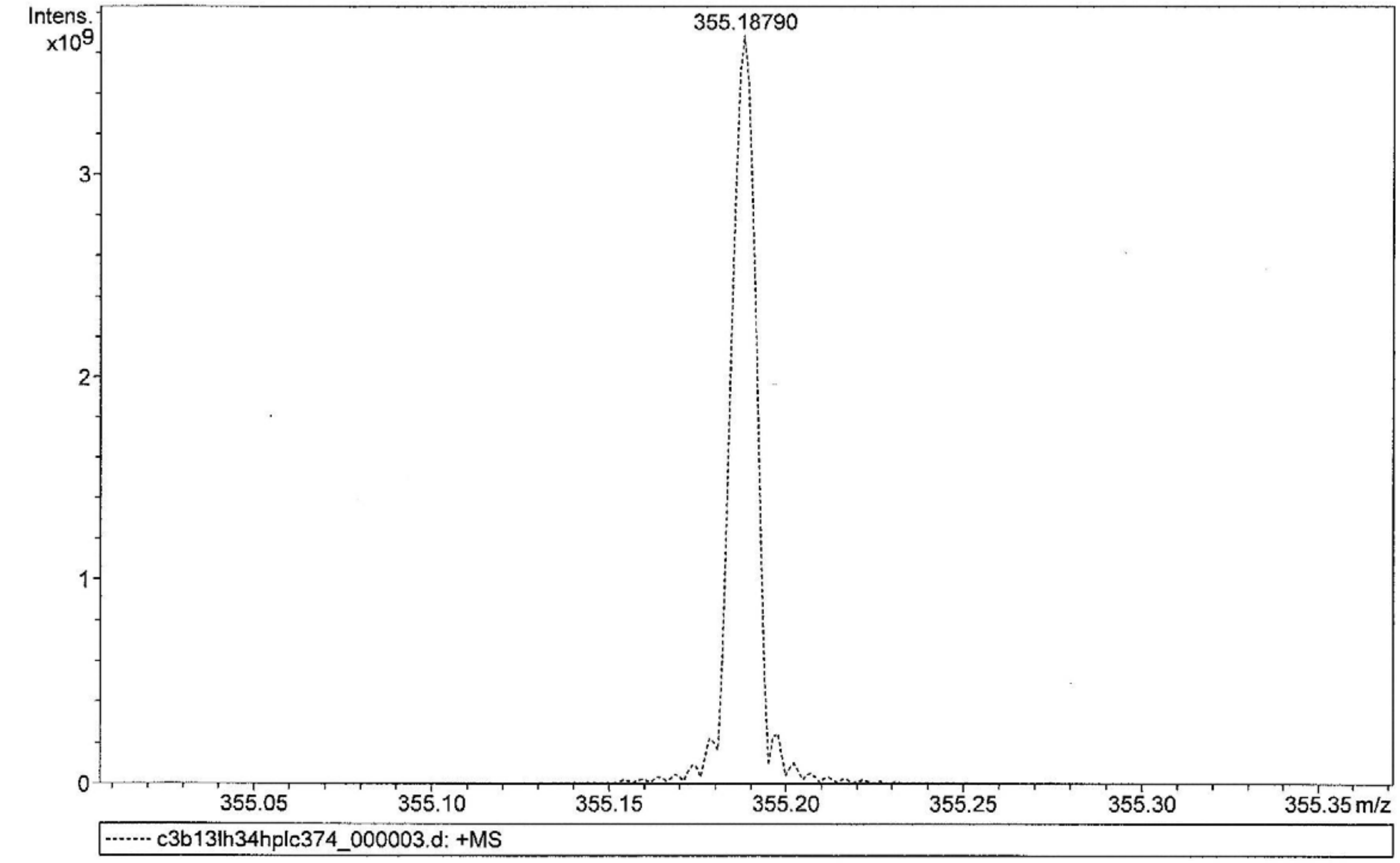

Meas. m/z	#	Formula	Score	m/z	err [mDa]	err [ppm]	mSigma	rdb	e^- Conf	N-Rule
355.18790	1	C 20 H 28 Na O 4	100.00	355.18798	0.08	0.23	4.6	6.5	even	ok

Figure S1. HRESIMS spectrum of **1**

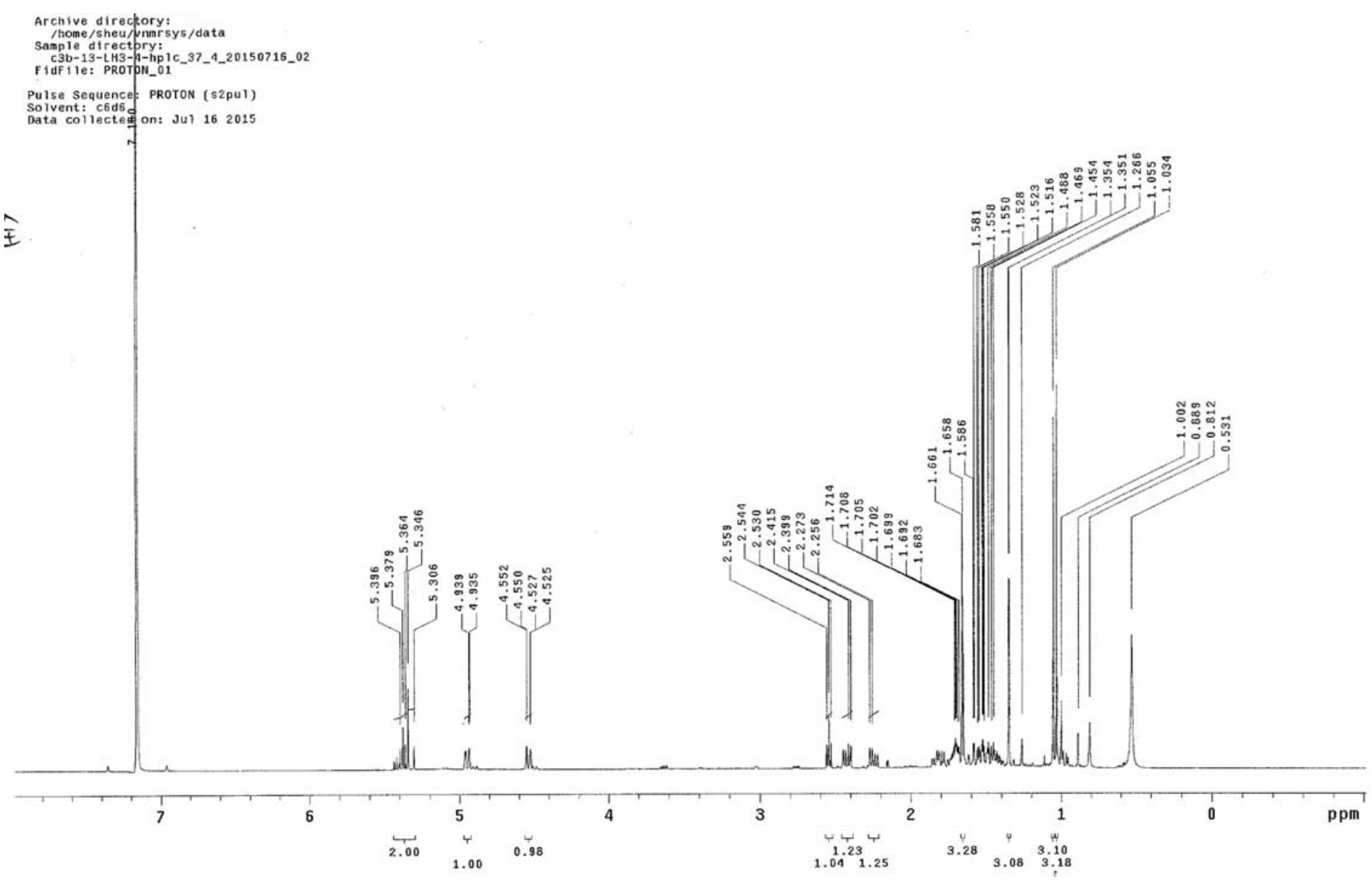

Figure S2. ^{1}H NMR spectrum of **1** in C_6D_6

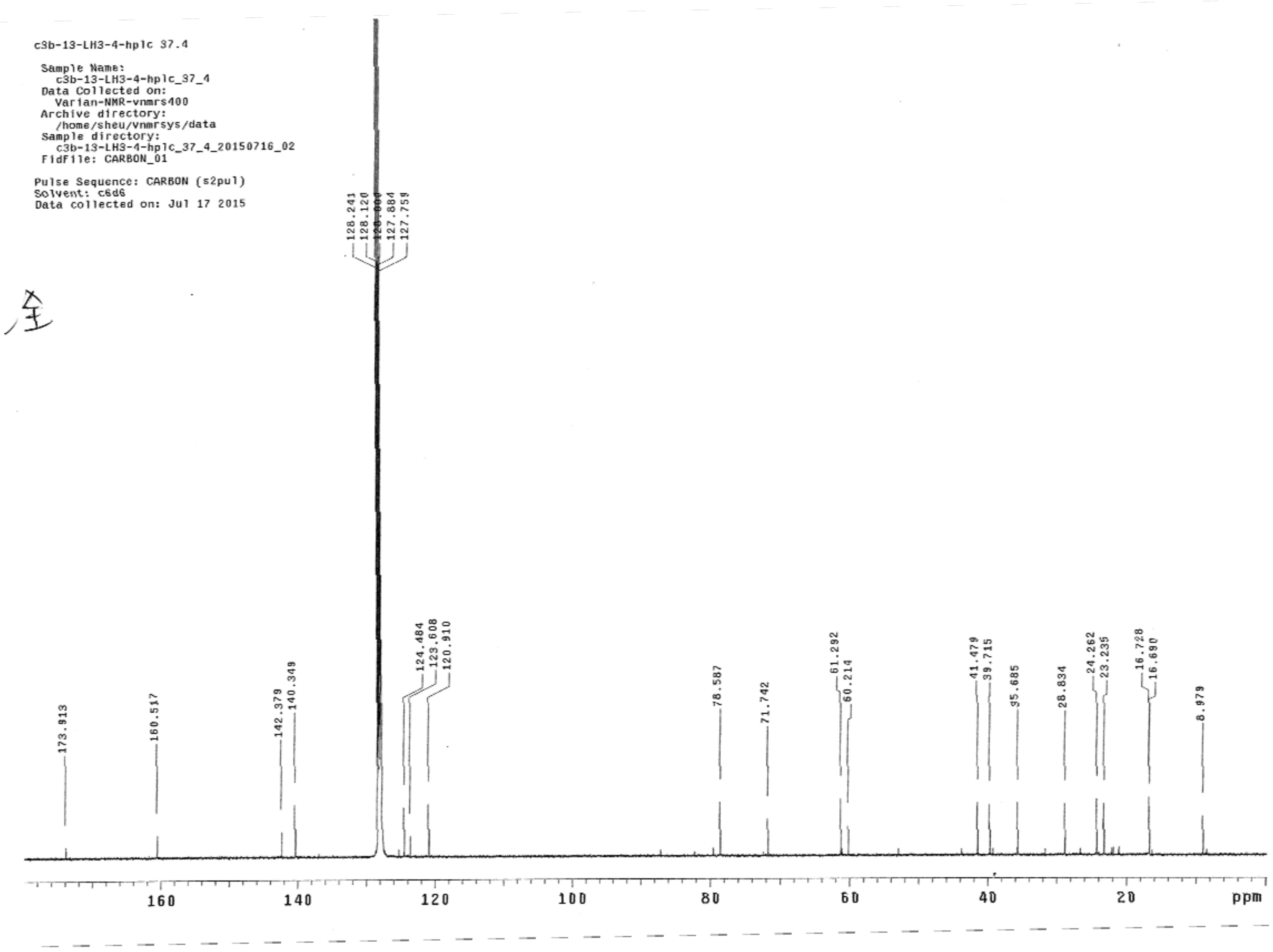

Figure S3. ^{13}C NMR spectrum of **1** in C_6D_6

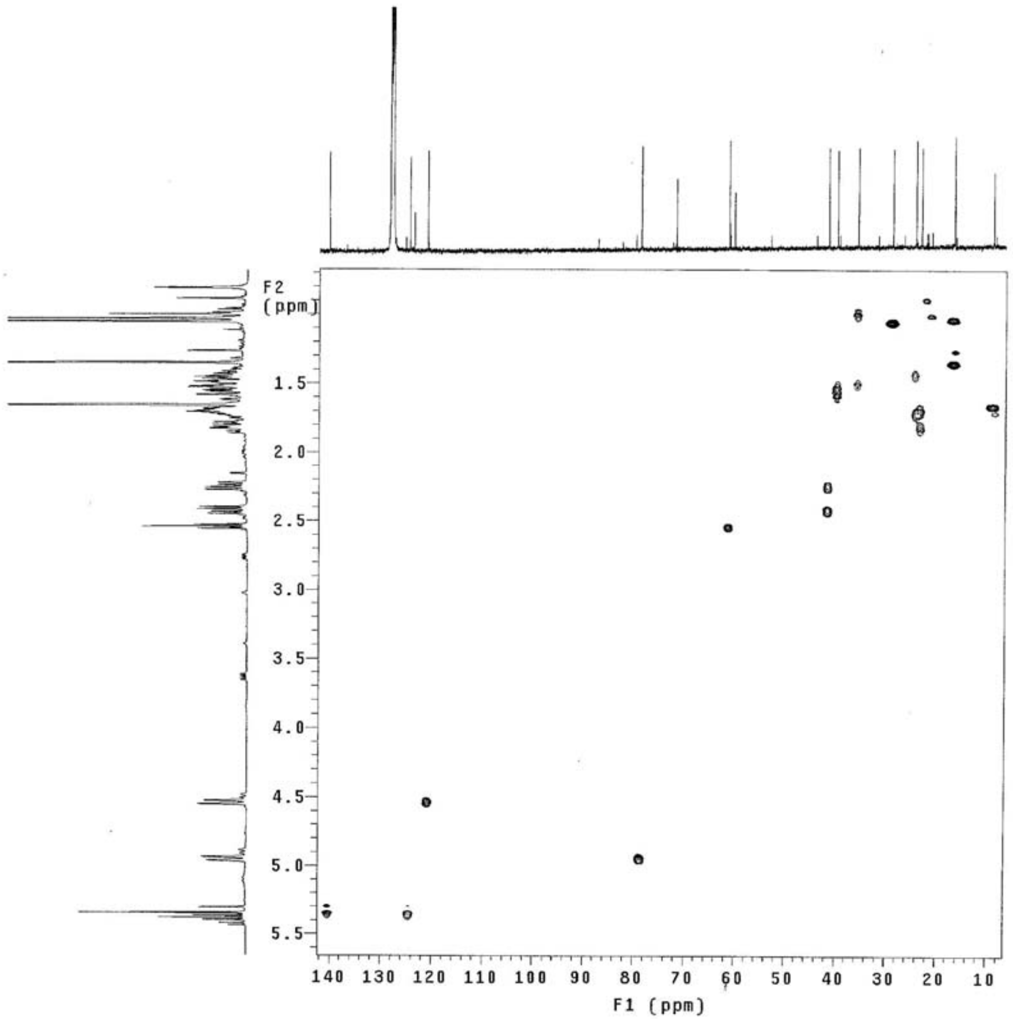

Figure S4. HSQC spectrum of **1** in C_6D_6

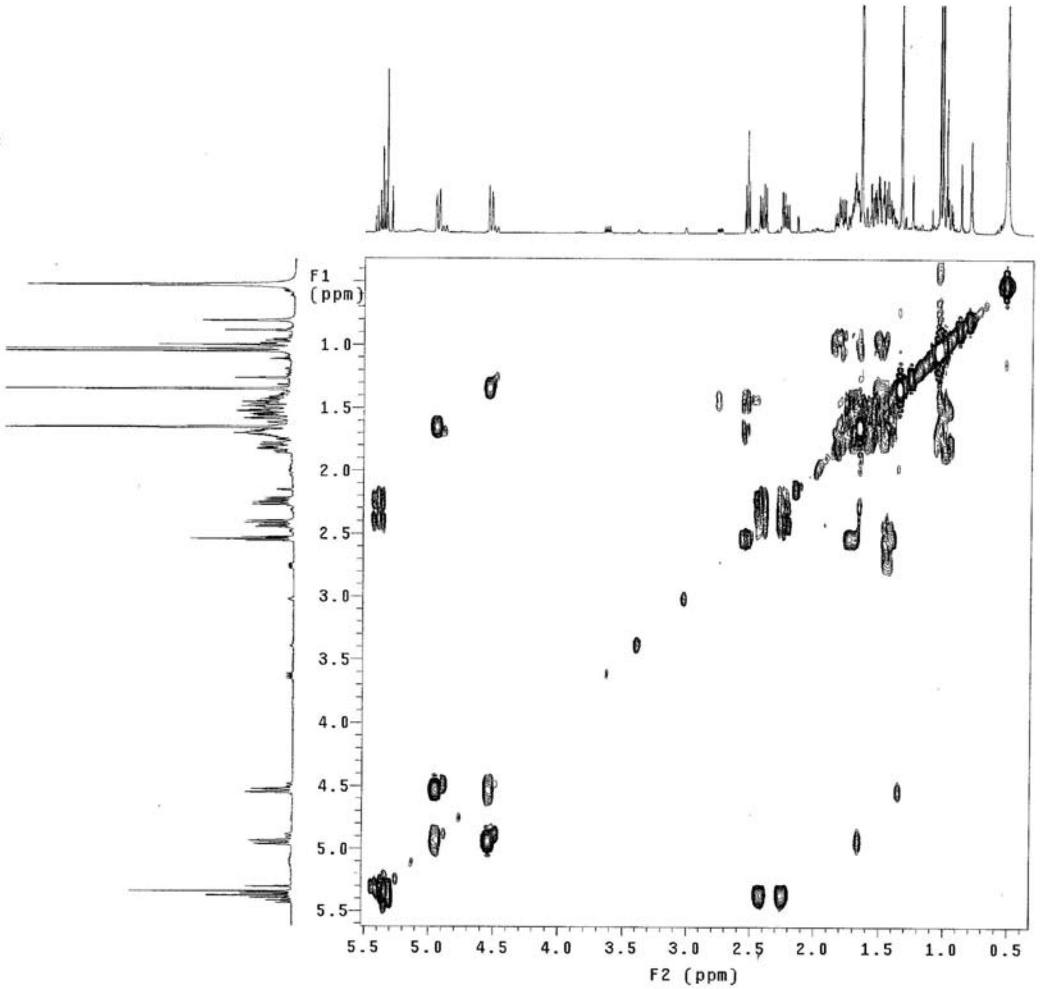

Figure S5. ^1H-^1H COSY spectrum of **1** in C_6D_6

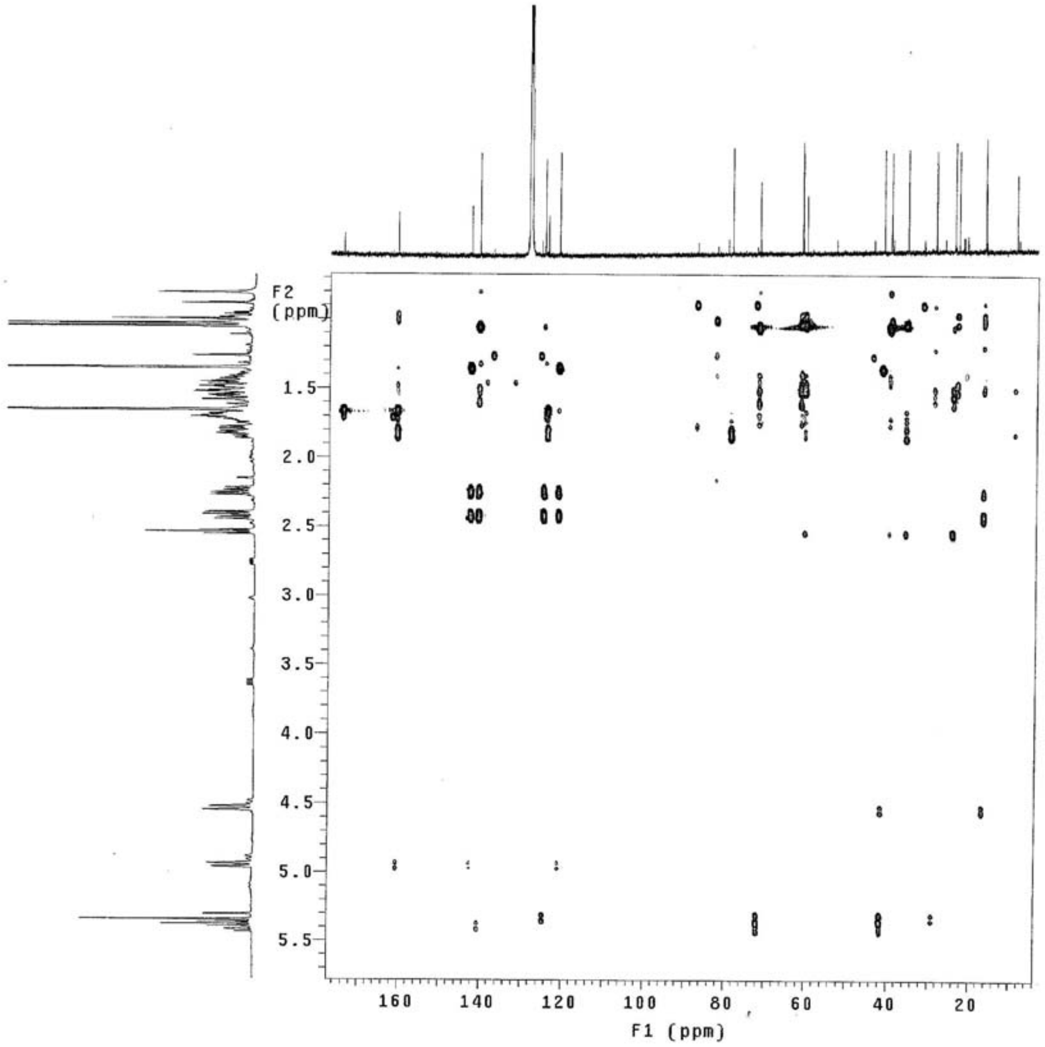

Figure S6. HMBC spectrum of **1** in C_6D_6

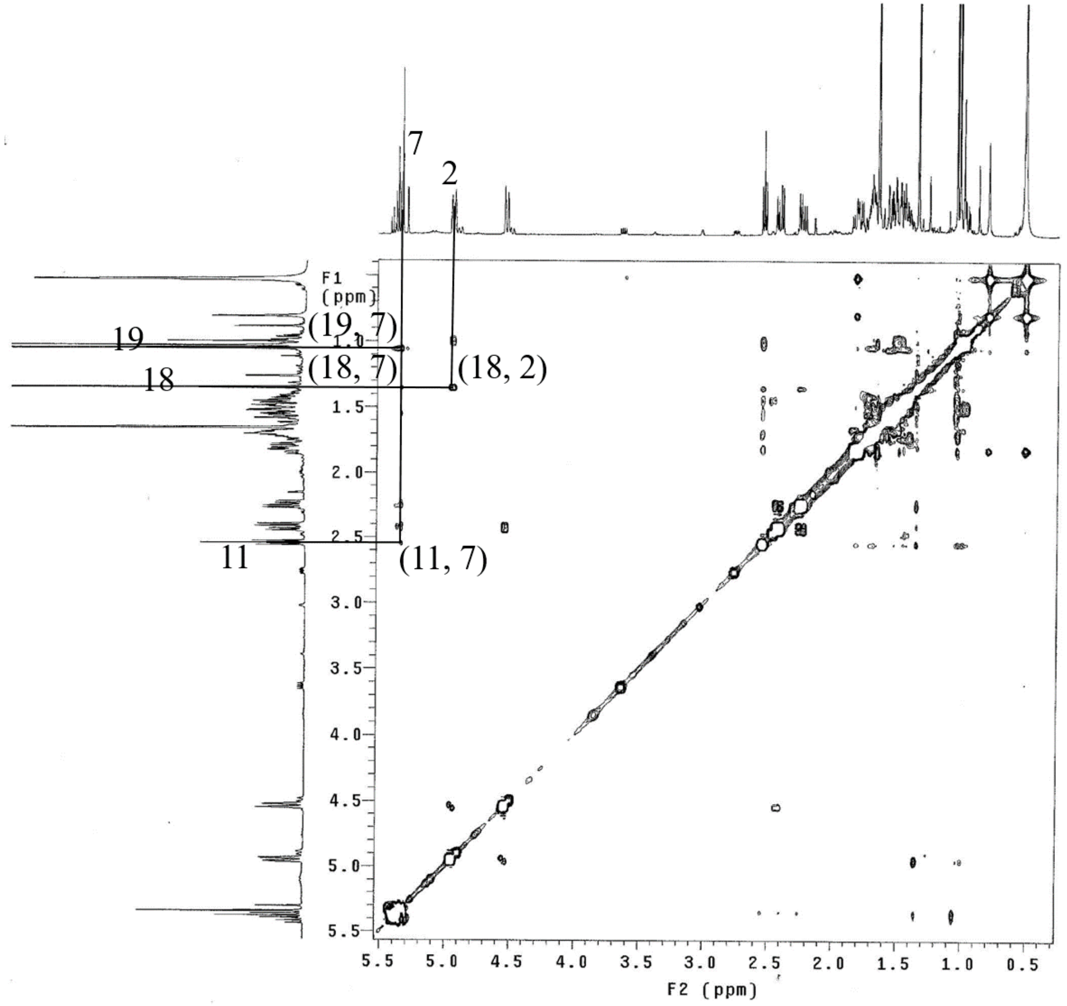

Figure S7. NOESY spectrum of **1** in C_6D_6

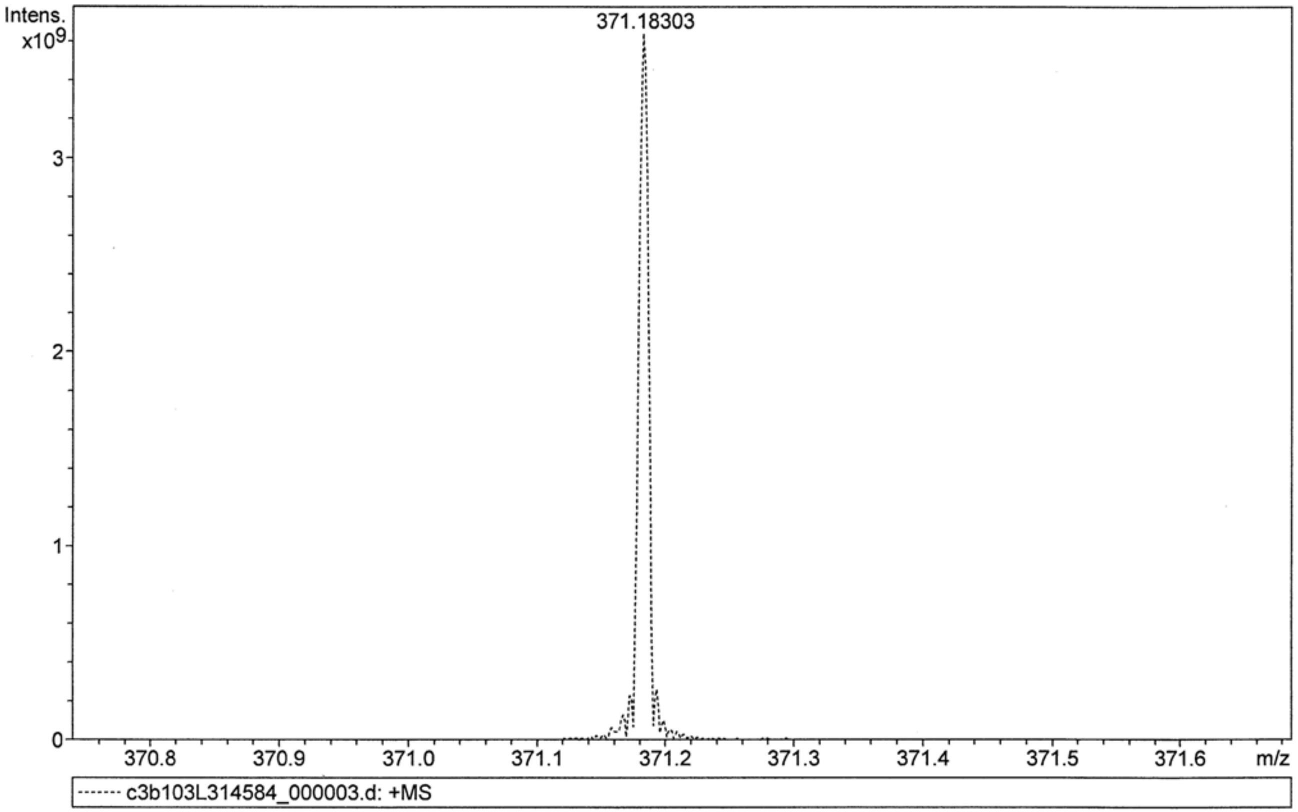

Meas. m/z	#	Formula	Score	m/z	err [mDa]	err [ppm]	mSigma	rdb	e⁻ Conf	N-Rule
371.18303	1	C 20 H 28 Na O 5	100.00	371.18290	-0.14	-0.37	6.9	6.5	even	ok

Figure S8. HRESIMS spectrum of **2**

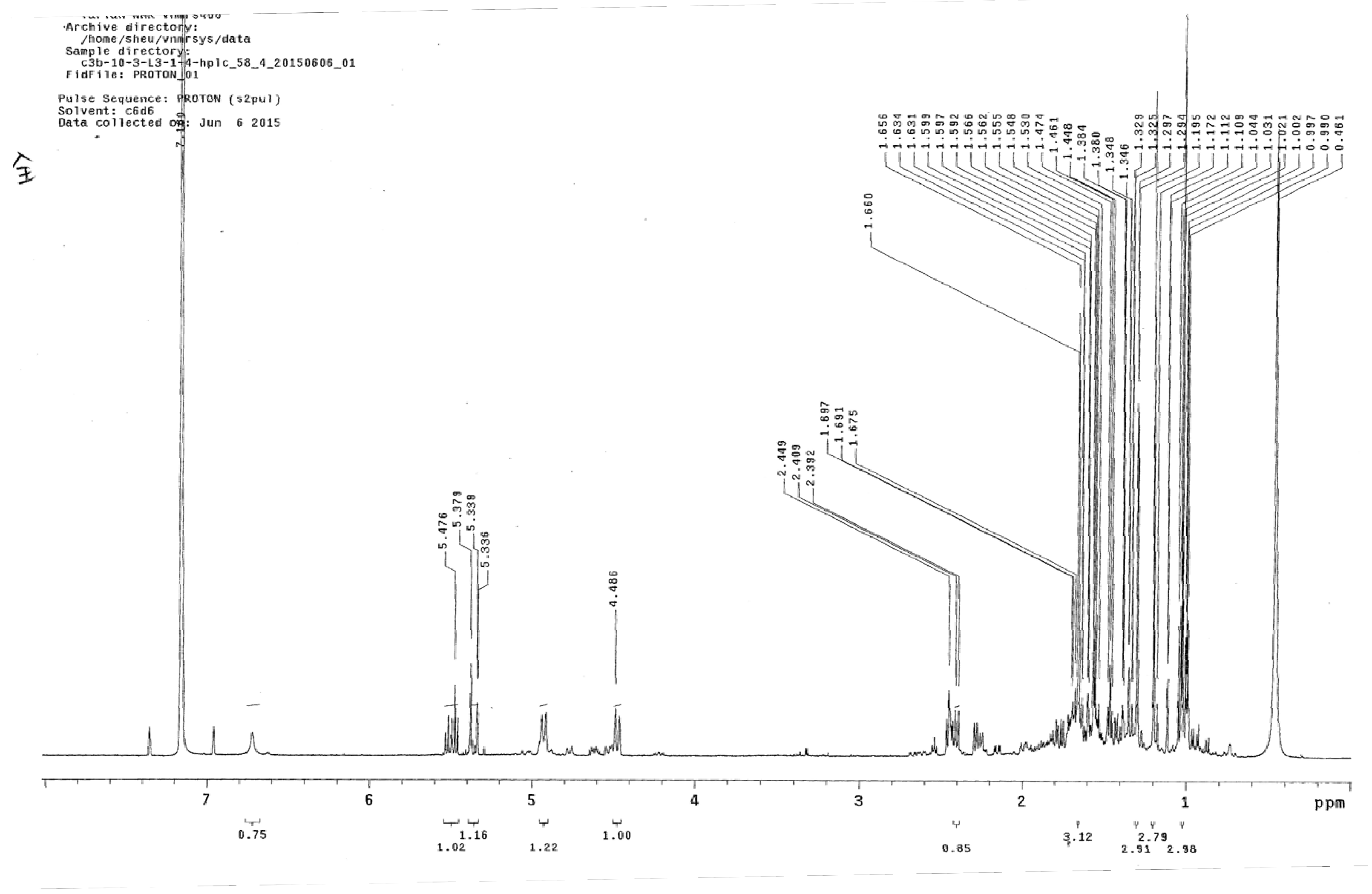

Figure S9. ^{1}H NMR spectrum of **2** in C_6D_6

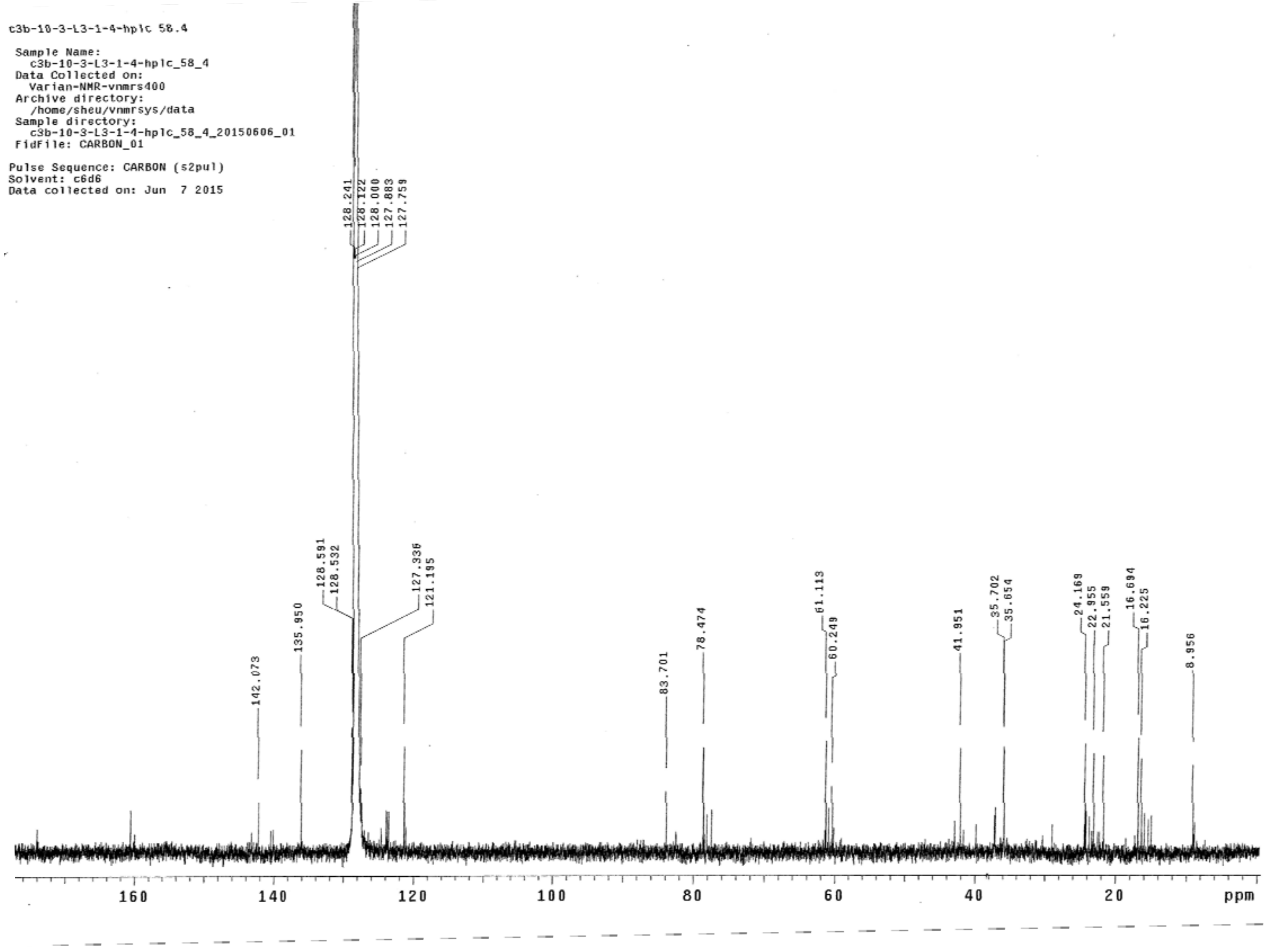

Figure S10. ^{13}C NMR spectrum of **2** in C_6D_6

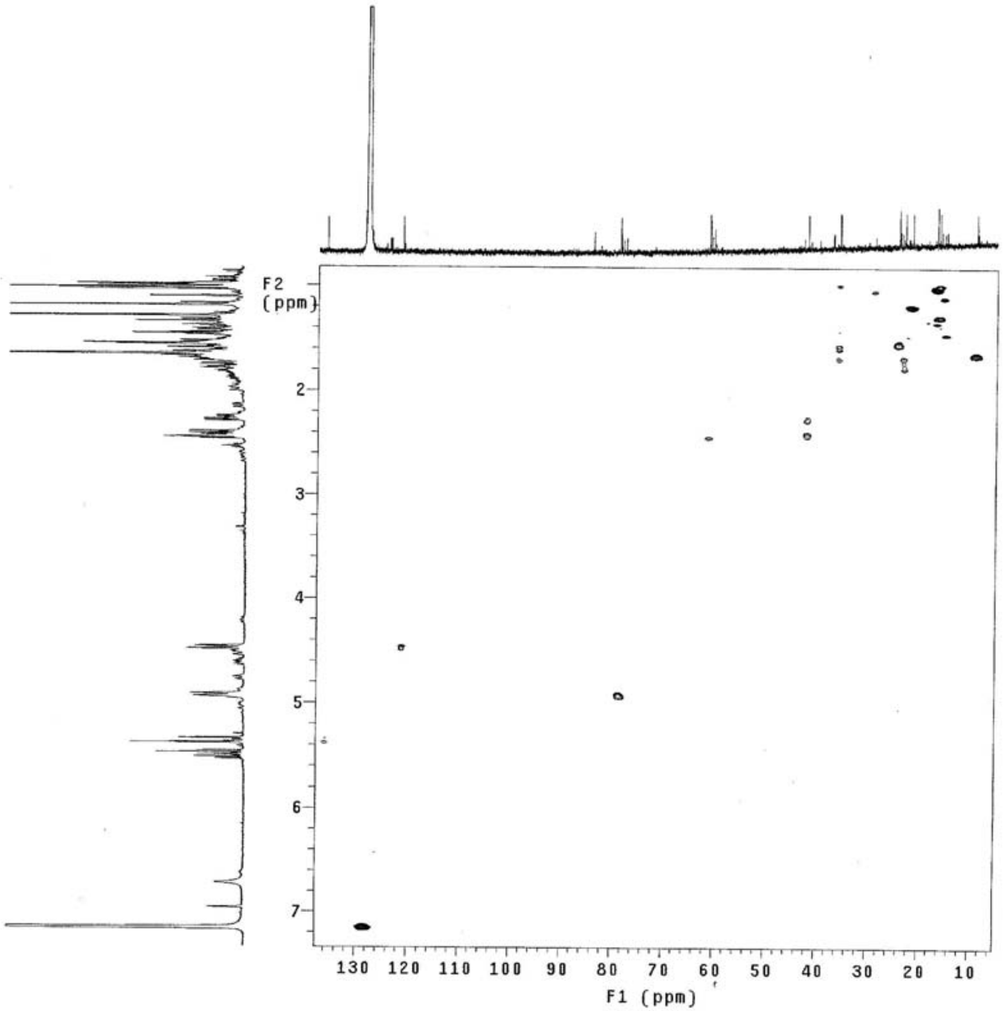

Figure S11. HSQC spectrum of **2** in C_6D_6

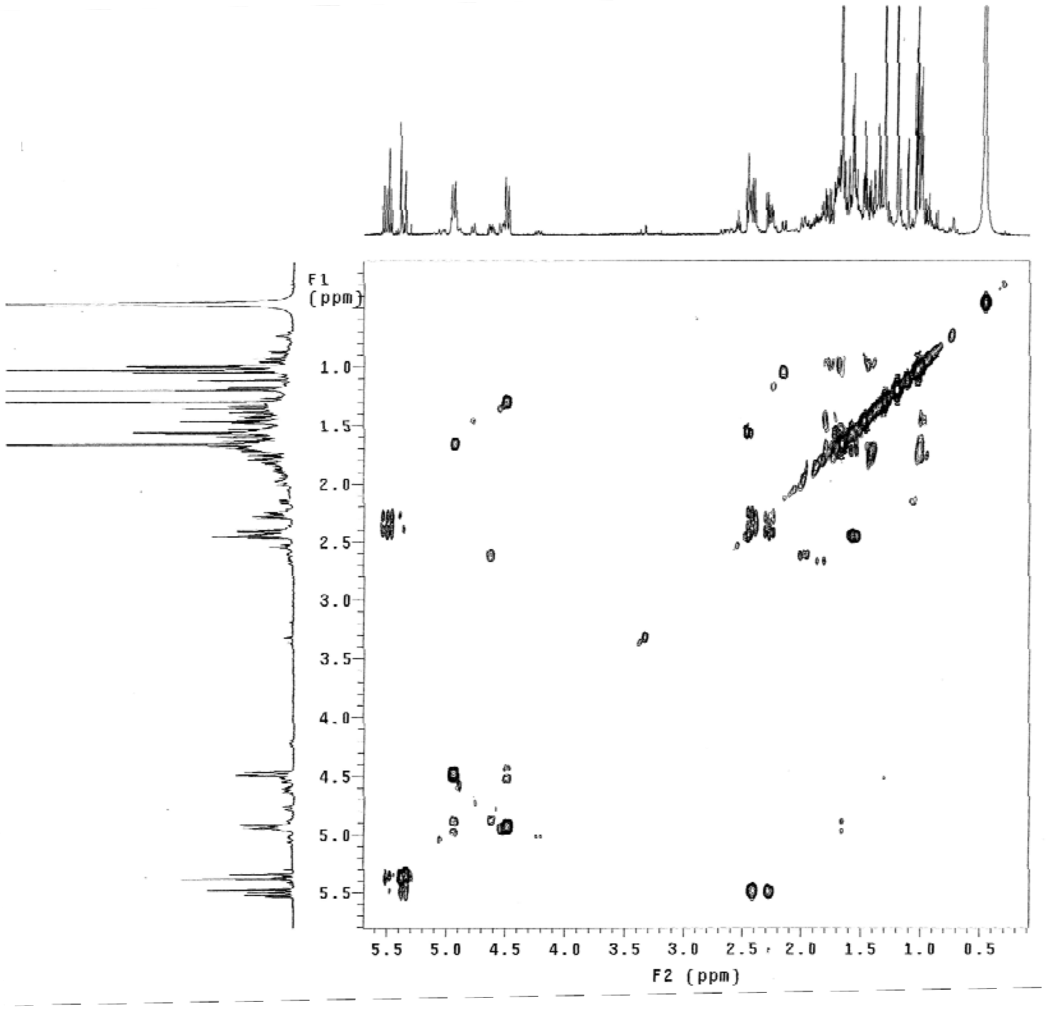

Figure S12. ^1H-^1H COSY spectrum of **2** in C_6D_6

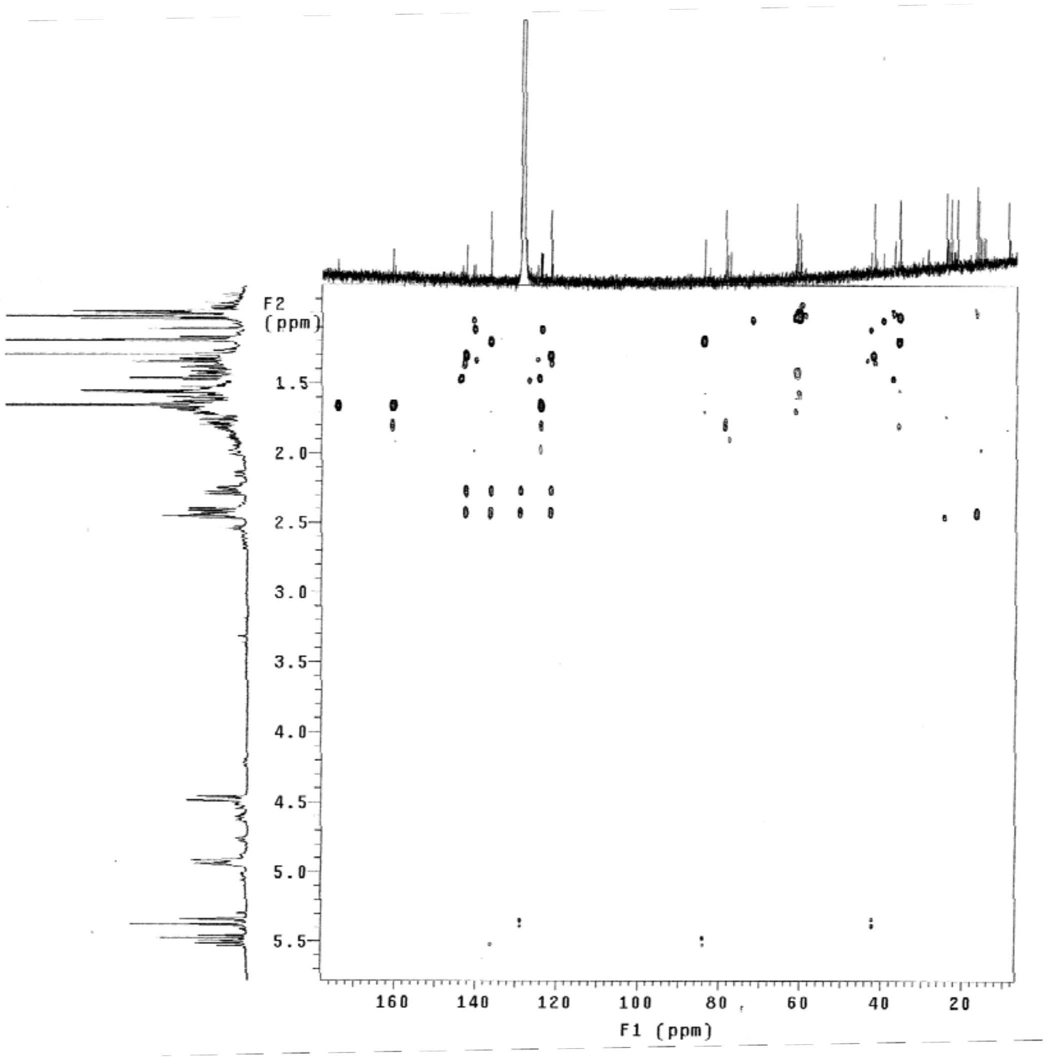

Figure S13. HMBC spectrum of **2** in C_6D_6

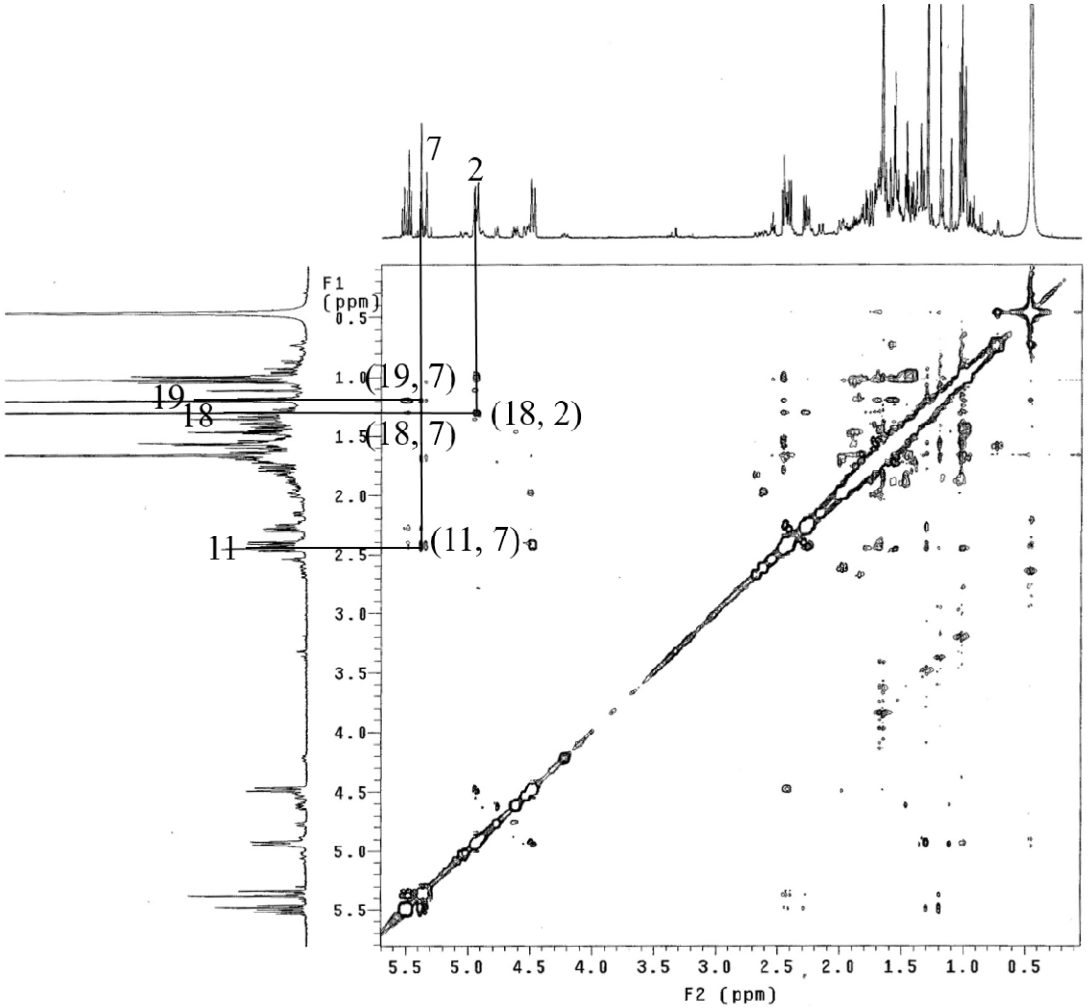

Figure S14. NOESY spectrum of **2** in C_6D_6

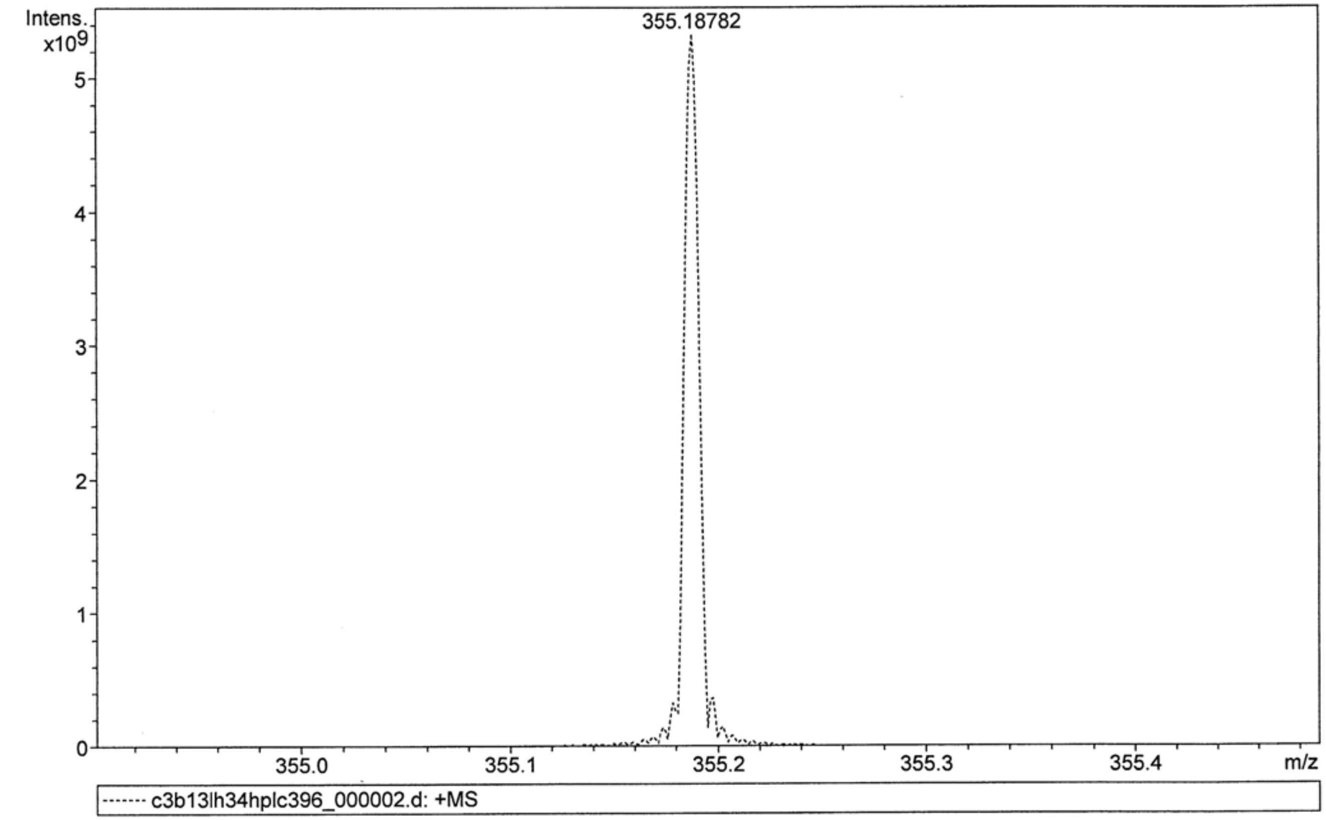

Meas. m/z	#	Formula	Score	m/z	err [mDa]	err [ppm]	mSigma	rdb	e⁻ Conf	N-Rule
355.18782	1	C 20 H 28 Na O 4	100.00	355.18798	0.16	0.45	14.0	6.5	even	ok

Figure S15. HRESIMS spectrum of **3**

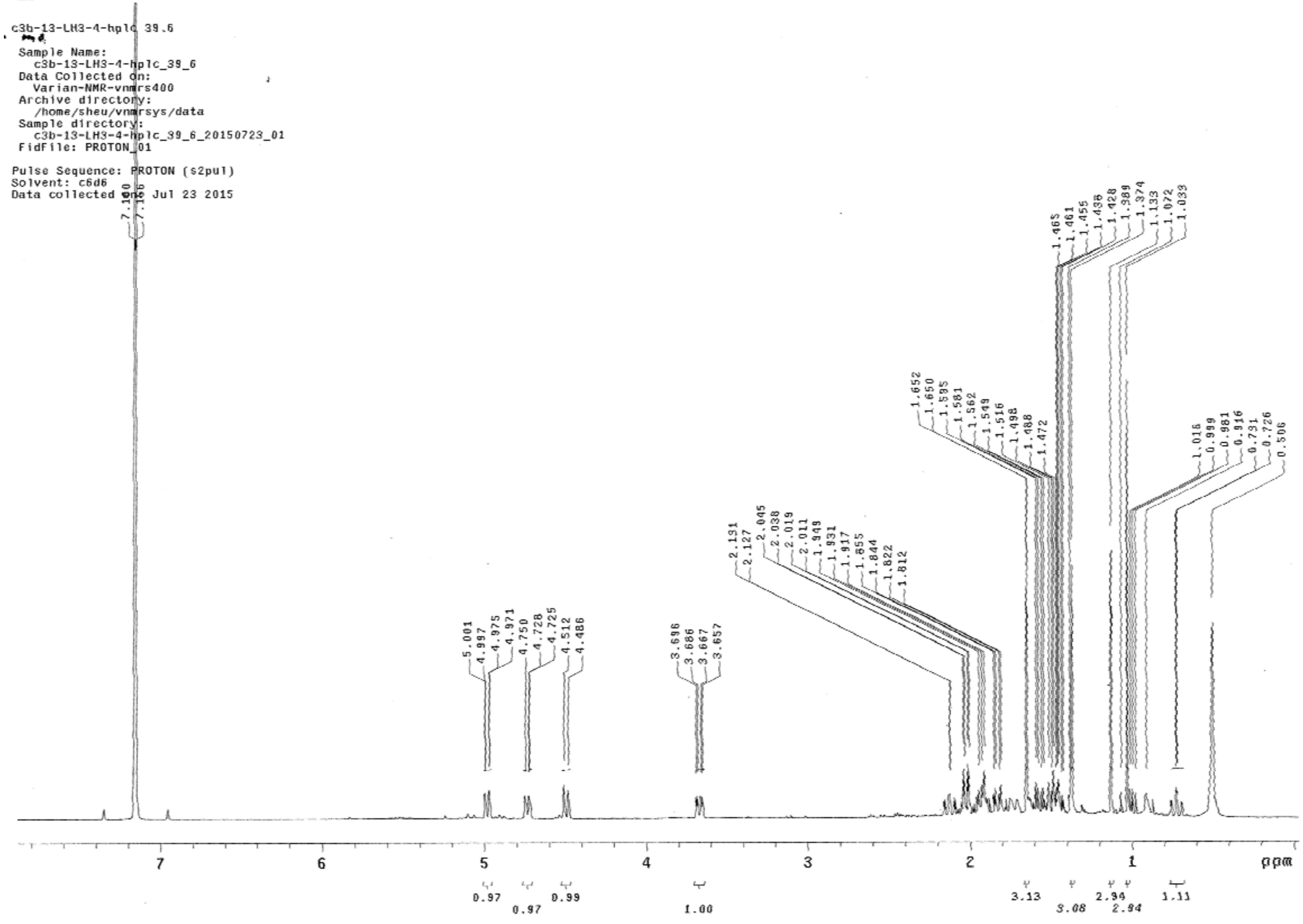

Figure S16. ^{1}H NMR spectrum of **3** in C_6D_6

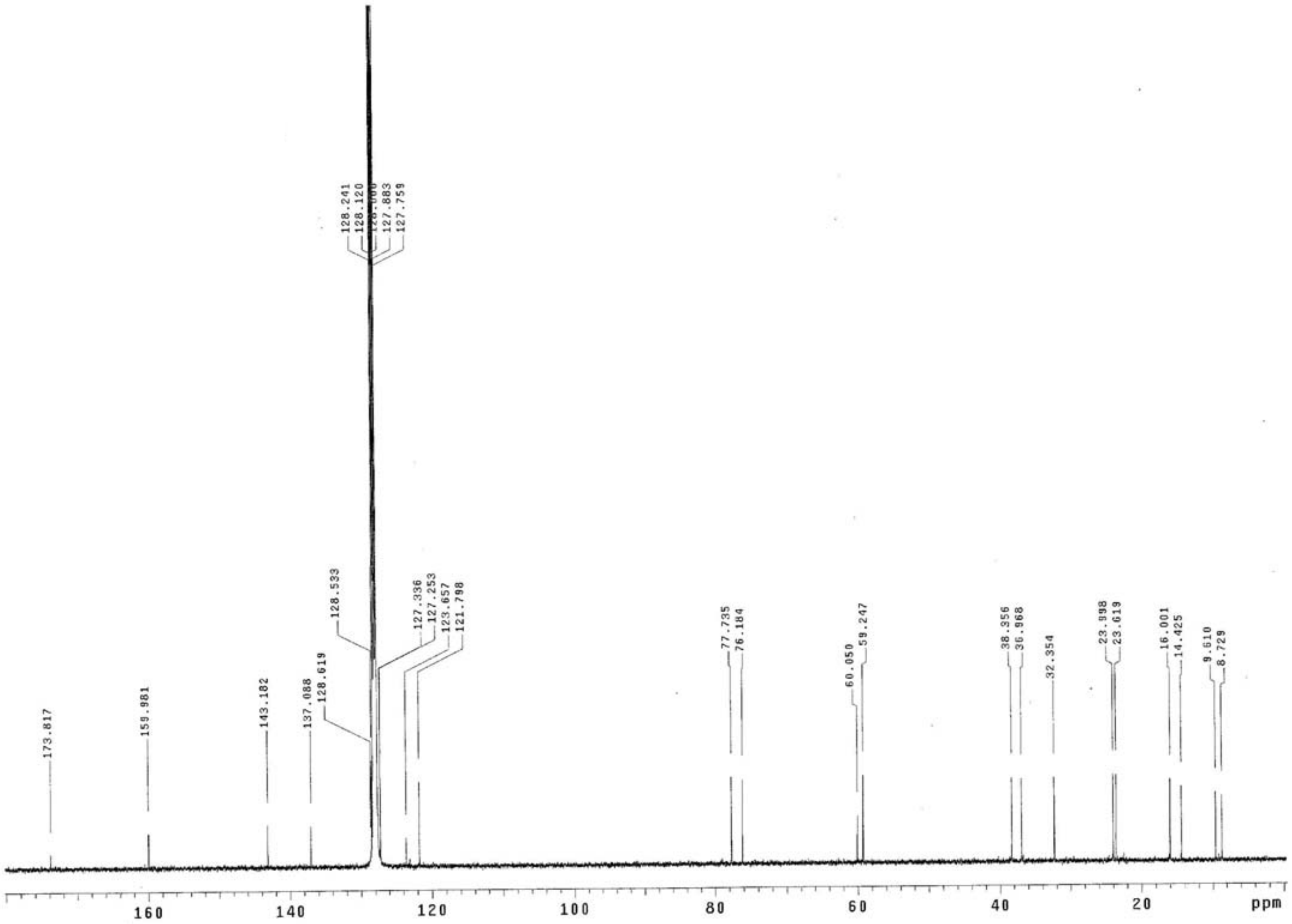

Figure S17. ^{13}C NMR spectrum of **3** in C_6D_6

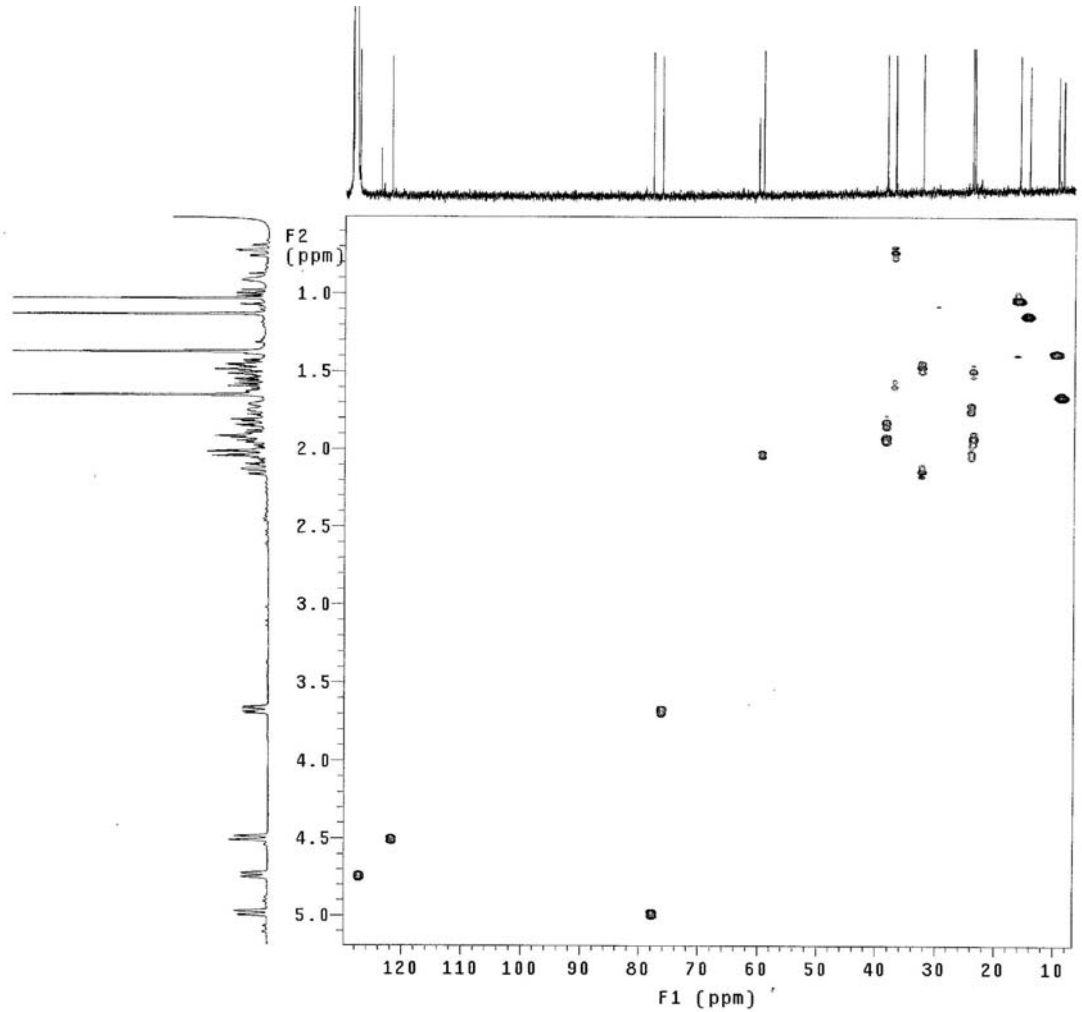

Figure S18. HSQC spectrum of **3** in C_6D_6

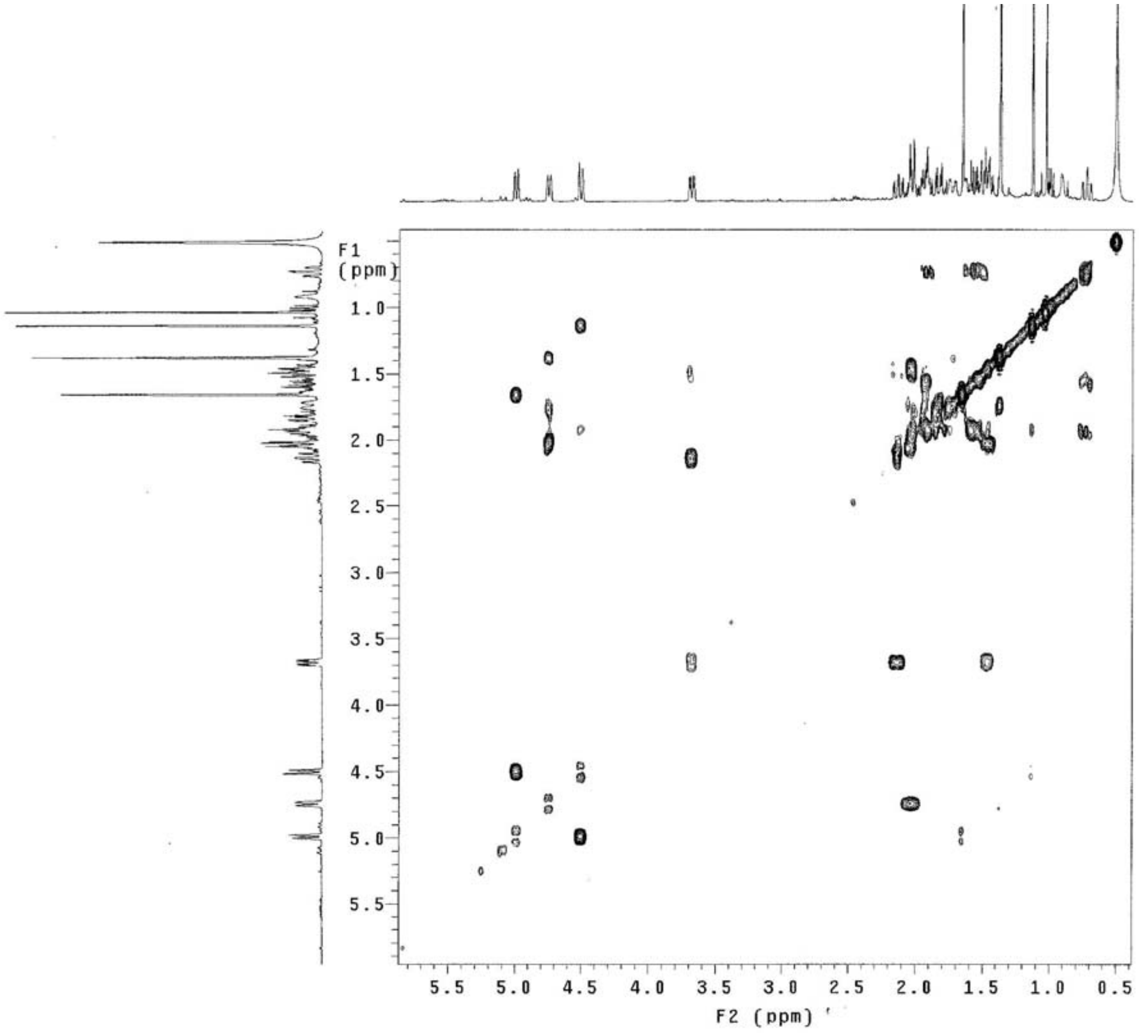

Figure S19. ^1H-^1H COSY spectrum of **3** in C_6D_6

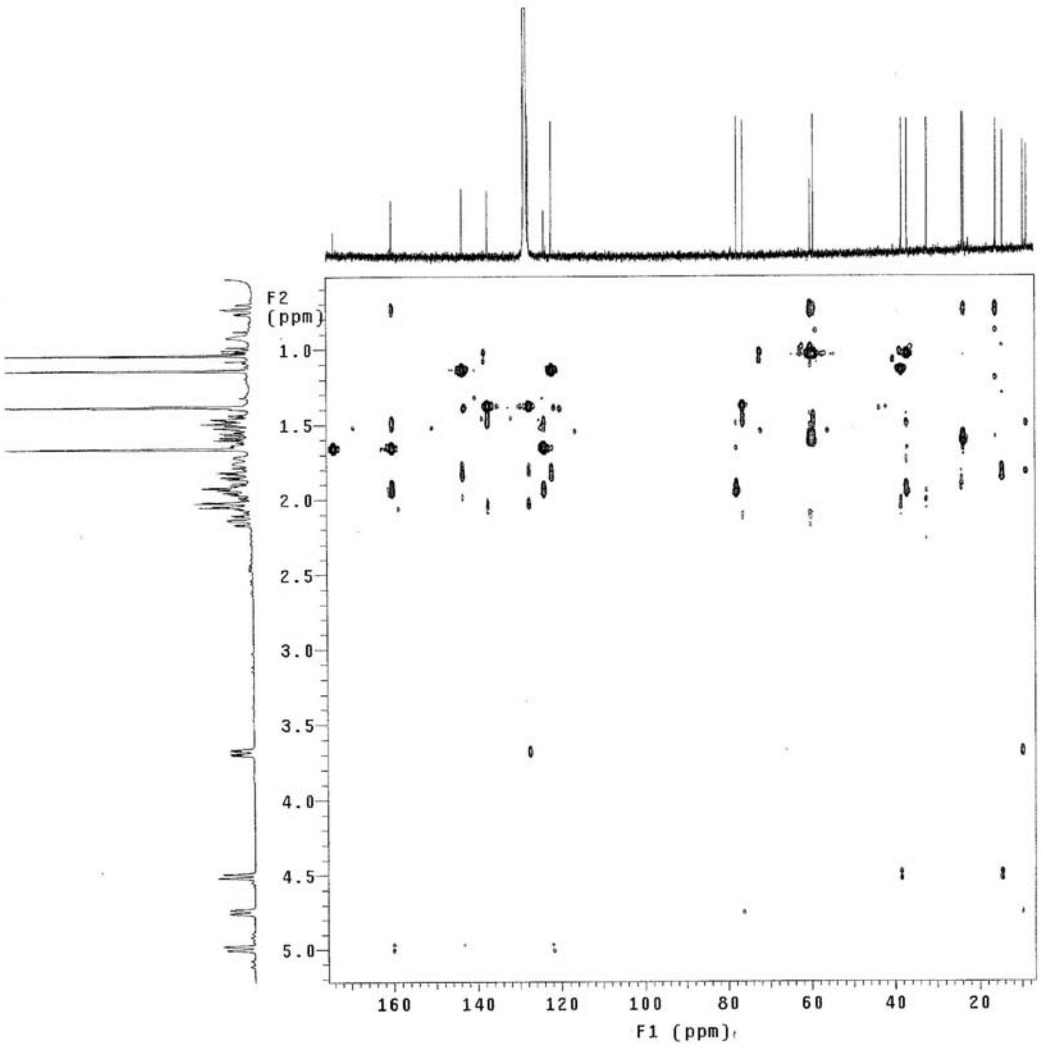

Figure S20. HMBC spectrum of **3** in C_6D_6

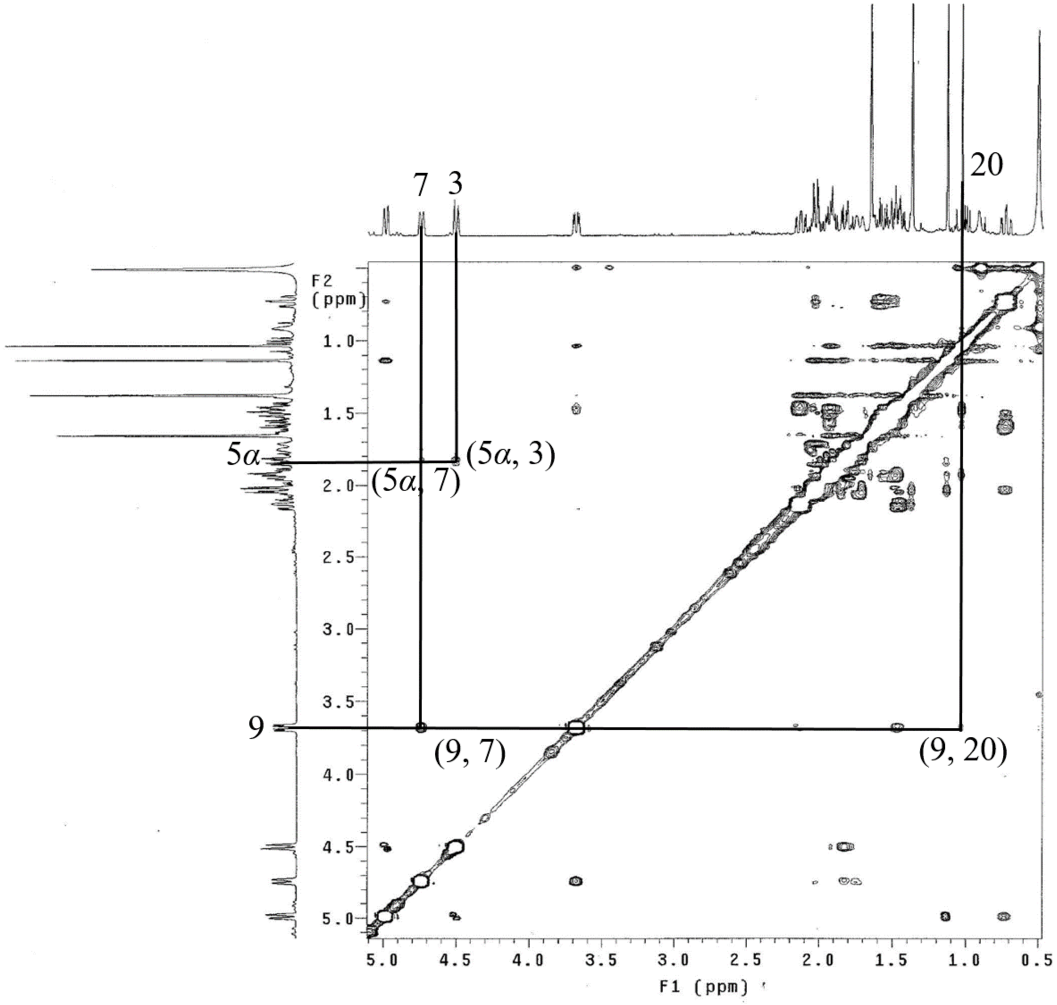

Figure S21. NOESY spectrum of **3** in C_6D_6

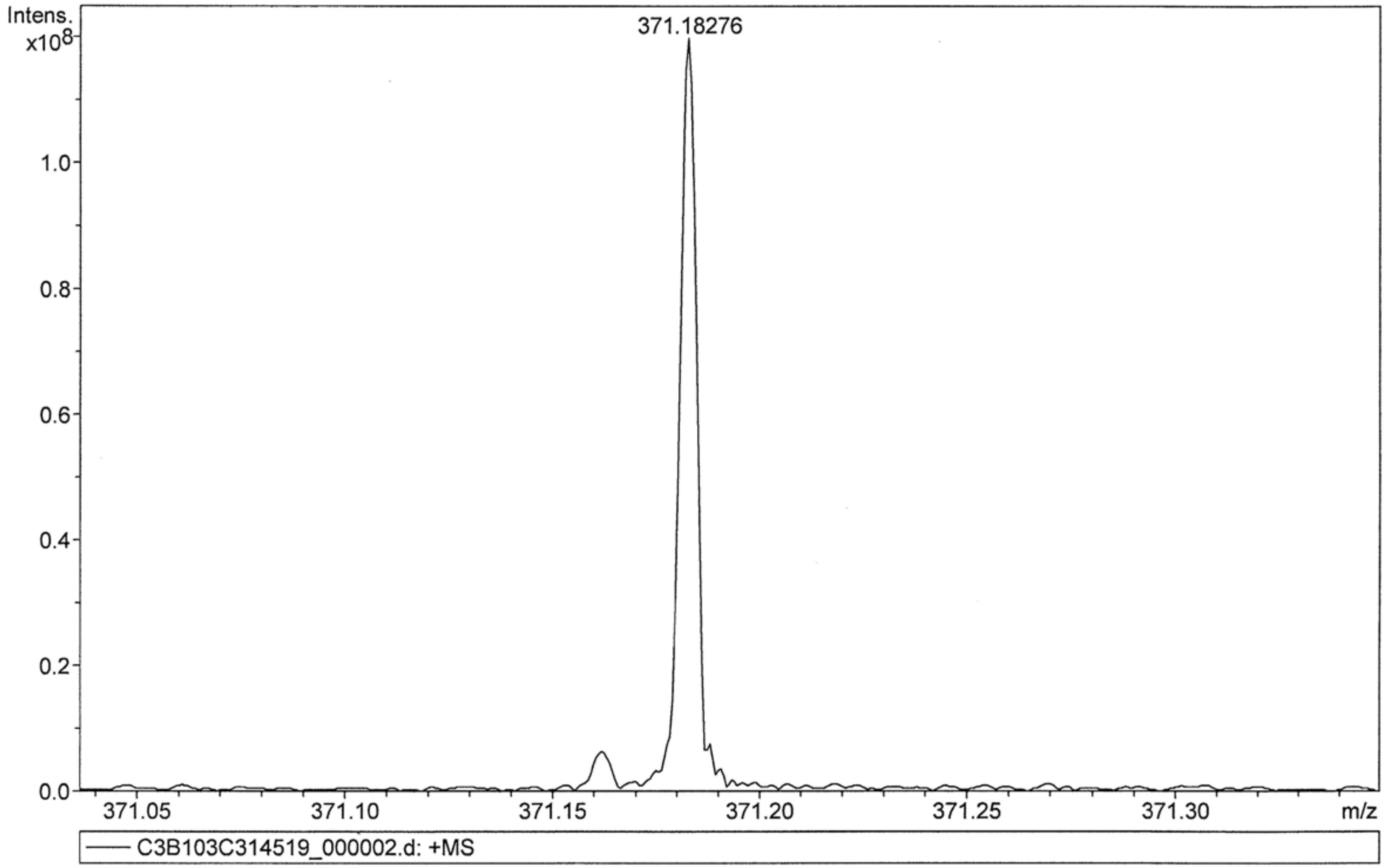

Figure S22. HREIMS spectrum of **4**

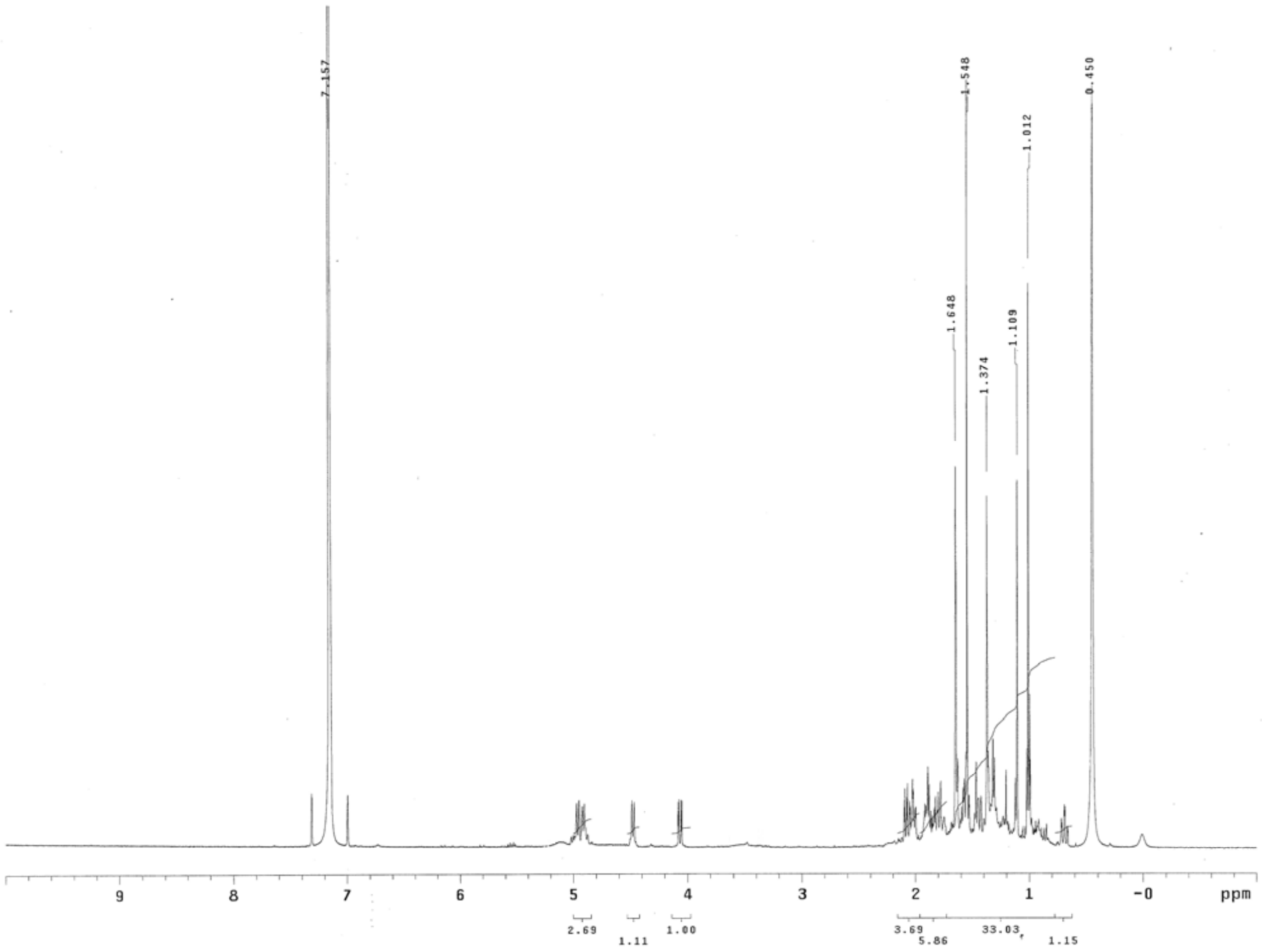

Figure S23. ^{1}H NMR spectrum of **4** in C_6D_6

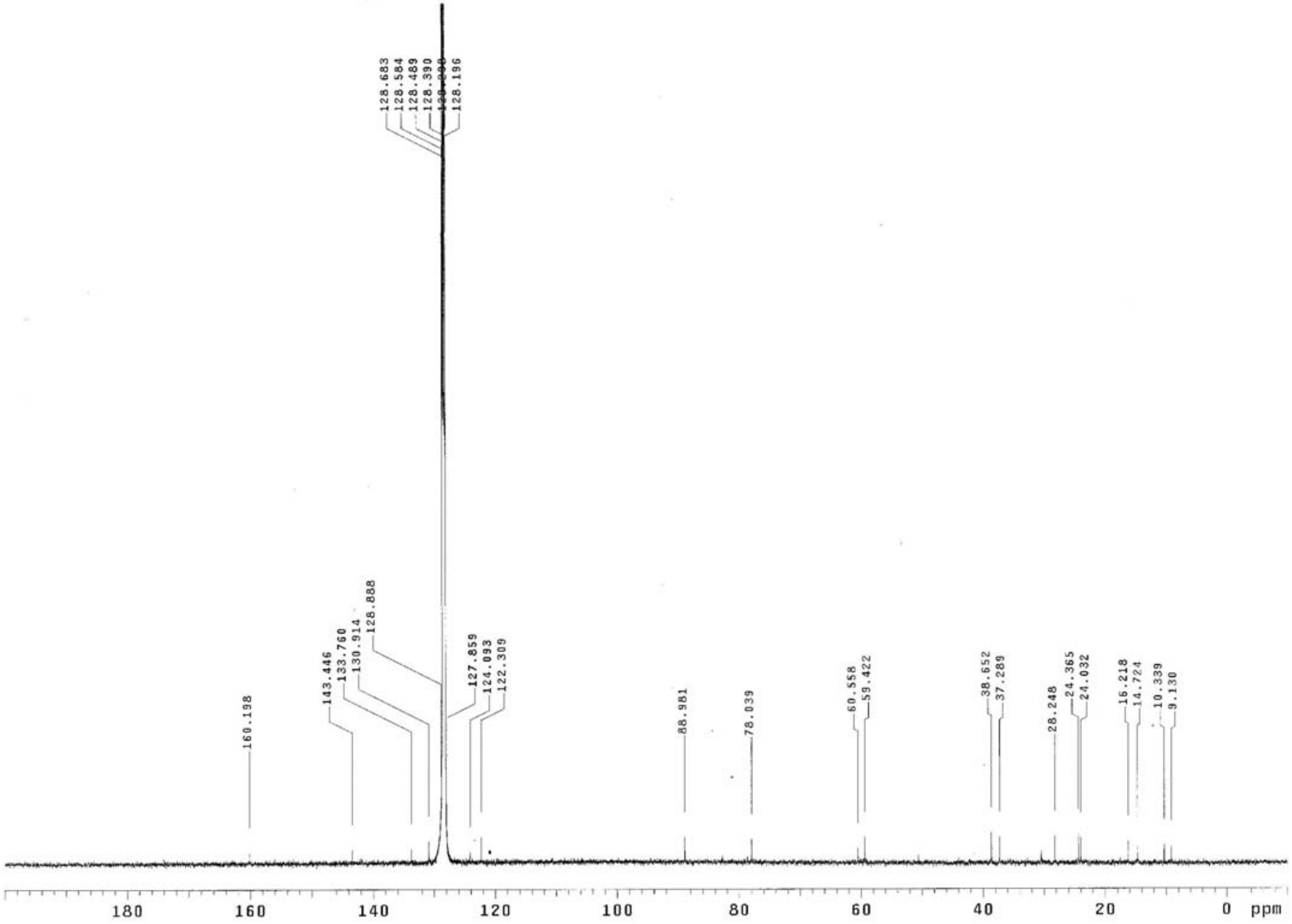

Figure S24. ^{13}C NMR spectrum of **4** in C_6D_6

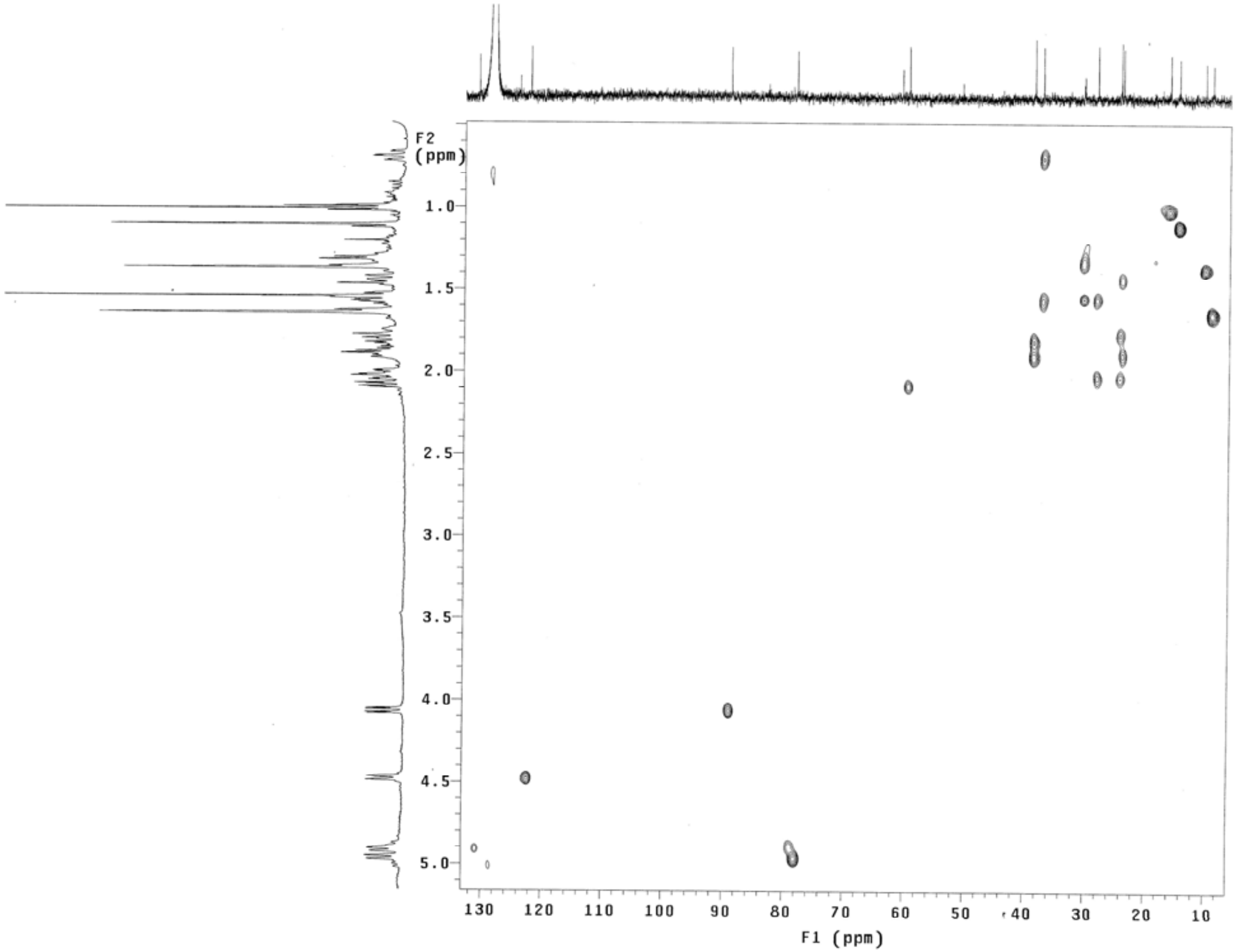

Figure S25. HSQC spectrum of **4** in C_6D_6

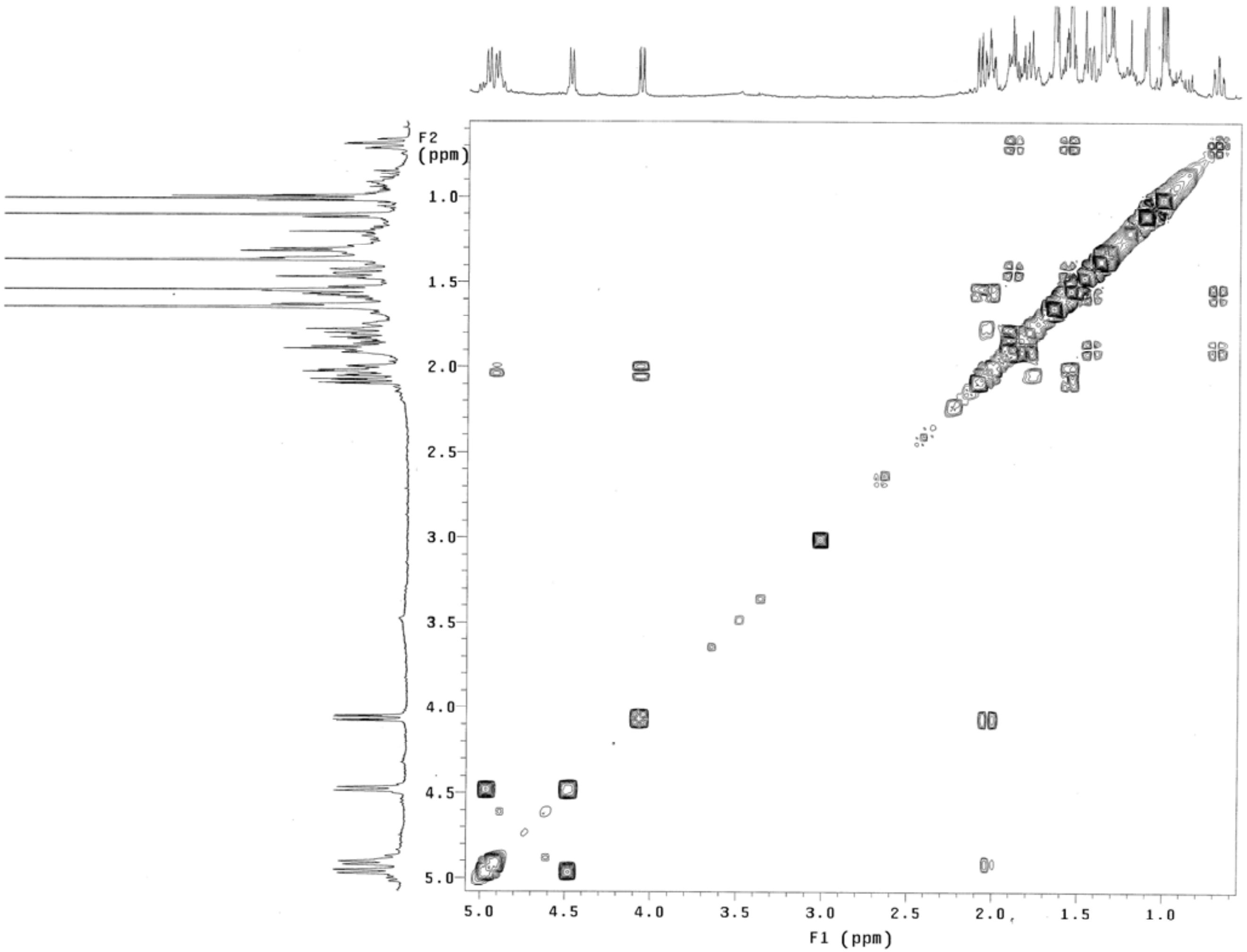

Figure S26. ^{1}H-^{1}H COSY spectrum of **4** in C_6D_6

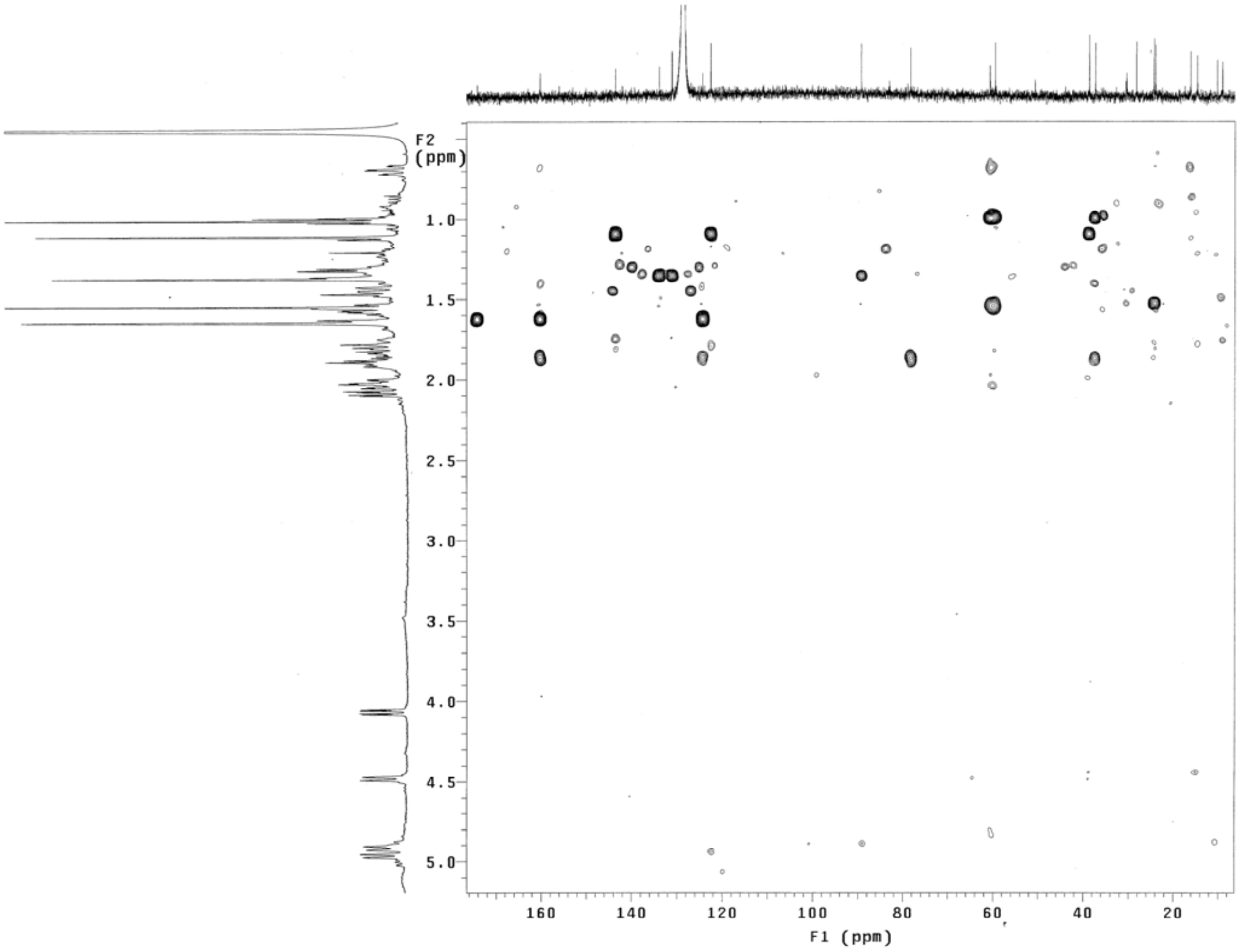

Figure S27. HMBC spectrum of **4** in C_6D_6

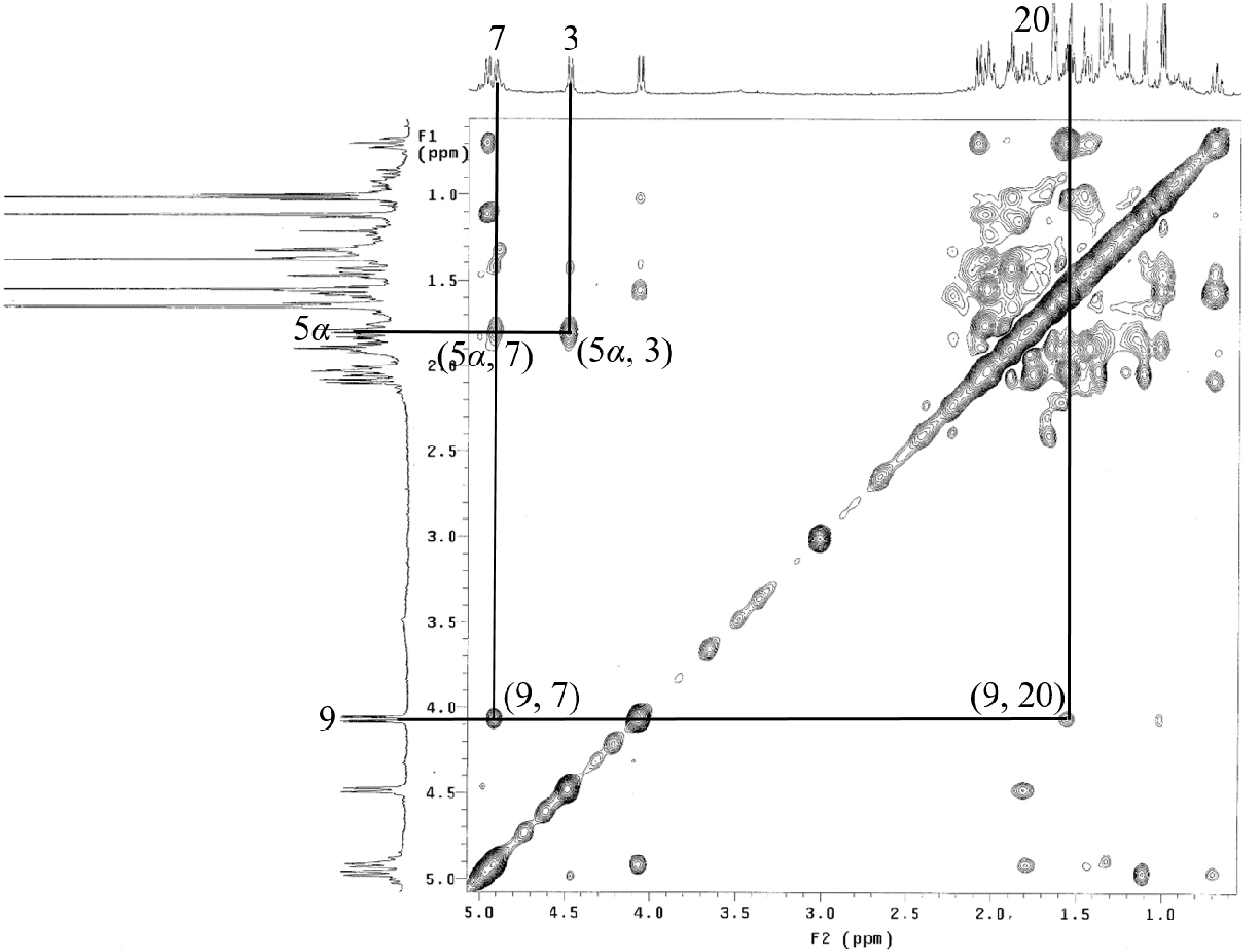

Figure S28. NOESY spectrum of **4** in C_6D_6

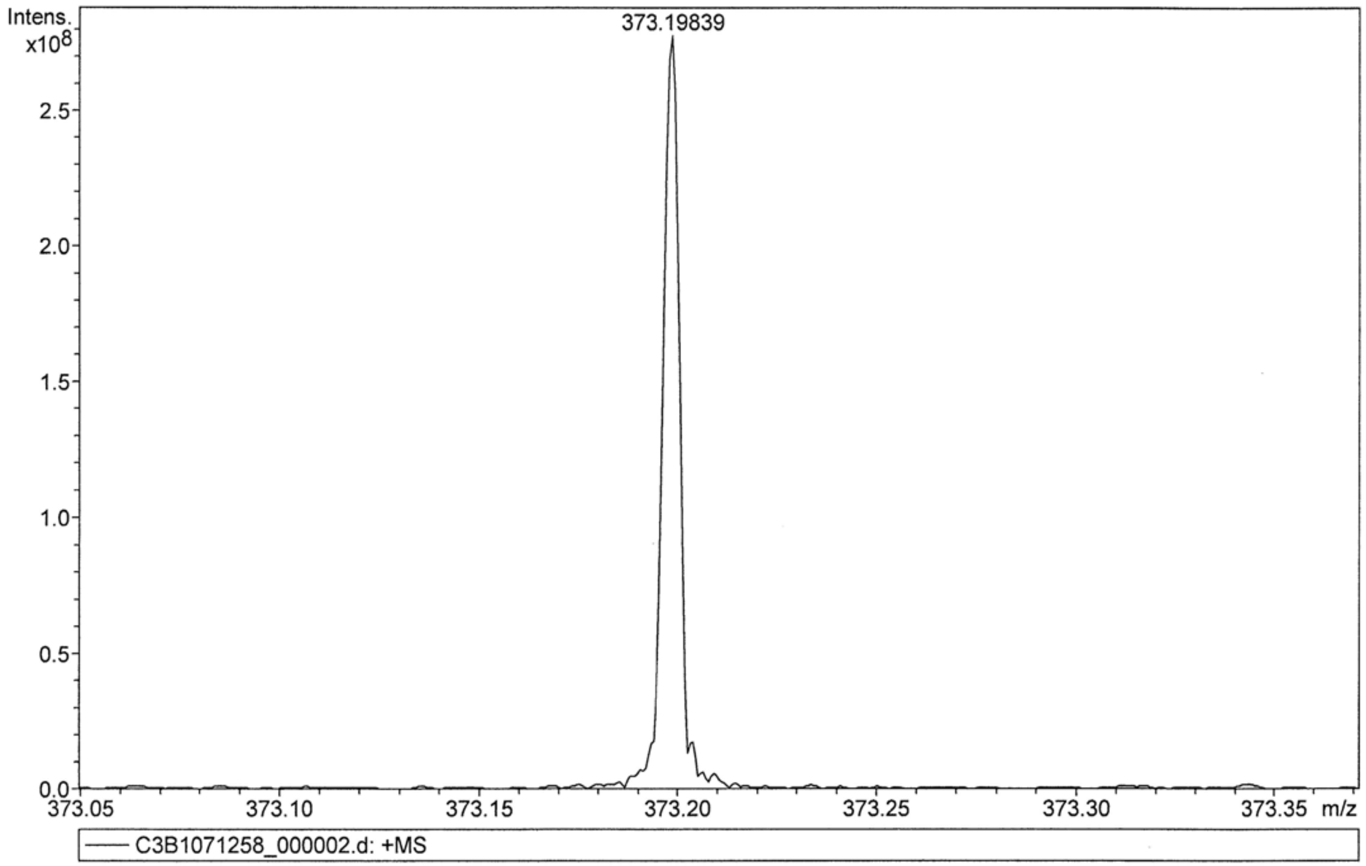

Meas. m/z	#	Formula	Score	m/z	err [mDa]	err [ppm]	mSigma	rdb	e⁻ Conf	N-Rule
373.19839	1	C 20 H 30 Na O 5	100.00	373.19855	0.16	0.43	7.9	5.5	even	ok

Figure S29. HREIMS spectrum of **5**

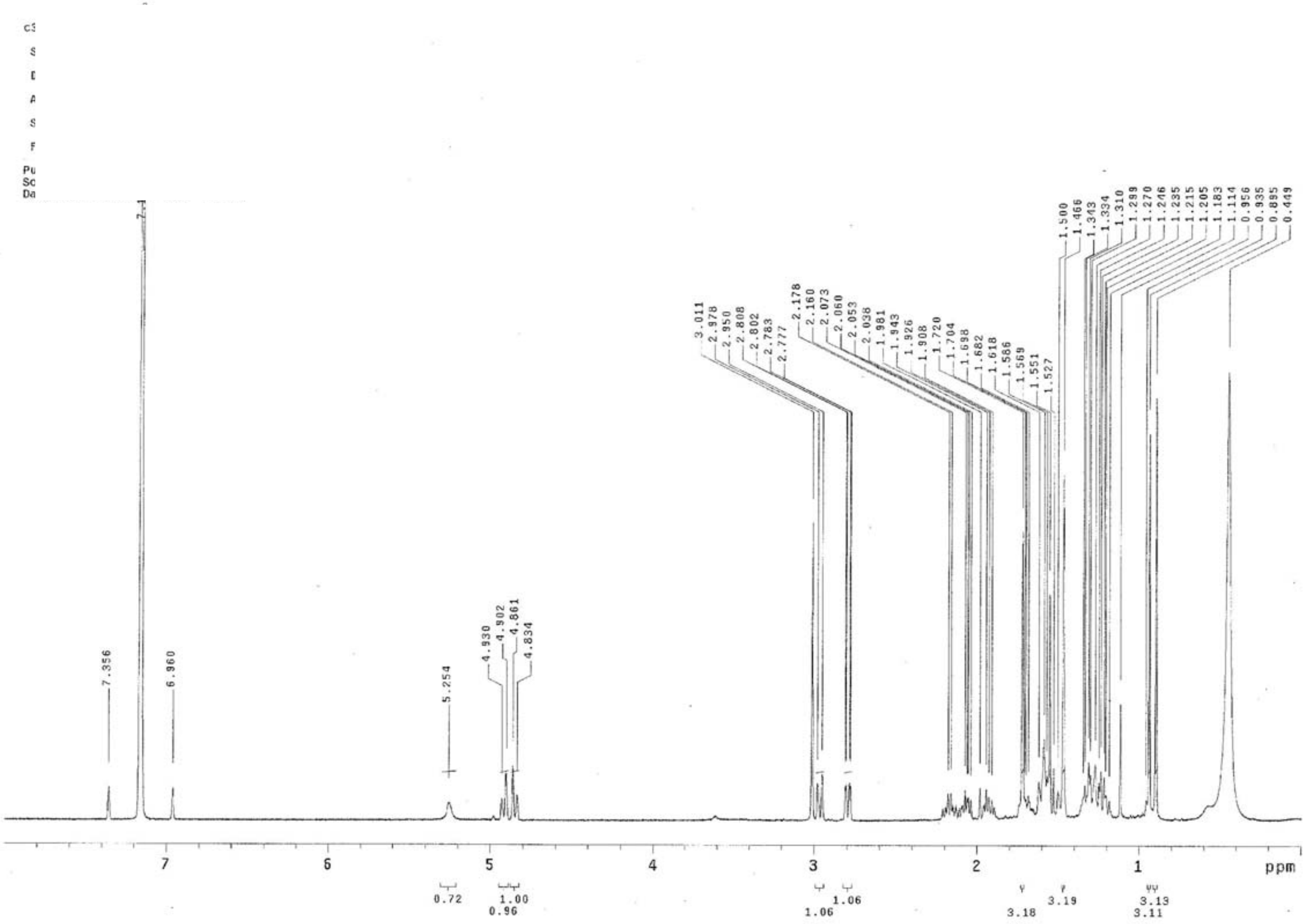

Figure S30. ^{1}H NMR spectrum of **5** in C_6D_6

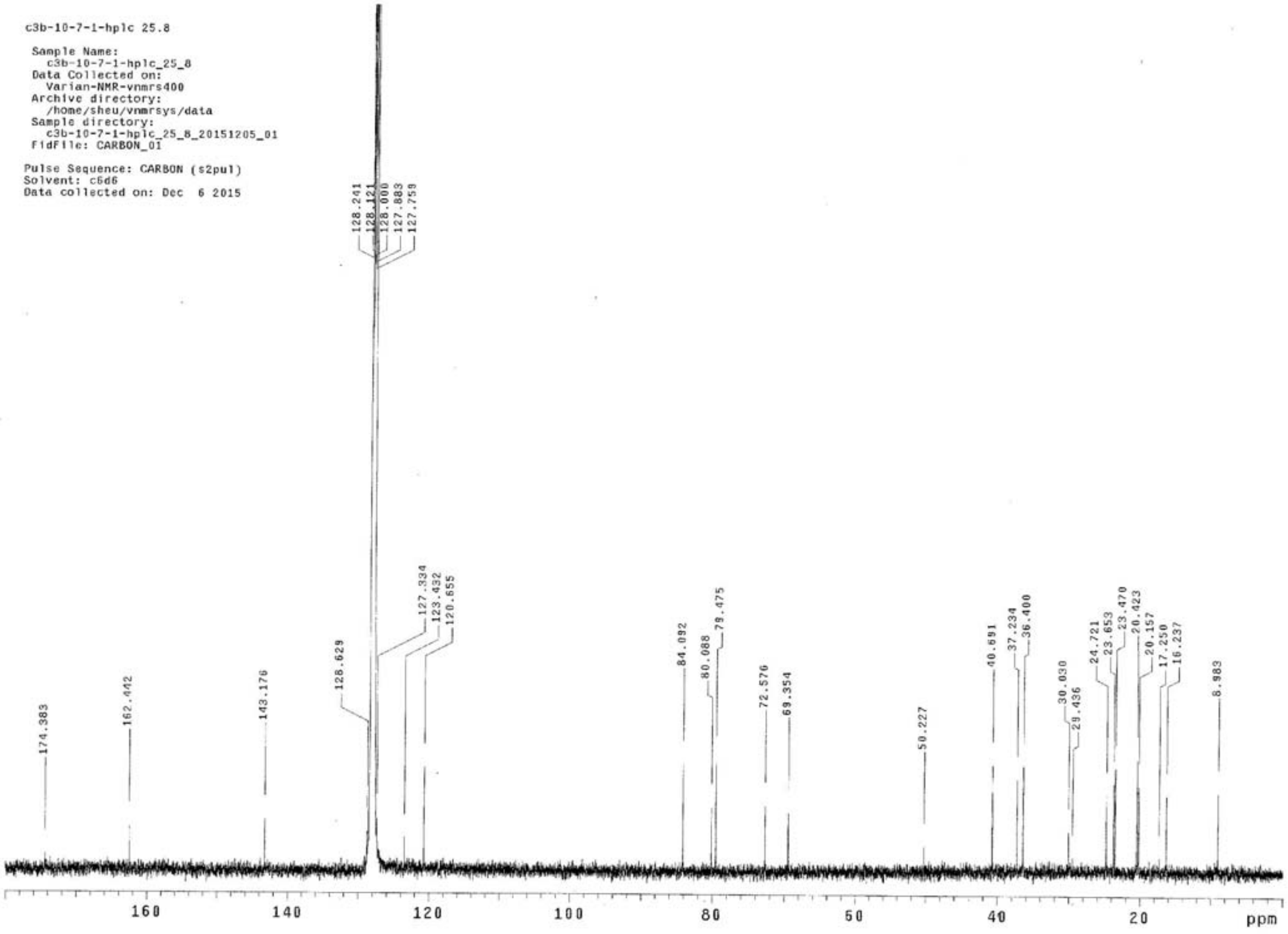

Figure S31. ^{13}C NMR spectrum of **5** in C_6D_6

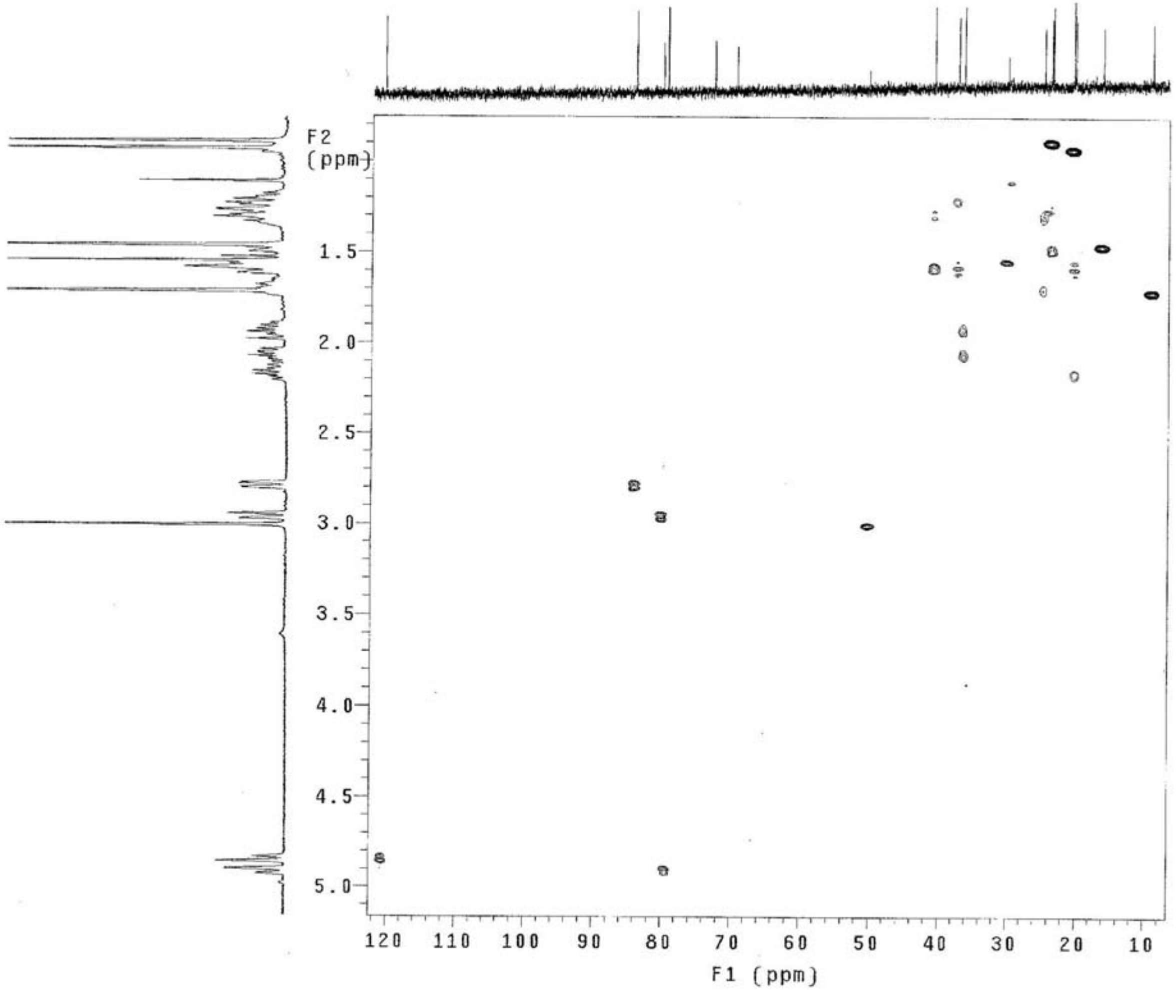

Figure S32. HSQC spectrum of **5** in C_6D_6

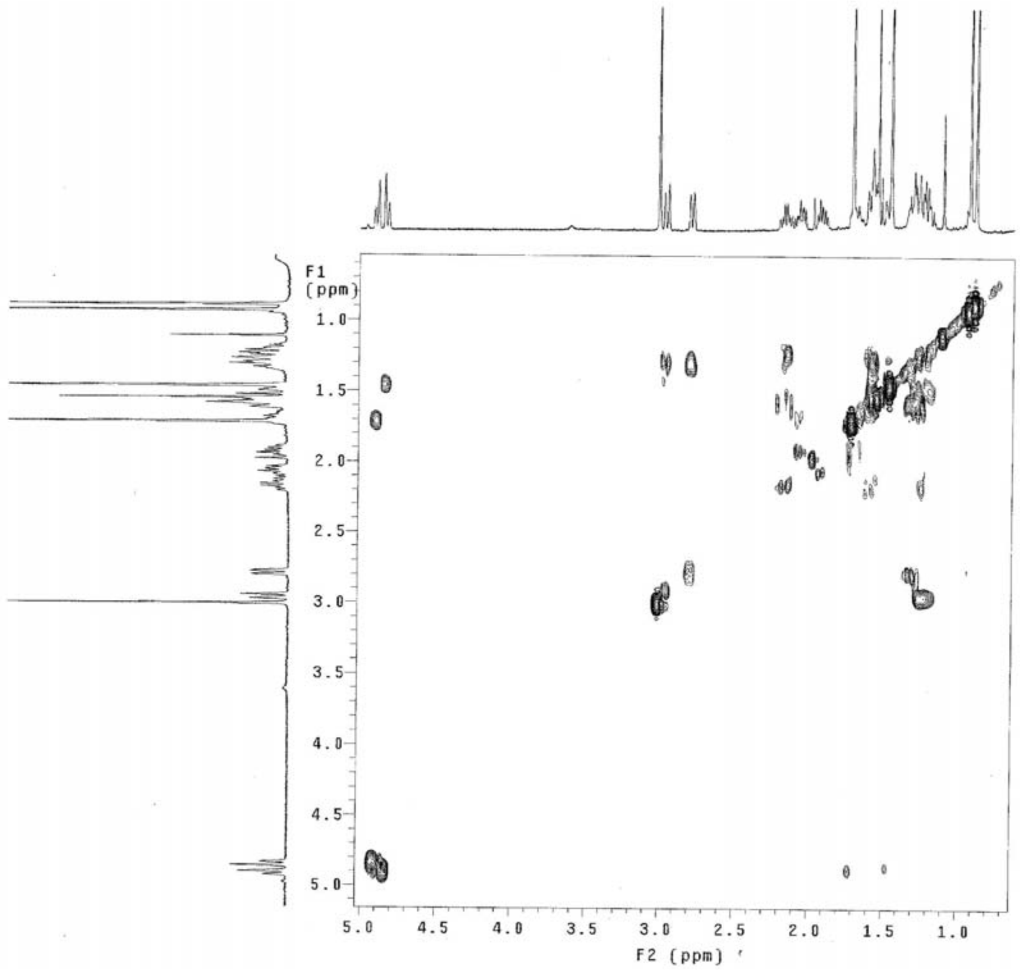

Figure S33. ^{1}H-^{1}H COSY spectrum of **5** in C_6D_6

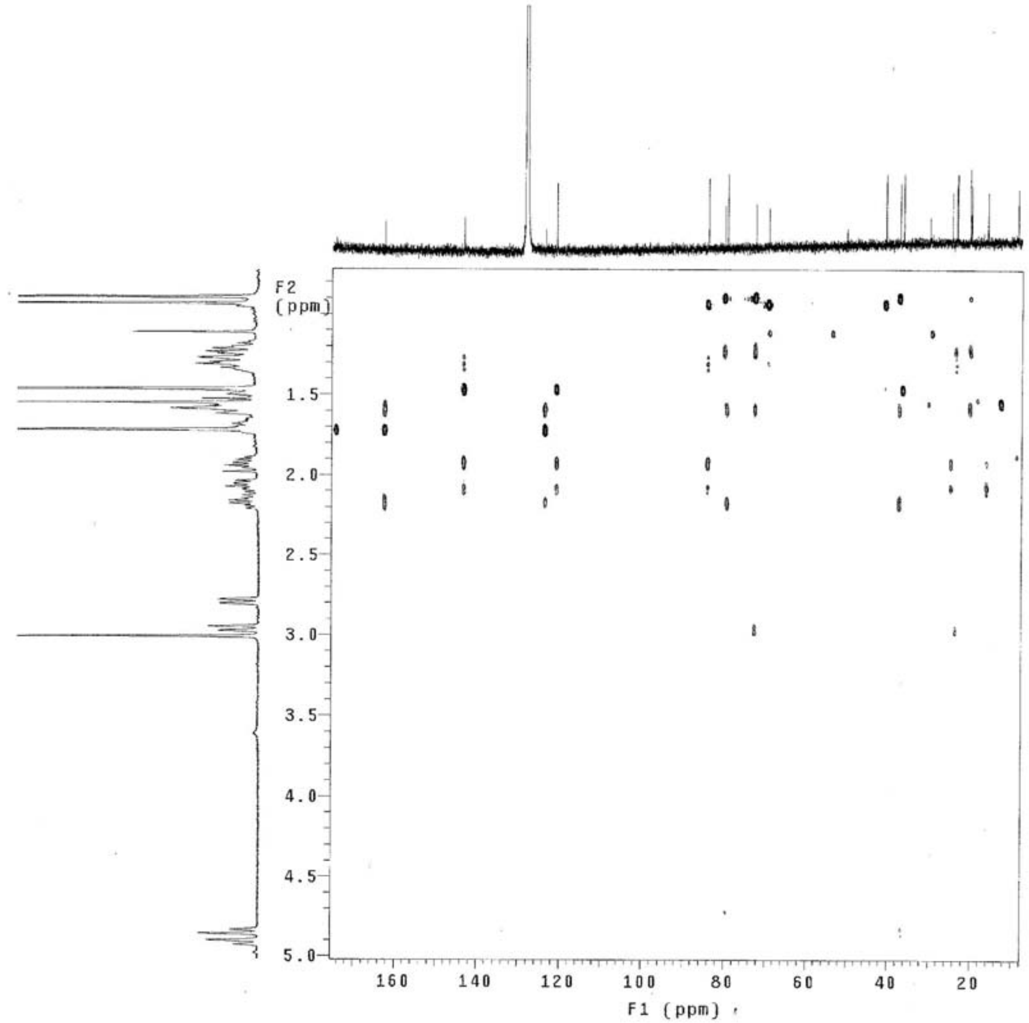

Figure S34. HMBC spectrum of **5** in C_6D_6

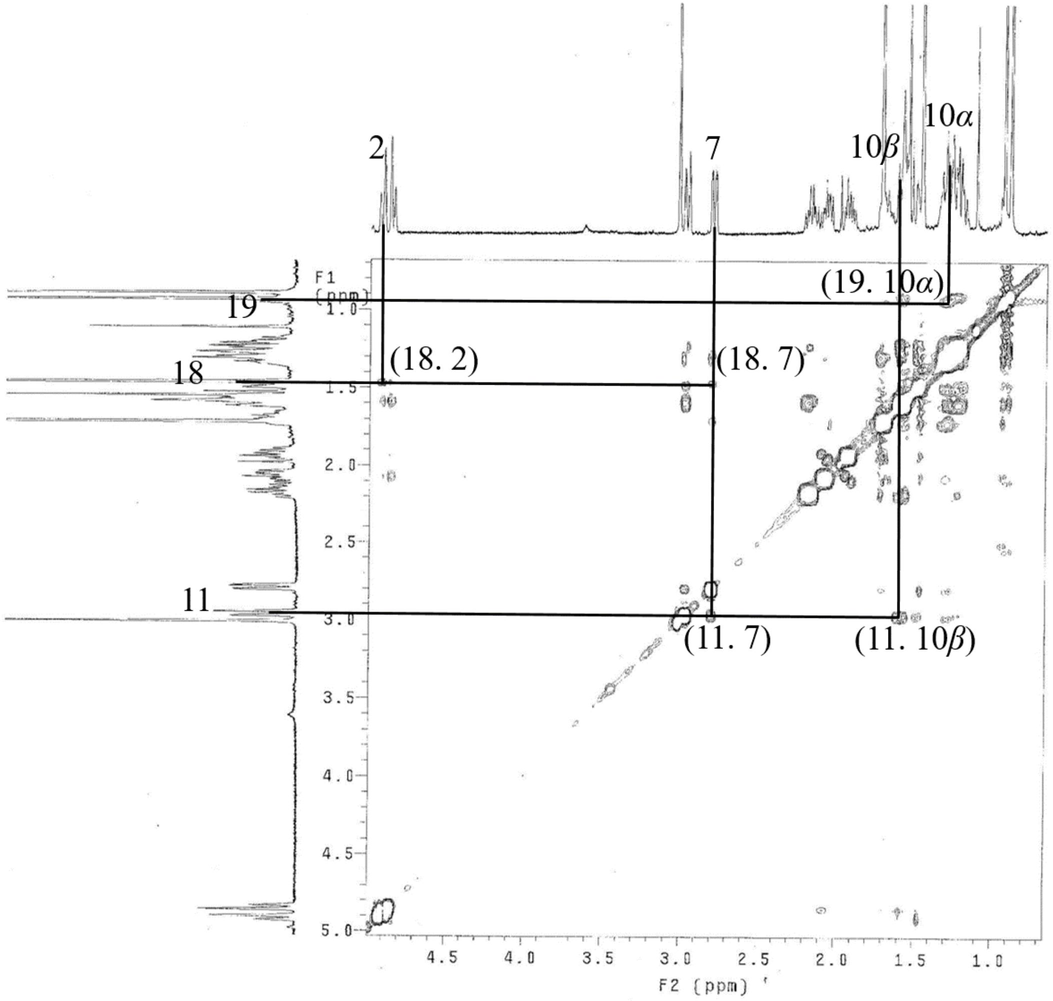

Figure S35. NOESY spectrum of **5** in C_6D_6

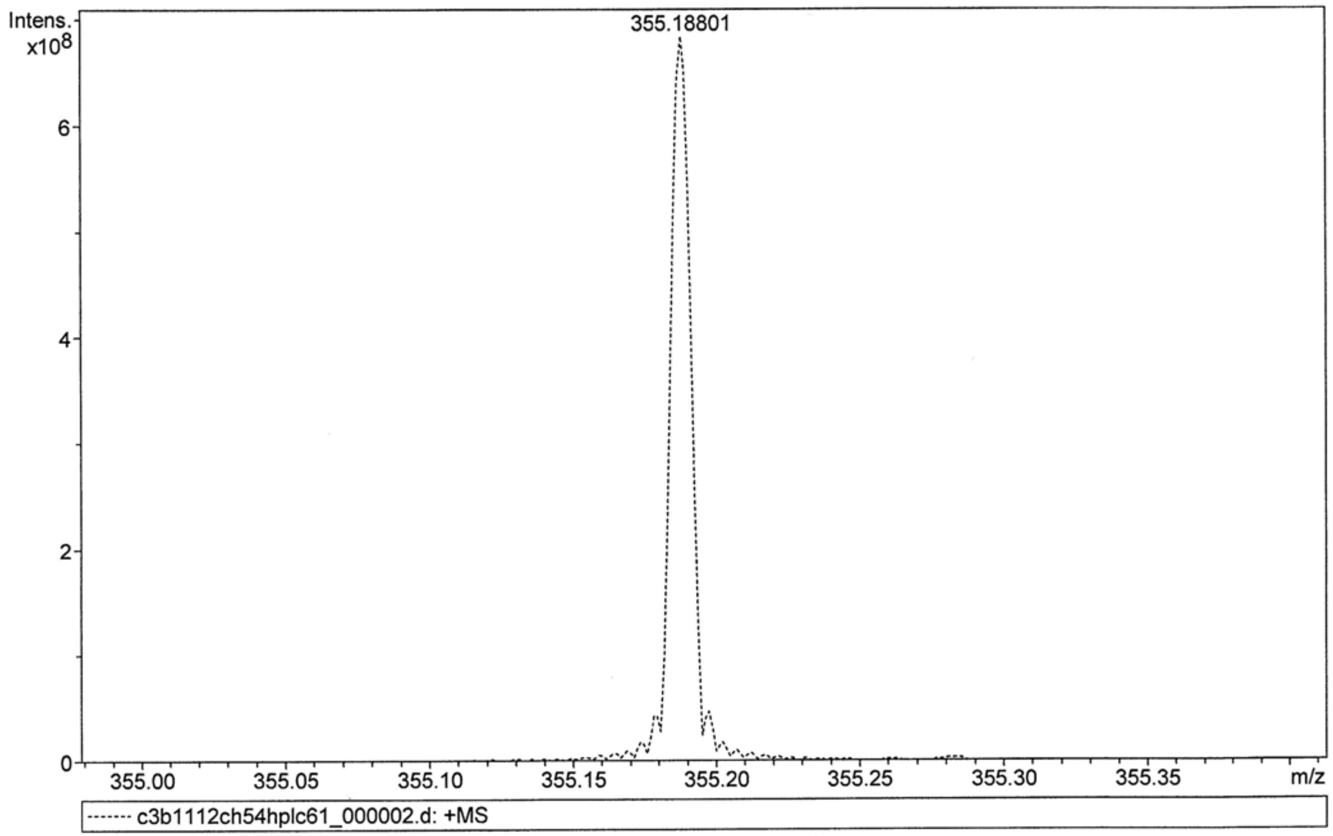

Meas. m/z	#	Formula	Score	m/z	err [mDa]	err [ppm]	mSigma	rdb	e⁻ Conf	N-Rule
355.18801	1	C 20 H 28 Na O 4	100.00	355.18798	-0.03	-0.10	17.9	6.5	even	ok

Figure S36. HRESIMS spectrum of **6**

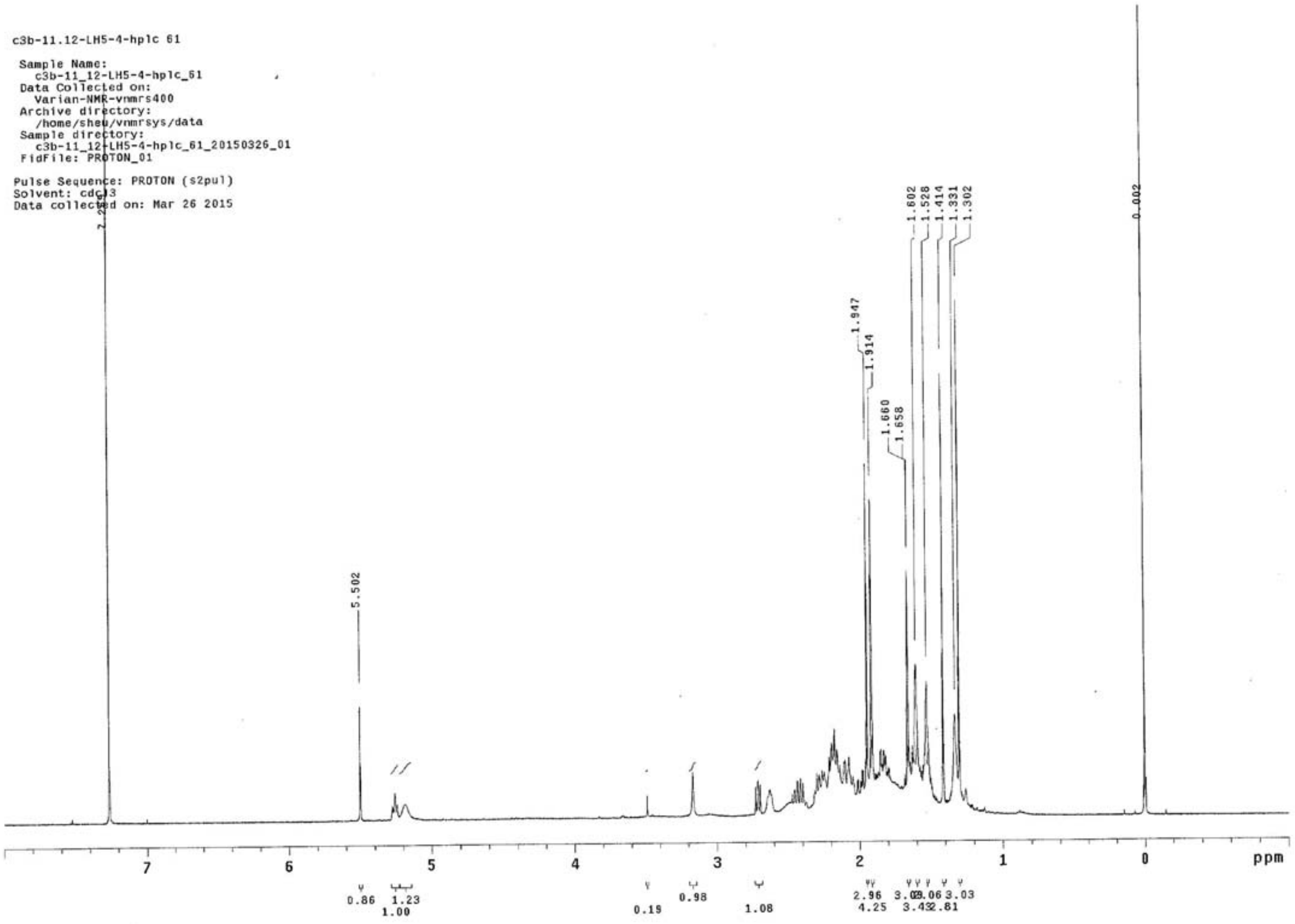

Figure S37. ^1H NMR spectrum of **6** in $CDCl_3$

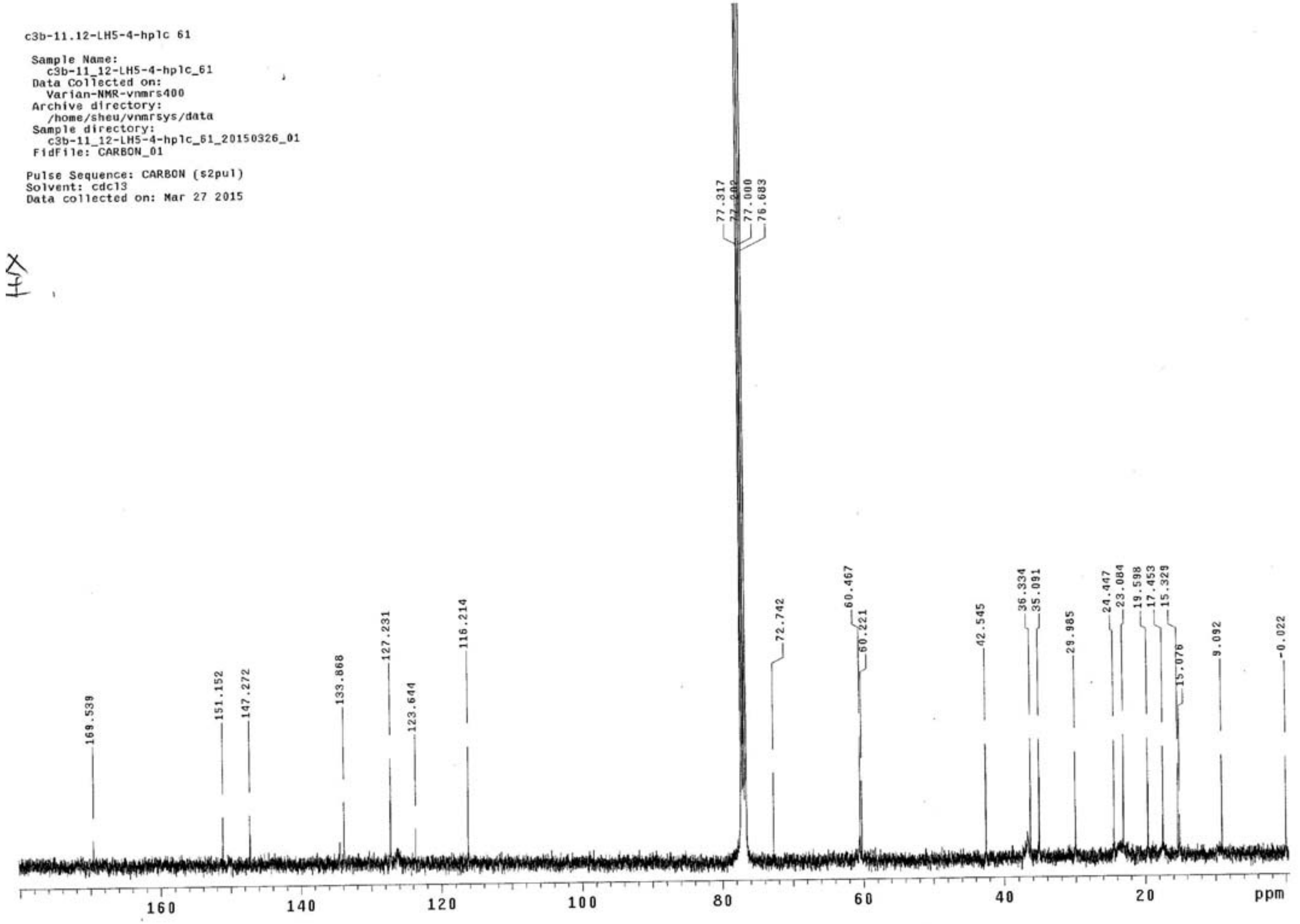

Figure S38. ^{13}C NMR spectrum of **6** in $CDCl_3$

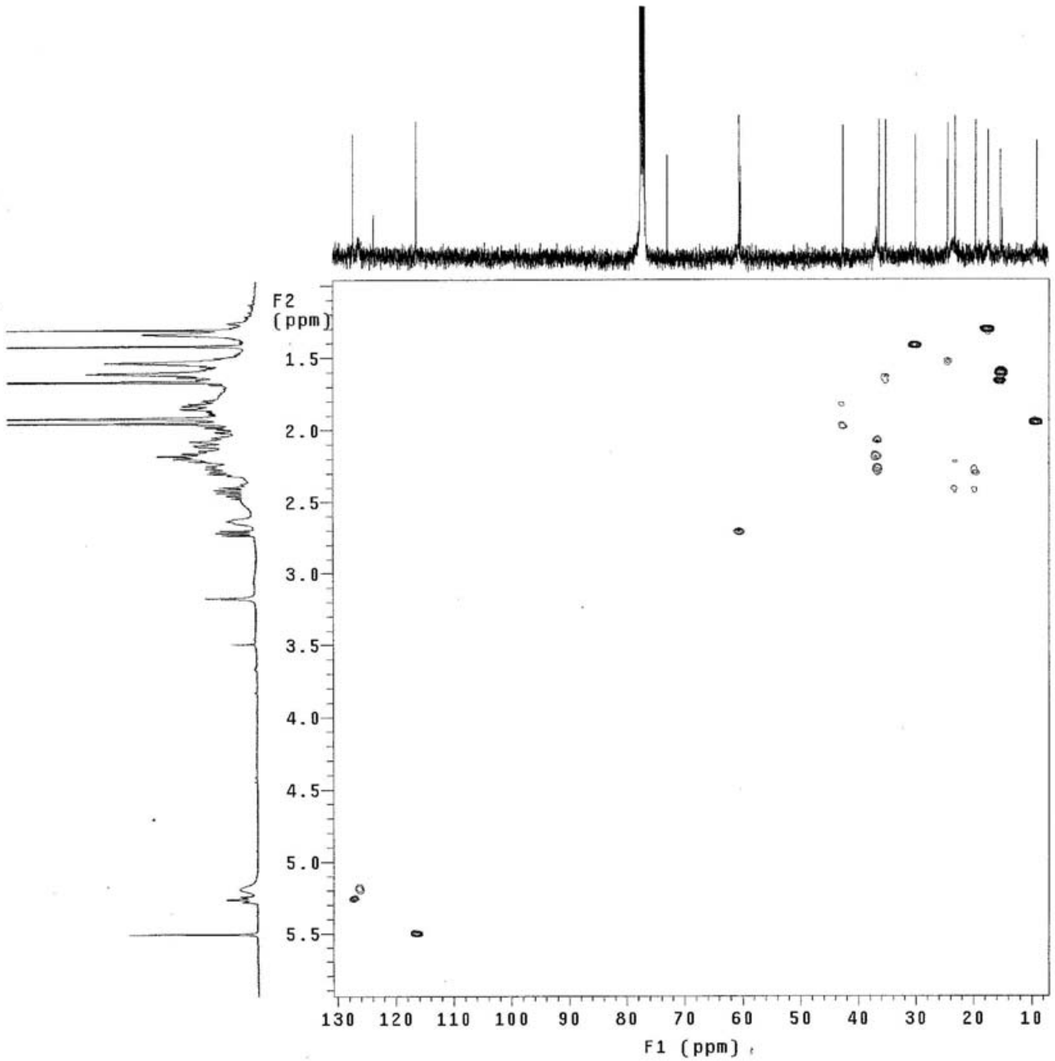

Figure S39. HSQC spectrum of **6** in $CDCl_3$

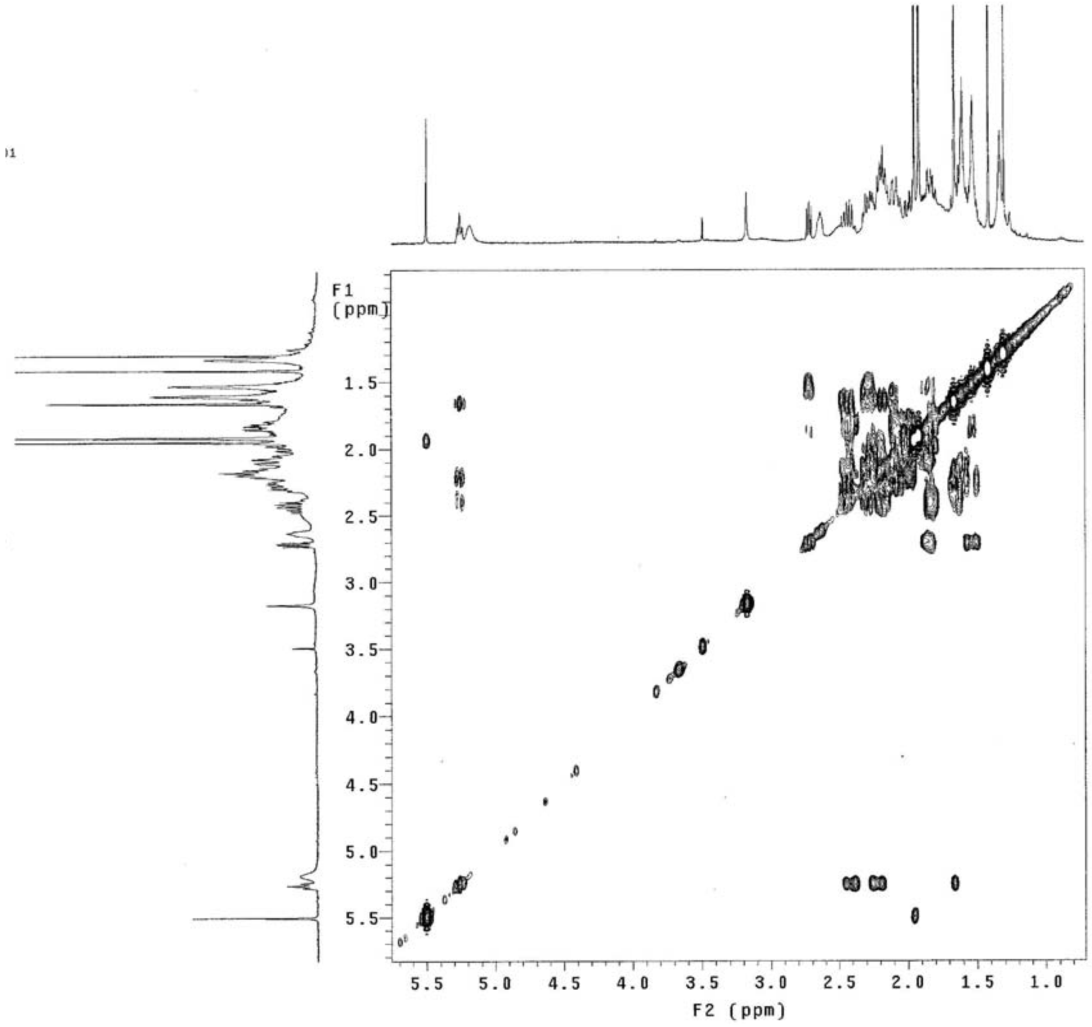

Figure S40. ^{1}H-^{1}H COSY spectrum of **6** in $CDCl_3$

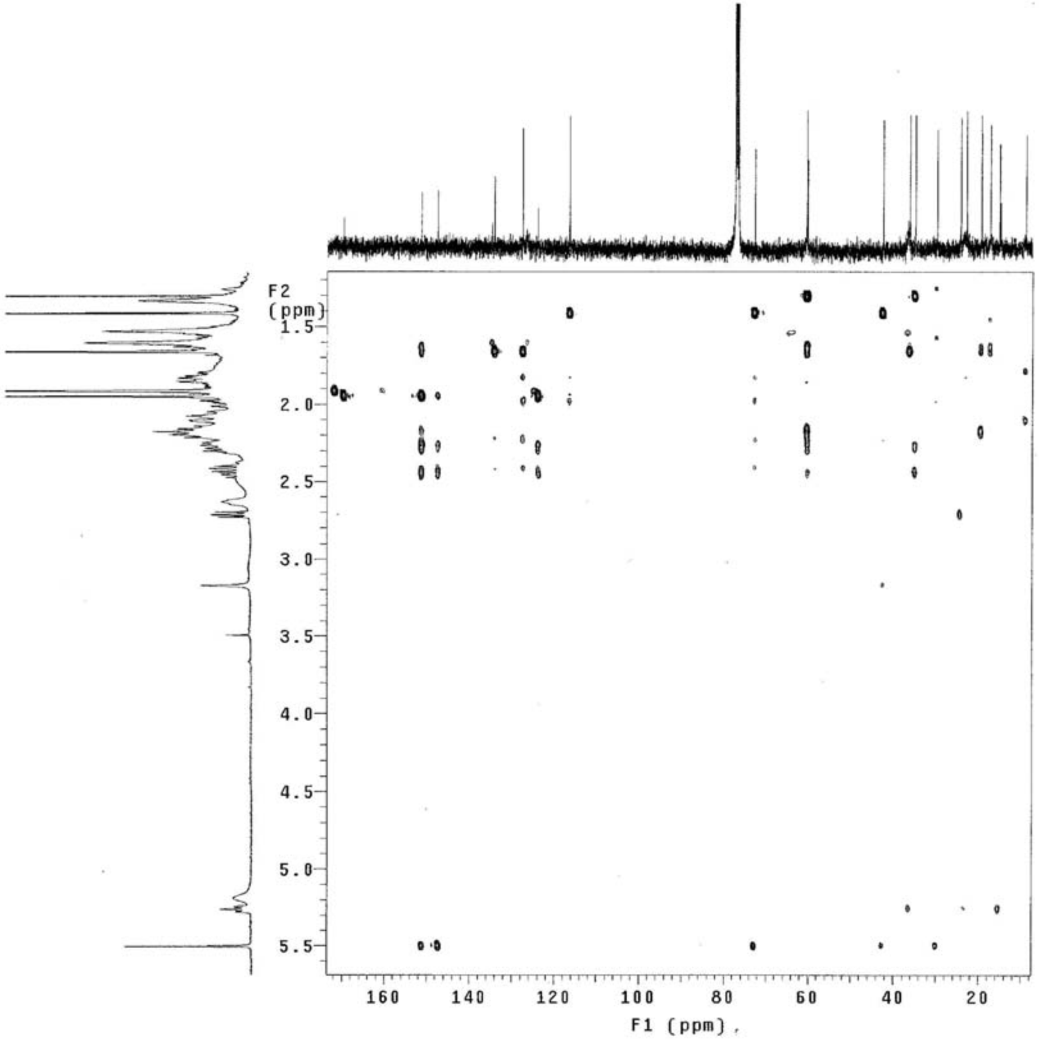

Figure S41. HMBC spectrum of **6** in $CDCl_3$

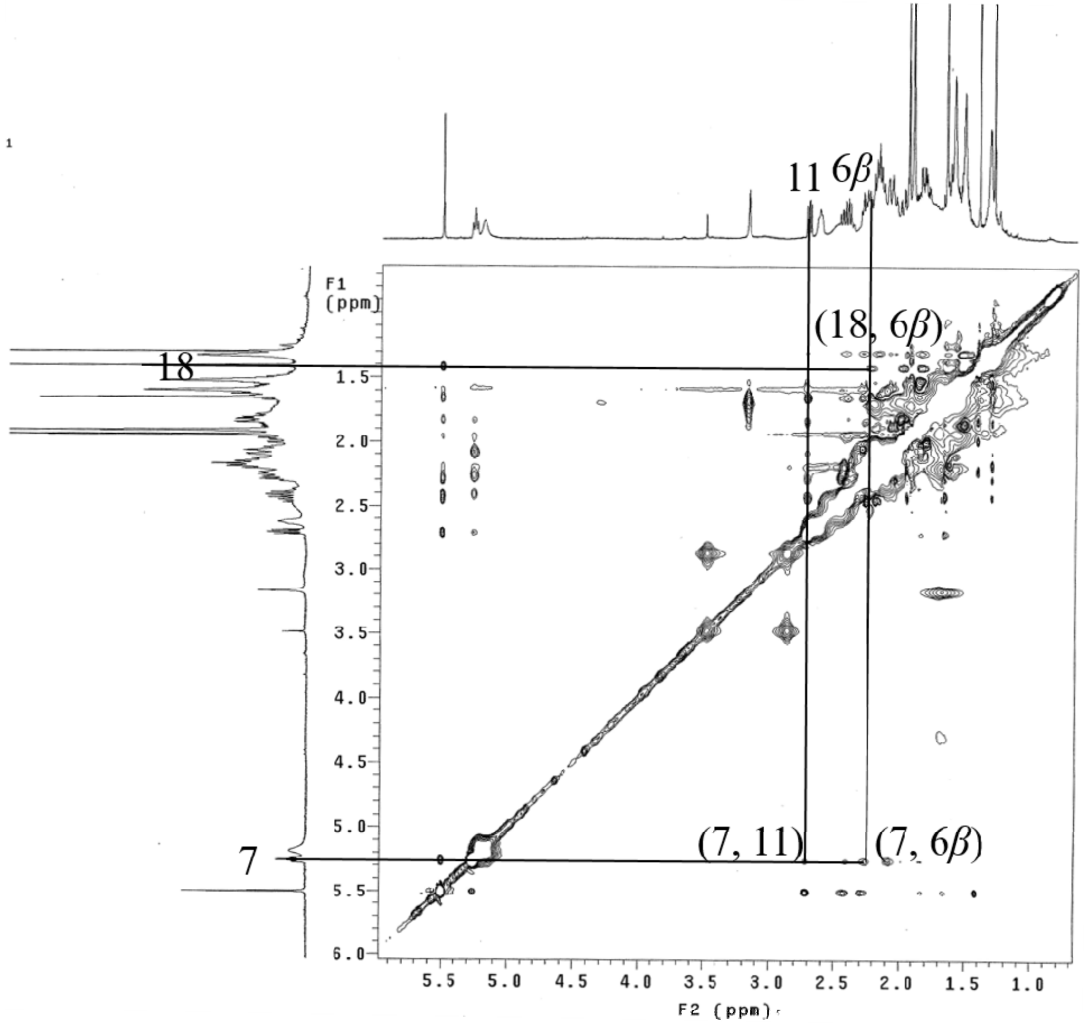

Figure S42. NOESY spectrum of **6** in $CDCl_3$

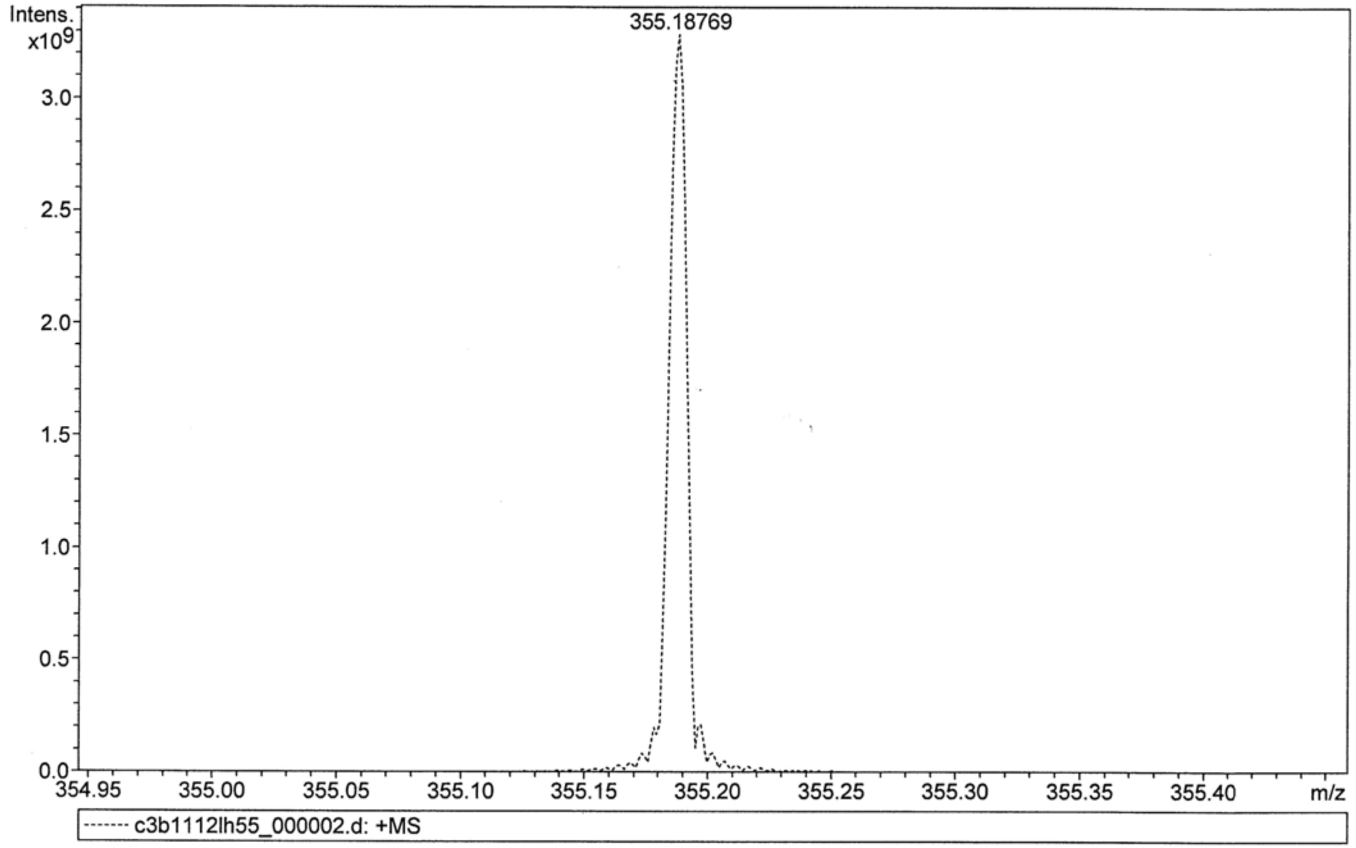

Meas. m/z	#	Formula	Score	m/z	err [mDa]	err [ppm]	mSigma	rdb	e⁻ Conf	N-Rule
355.18769	1	C 20 H 28 Na O 4	100.00	355.18798	0.30	0.83	20.8	6.5	even	ok

Figure S43. HRESIMS spectrum of **7**

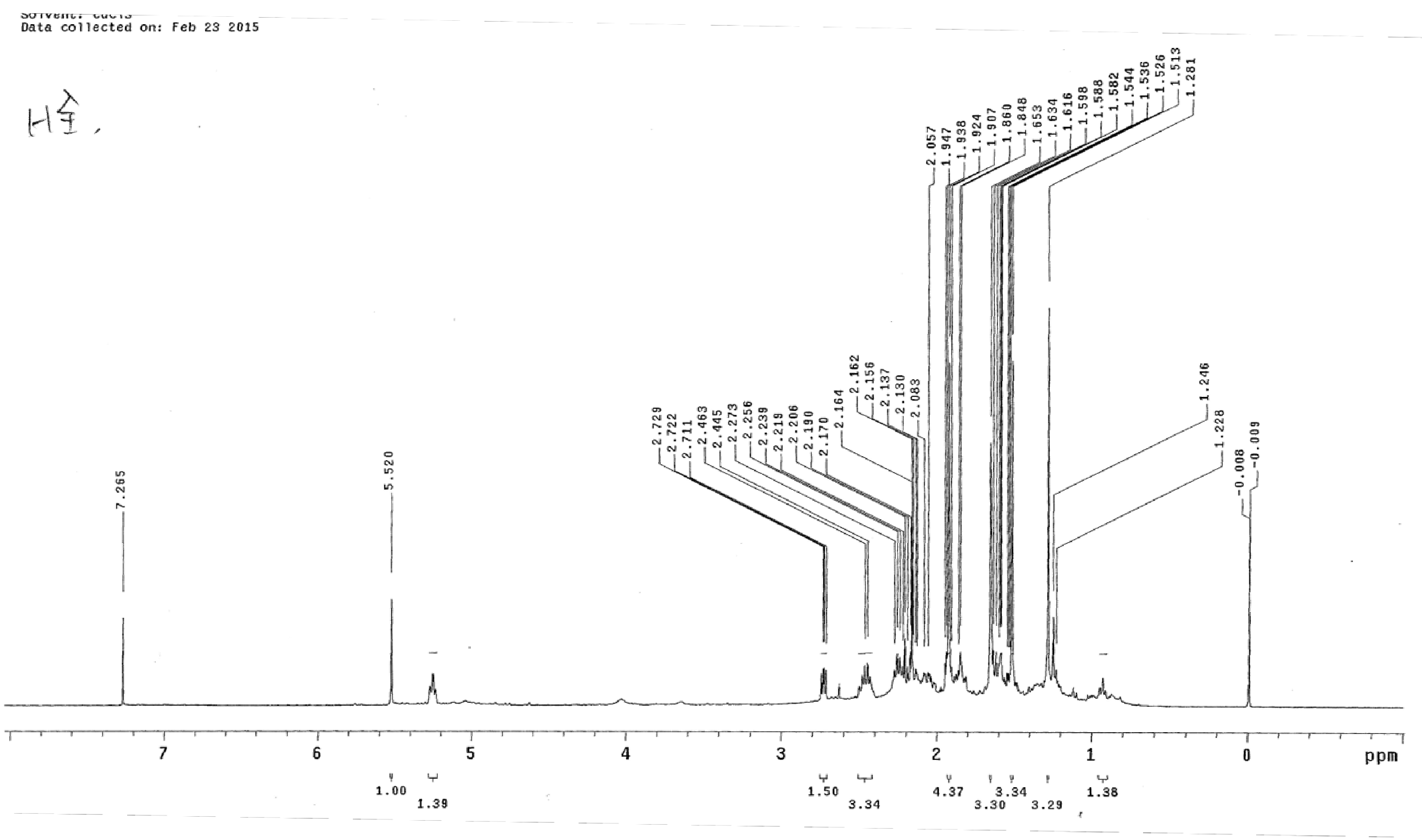

Figure S44. ^{1}H NMR spectrum of **7** in $CDCl_3$

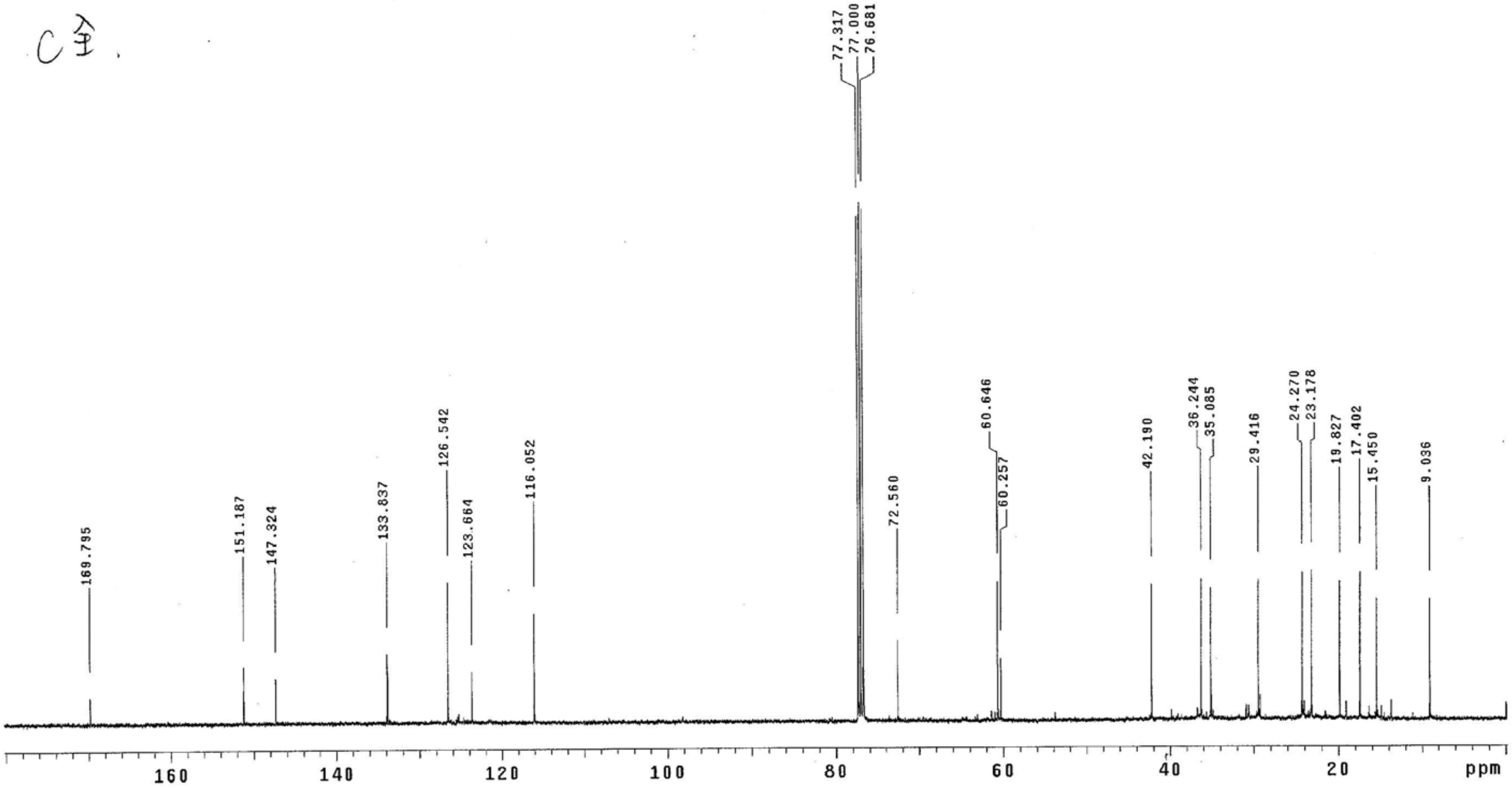

Figure S45. ^{13}C NMR spectrum of **7** in $CDCl_3$

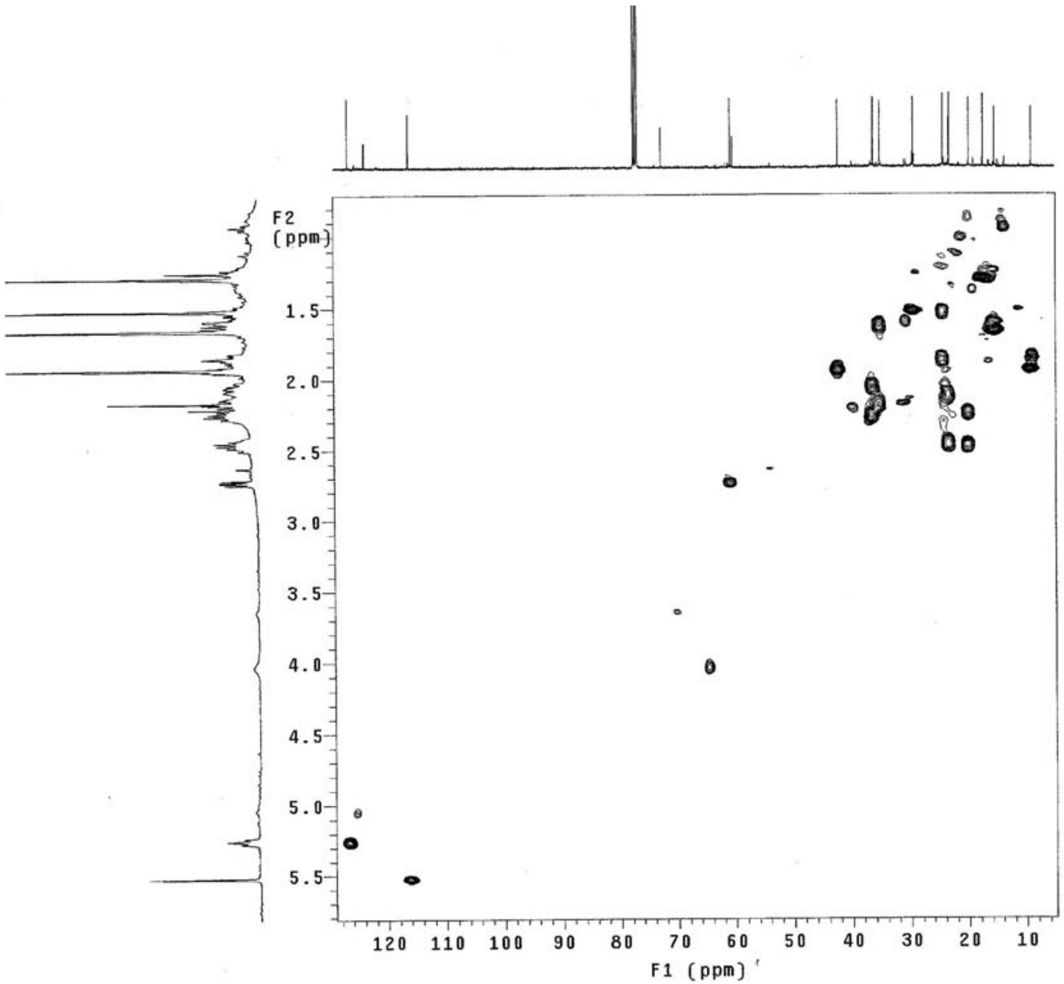

Figure S46. HSQC spectrum of **7** in $CDCl_3$

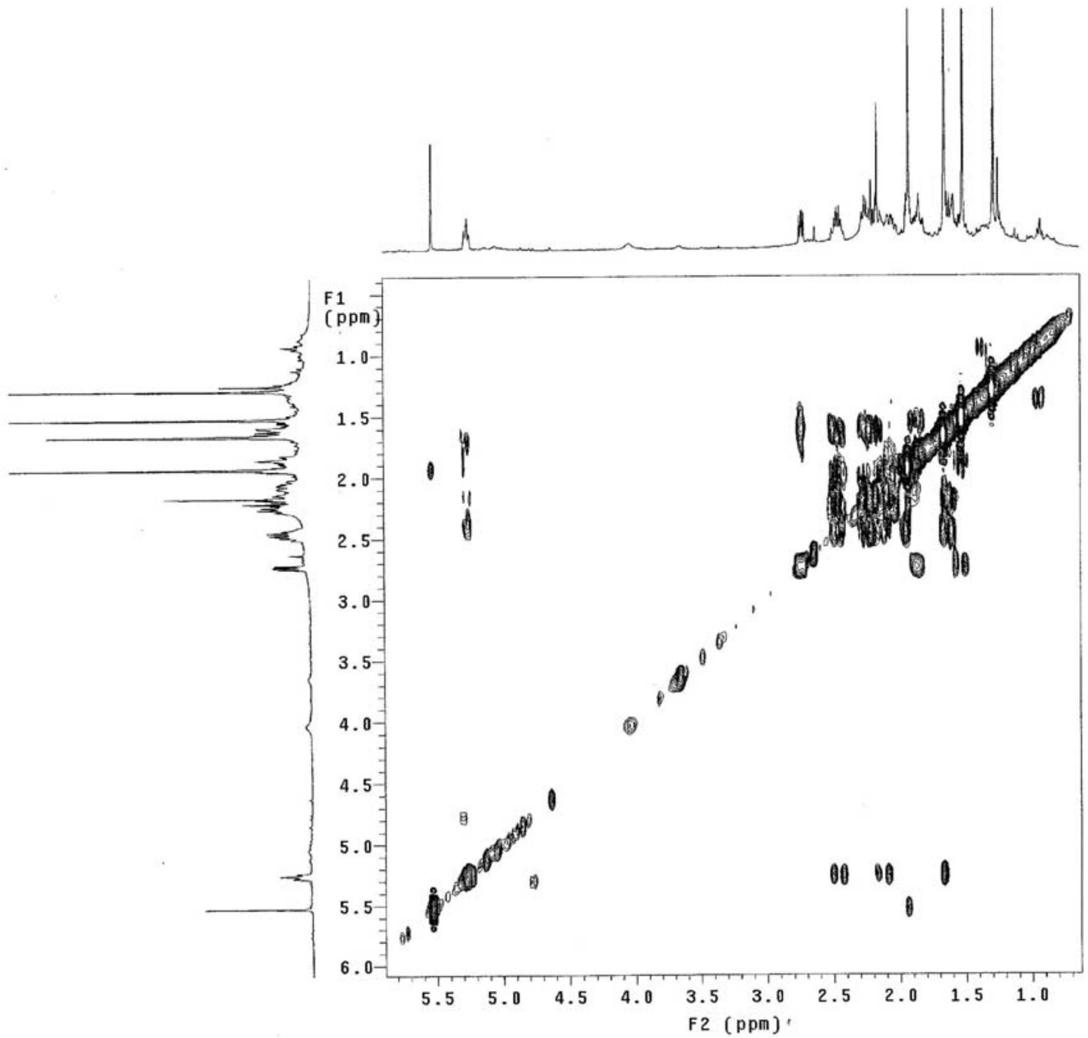

Figure S47. ^{1}H-^{1}H COSY spectrum of **7** in $CDCl_3$

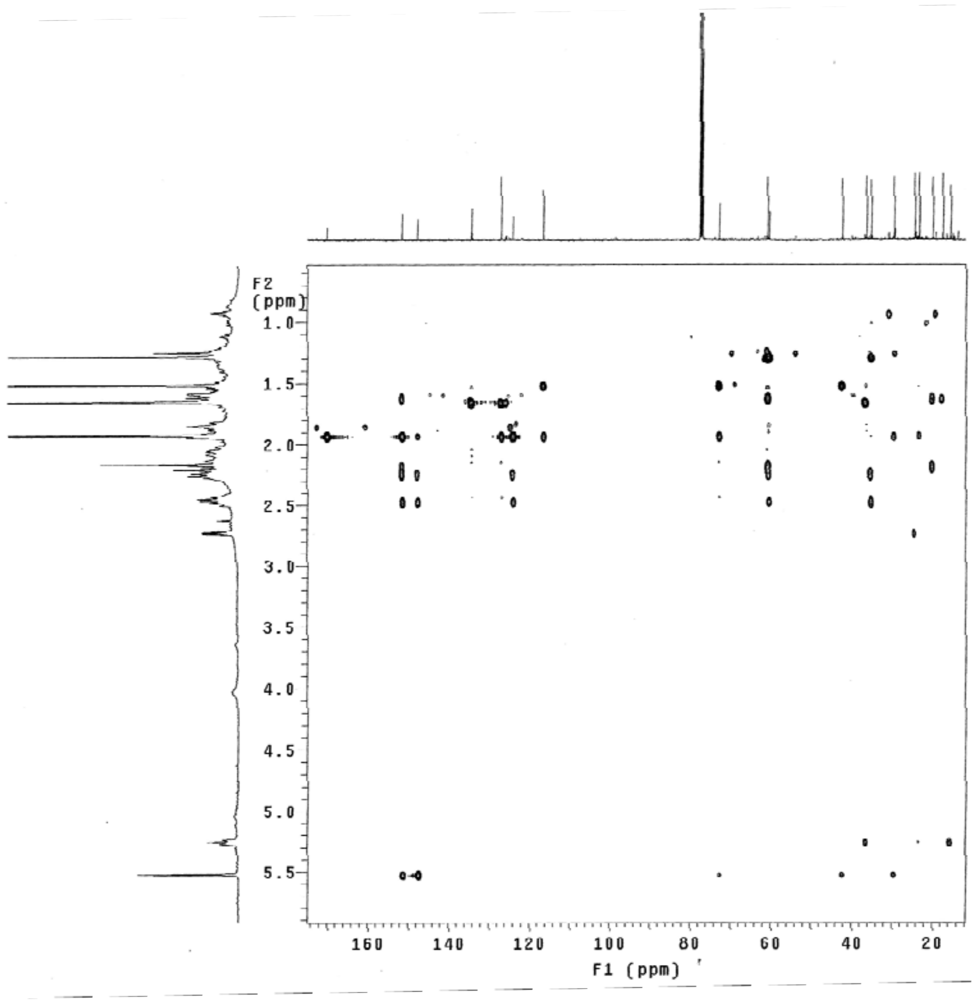

Figure S48. HMBC spectrum of **7** in $CDCl_3$

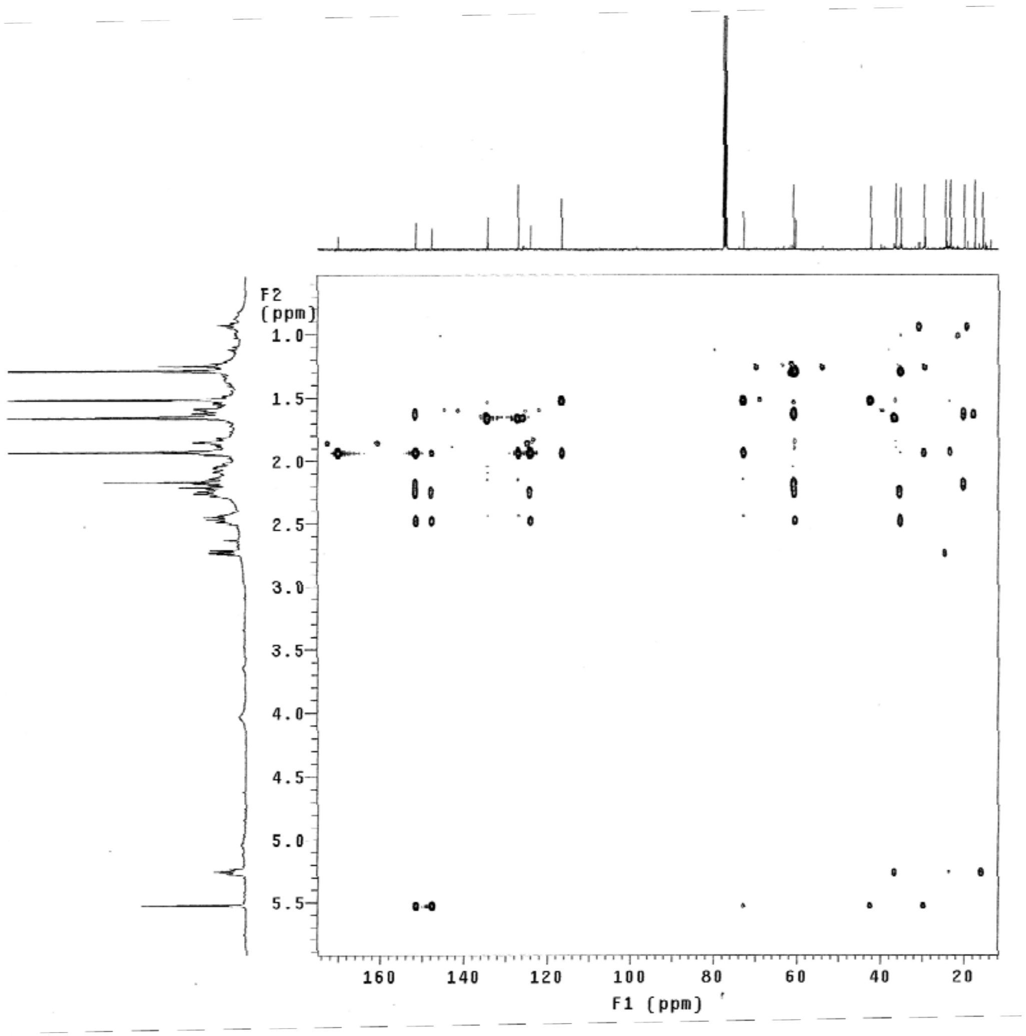

Figure S49. NOESY spectrum of **7** in $CDCl_3$
